# Environmental Determinants of Foraging Site Revisitation by African Elephants ( *Loxodonta africana* )

**DOI:** 10.1002/ece3.71506

**Published:** 2025-06-03

**Authors:** Suzanne Antoinette Jacob, Willem Frederik De Boer, Henrik Johan De Knegt, Cassander Cassijn Engelen, Michelle Henley

**Affiliations:** ^1^ Wildlife Ecology and Conservation Group Wageningen University Wageningen the Netherlands; ^2^ Elephants Alive Hoedspruit South Africa; ^3^ Applied Behavioural Ecology and Ecosystem Research Unit, School of Environmental Sciences University of South Africa Florida South Africa; ^4^ Department of Philosophy, Faculty of Humanities University of Johannesburg Auckland Park South Africa

**Keywords:** African elephant ( *Loxodonta africana* ), density, foraging, Greater Kruger, movement ecology, revisitation

## Abstract

Understanding the spatial patterns of herbivore habitat use is important for understanding ecological processes and ecosystem management. Past research has mainly focused on explaining herbivore habitat selection, to the extent that environmental variables driving habitat selection can be considered well researched. However, little is known about how environmental variables contribute to patterns of foraging site revisitation, even though revisited sites might represent sites of ecological significance. The increasing elephant population in South Africa is causing a decline in woody species, necessitating insight into elephant habitat use to improve the management of areas in which they occur. This study aimed to gain insight into the spatial patterns of foraging site revisitations by the African elephant ( *Loxodonta africana* ). To achieve this, the non‐randomness of the spatial locations of revisited sites in terms of their environmental conditions was assessed. Next, the relations between these environmental variables and the revisitation rate per site were analysed. Lastly, a model was constructed to assess which environmental variables had the largest effect on site revisitations. Ten years of GPS tracking data of elephants in the Greater Kruger Area, South Africa, was used. Environmental variables were obtained from existing data sources. The data were analysed with Kolmogorov tests and linear mixed effects models, using AIC‐based model selection. The results indicate that elephants select revisitation sites close to water and at intermediate slopes. Cows also selected revisitation sites with intermediate phosphorus concentrations. Yet, elephants revisited sites at intermediate distances from water and higher phosphorus concentrations more frequently. Moreover, phosphorus is the most important environmental variable influencing the revisitation rate. This study shows that analysing revisitation patterns gives valuable insight into how animals use specific sites within their home range, as the environmental covariates related to the selection of the revisitation sites differ from those related to the revisitation rate.

## Graphical abstract

This study examines the spatial patterns of foraging site revisitations by African elephants ( *Loxodonta africana* ) in South Africa's Greater Kruger Area. Using 10 years of GPS tracking data and environmental variables, it reveals that elephants select revisitation sites near water and revisit sites with higher phosphorus concentrations. The findings highlight how revisitation patterns provide unique insights into habitat use, differing from general site selection, with implications for managing protected areas where elephants are present.

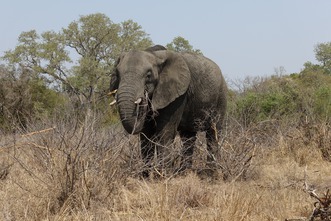

## 1 Introduction

Deciding where and when to forage is crucial for the survival of animals. These decisions do not only impact the animals themselves, but also other components of the ecosystem. Large herbivores, for example, can influence ecosystems by altering vegetation biomass and structure, which in turn has cascading effects on other species (Adler et al. 2001; Côté et al. 2004). Understanding the spatial patterns of habitat use by herbivores is thus important for understanding ecological processes, biodiversity conservation and ecosystem management. The environmental factors that influence habitat selection are nowadays well understood, such as food quality and availability (Van Beest et al. 2010) and predation risk (Gaynor et al. 2019; Moll et al. 2017). Despite this, little is known about how environmental variables contribute to patterns of foraging site revisitations, even though frequently revisited sites might be sites of high ecological significance. Animals often return to sites containing regularly used resources like foraging patches, nests, dens, watering holes, etc. (Bracis et al. 2018). Consistent revisitation of foraging sites has been recorded for multiple species (McLaren and Patterson 2021; Rebstock et al. 2022; Schloesing et al. 2020). For example, gorillas frequently return to foraging patches with high food abundance and quality (Watts 1998). Likewise, elk were found to revisit highly productive foraging patches regularly (Seidel and Boyce 2015). In some cases, animals even keep revisiting the same areas when these are of poor quality, while more profitable areas are available (Merkle et al. 2015). Despite this, revisiting sites, even when of poor quality, might still prove beneficial, as site fidelity can increase reproductive success by increasing foraging efficiency (Rebstock et al. 2022).

African elephants ( *Loxodonta africana* ) tend to return to foraging sites, more than could be expected from random movements (De Knegt 2010). Elephants are able to display this behaviour, as they are a long‐lived species that excel in ‘long‐term, extensive spatial‐temporal and social memory’ (Hart et al. 2008, 86). They should thus be able to remember the ‘what’, ‘where’ and ‘when’, which are essential prerequisites for revisiting sites (Eacott and Easton 2010). This species is therefore an excellent species to study site revisitation.

There are multiple reasons why elephants visit specific sites; one of them is the presence of water (Viljoen 1989). Although these visitations happen so that an elephant can drink, distance to water is also important for foraging, as elephants are restricted in their movements by water during the dry season (Polansky et al. 2015). Consequently, due to the high elephant density near waterbodies, woody vegetation near water is browsed upon more by elephants (De Beer et al. 2006). Regarding foraging sites, elephants also select areas with low slopes and at large distances from anthropogenic influences (Mpakairi et al. 2019; Roever et al. 2012; Wall et al. 2006). Elephants also consistently select greener‐than‐average vegetation throughout the year (Loarie et al. 2009) and prefer sites with a high productivity and a high grass biomass (Tsalyuk et al. 2019). Moreover, patches that received more rain might have more and better forage, such as fruits (Danquah and Oppong 2007). Pretorius et al. (2012) found that elephants maximise phosphorus intake throughout the year, nitrogen intake during the wet season and energy intake during the dry season. Hence, elephants have smaller home ranges in phosphorus‐rich areas, which may imply more frequent revisitations (Sach et al. 2020). Additionally, phosphorus has been shown to positively influence debarking behaviour in elephants (Ihwagi et al. 2012), suggesting that phosphorus availability may affect various aspects of elephant foraging behaviour. In addition, large mammals tend to avoid open areas during hot periods, as thermoregulation is essential for them (Valeix et al. 2008), and elephants therefore spend more time in shaded areas (Duffy et al. 2011). So, the factors influencing habitat selection by elephants are well known.

Compared to habitat selection, revisitations might be driven by a more extreme selection of sites, as certain visited sites might be assessed as being of such high value that the elephants keep returning to those sites, thus revisiting the sites with an even higher quality than the selected habitat patches. Additionally, elephant‐vegetation feedback loops might play a role in driving the patterns of site revisitation, as understory biomass is found to be greater at sites where elephants are present (Coverdale et al. 2016). However, how these variables influence site revisitations is not sufficiently understood. Moreover, the African elephant is a key species in African savannas through altering the vegetation and thereby the herbivore community composition (Skarpe et al. 2014). Identifying landscape variables that drive revisitation patterns can have important implications for conservation, as protecting sites with high ecological significance can benefit both the conservation of the African elephants themselves as well as other species they interact with.

**FIGURE 1:**
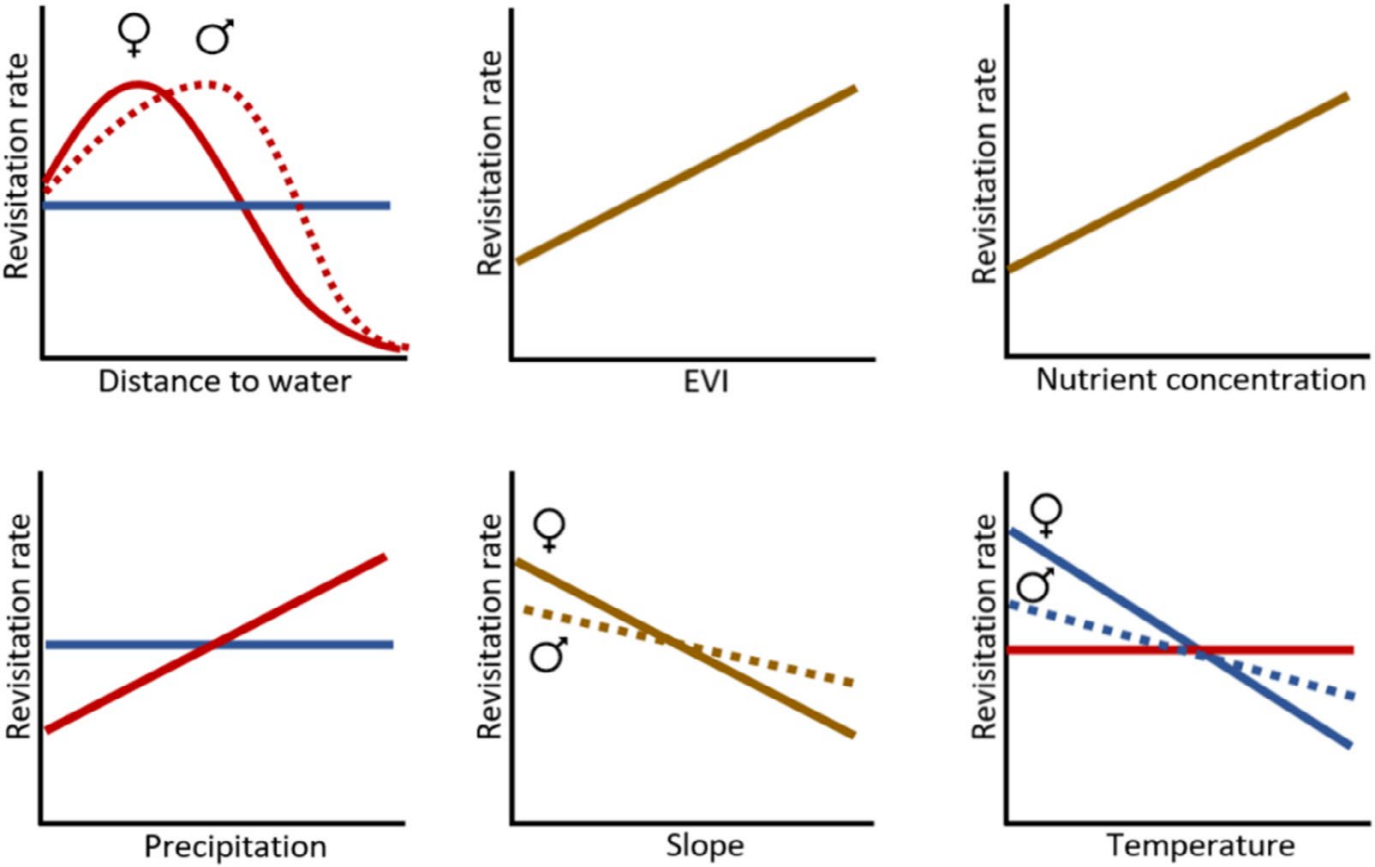
Hypothesised relations between the environmental variables and the revisitation rate per site, for the dry season (red line) and wet season (blue line) or year round (brown line). When a difference between cows and bulls is hypothesised, the cows are represented by a solid line and the bulls by a dashed line.

In this paper, we analyse the spatial patterns of site revisitation by African elephants in the Greater Kruger National Park, South Africa. We do so by first assessing the non‐randomness of the spatial locations of revisited sites in terms of their environmental conditions. Next, we analyse the relations between environmental variables and the number of revisitations per site. Lastly, we test which of the environmental variables has the strongest correlation with the patterns of site revisitation. Since waterbodies are known to be frequently revisited, and the focus of this paper is on vegetation‐based site revisitations, revisitations of waterbodies are excluded from the analyses. Similar to the patterns of habitat selection, we expected that the elephants revisit sites that are greener, have higher phosphorus and nitrogen concentrations and have lower slopes than the available sites. In addition, we expected the elephants to revisit sites that are close to permanent waterbodies and have high precipitation during the dry season, and that have low temperatures during the hot wet season. Yet, unlike general habitat selection, we also expected a correlation between the revisitation rate and environmental variables, since we expected the patterns of site revisitations to be more extreme than those of general habitat selection. For example, we expected that elephants revisit sites most frequently that have the highest phosphorus concentrations (Figure 1). Since habitat selection factors differ between bulls/bachelor herds and cows/family units (Evans and Harris 2012; Greyling et al. 2004), as for example, cows stay closer to water and shade due to their smaller size and the presence of young (Stokke 1999), and Greyling et al. (2004) found feeding distinctions between the sexes within the area of this study, we took this into account as well. The relation between the distance to water and the revisitation rate is predicted to follow a quadratic relationship, since high elephant densities near water generate less favourable circumstances close to permanent waterbodies, but elephants cannot move too far from the waterbodies either. Phosphorus is hypothesised to be important for revisitations due to the deficiency of this nutrient in this region (Greyling et al. 2004; Pretorius et al. 2012).

## 2 Material and Methods

### 2.1 Study Area

**FIGURE 2:**
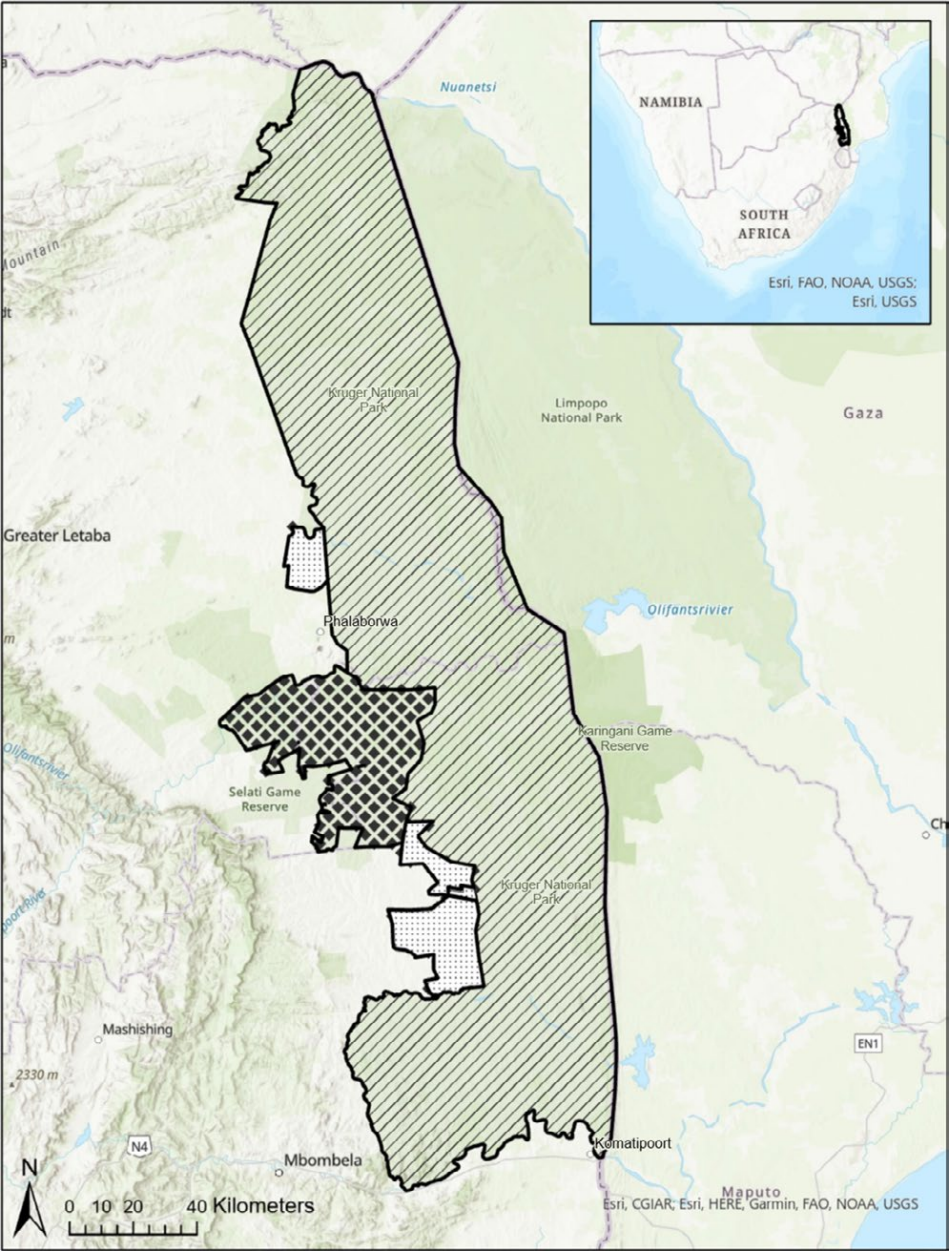
Study area, consisting of KNP (diagonal lines), the APNR (squares) and adjacent private nature reserves (tiny dots).

This study was conducted in the Greater Kruger National Park (Figure 2), South Africa. Kruger National Park (KNP) is one of the largest game reserves in South Africa, spanning over 19,623 km^2^ (South African National Parks, n.d.). The area has a hot semi‐arid climate, with a wet season from November to April and a dry season from May to October (MacFadyen et al. 2018).

### 2.2 Data Collection

Elephant tracking data, collected between January 2012 and October 2021, was provided by the South African‐based Not‐for‐Profit Company, Elephants Alive. The tracking data contained GPS coordinates of 41 elephants, 29 bulls and 12 cows on an hourly scale. Spatial layers on permanent water bodies (artificial waterholes, rivers, etc.) were also provided by Elephants Alive (Table 1). Data layers on the Enhanced Vegetation Index (EVI, an indicator for the greenness of vegetation), soil nutrient content, slope, precipitation and temperature (Table 1) were obtained through Google Earth Engine (GEE) (Gorelick et al. 2017).

**TABLE 1:** Data layers.

Used data layer	Units	Spatial resolution	Temporal resolution	Source
EVI	—	250 m	16‐day (dynamic)	Didan (2021)
LST_Day_1 km (day land surface temperature)	K	1000 m	8‐day (dynamic)	Wan et al. (2021)
Precipitation	mm/5 days	5566 m	5‐day (dynamic)	Funk et al. (2015)
Extractable phosphor at 20–50 cm depth	ppm	30 m	Recorded between 2001 and 2017 (static)	Hengl et al. (2021)
Extractable nitrogen at 20–50 cm depth	g/kg	30 m	Recorded between 2001 and 2017 (static)	Hengl et al. (2021)
Elevation	m	30 m	Recorded in 2000 (static)	NASA JPL (2020)
Permanent waterbodies	m	—	2012 (static)	Elephants Alive

*Note:* The layer of the permanent waterbodies consists of vector based data, which has no spatial resolution.

The data on the nutrient contents were available for a soil depth of 0–20 and 20–50 cm, with a very high correlation (> 0.9) between the two depth layers (Appendix 1). As the savanna is a more deeply rooted biome compared to grasslands and other biomes (Jackson et al. 1996), only the nutrient data from 20 to 50 cm depth were used.

### 2.3 Data Preparation

The data analyses were conducted using R version 4.2.1 (R Core Team 2021) and ArcGIS Pro 3.0.0 (Esri 2022). The complete R script is available in Appendix S1.

#### 2.3.1 Revisitation Analyses

To identify revisitation sites, the R package ‘recurse’ was used. This package was developed to identify revisited sites, by calculating how often a circle of a pre‐specified size is revisited. This circle is then moved along the tracking data points (Bracis et al. 2018) for each individual elephant. To find the optimal circle size for the recursion analyses, we compared circles with a 100, 200, 250, 300, 400 and 500 m radius with the recurse package. The variance in the number of revisitations differed by circle size. When a circle is too small, the variance of the number of revisitations is low, because all sites are only revisited a few times. A too large circle also has a low variance, because then all sites are revisited equally often. It is therefore best to use the circle size where the variance peaks (Bracis 2019). For most elephants in this study the variance peaked at a circle size between 200 and 300 m, so a circle size of 250 m radius was selected for further analyses.

In order to revisit a site, the elephants must have left the site before re‐entering it again. To prevent labelling brief excursions outside the site as a separate revisitation, the elephants had to leave the site for a minimum of 12 h before being considered a separate revisitation. Afterwards, the locations of the revisitation sites were extracted, together with the number of revisitations of each revisitation site and the time of entry of each revisitation.

#### 2.3.2 Waterbodies

All spatial layers on waterbodies were converted into one shapefile containing all waterbodies with a buffer of 500 m. Since waterbodies are known to be frequently revisited for drinking, revisitation sites within the 500 m buffer were filtered out of the dataset.

#### 2.3.3 Joining Environmental Data to the Revisitation Data

In GEE, the environmental variables were selected, and the slope was calculated in degrees using the elevation. The slope was transformed using a log scale, hereafter referred to as ‘slope’, as the data had a skewed distribution with many low‐slope values and fewer high‐slope values. For dynamic data (e.g., precipitation, EVI), the last record before the time of entry of each revisitation was used. Static data (e.g., the nutrient contents and slope) were extracted once for each revisitation site. In order to assess the effect of the distance to waterbodies on the number of revisitations, the Euclidean distance from each revisitation site to the nearest waterbody was calculated. As available area increases quadratically with distance from water, the distance to water was square‐root transformed to correct for this undesired effect, hereafter referred to as ‘distance to water’.

#### 2.3.4 Seasonality

The number of revisitations was calculated separately for the wet and dry seasons. As the timing and duration of the seasons can vary strongly between years, the biologically relevant dry and wet seasons were determined using code sourced from Ecoscope (Wildlife Dynamics, n.d.) with NDVI as an indicator for the seasons. The NDVI values were extracted and standardised for the entire period of the elephant tracking data. Per year, the two cut points in NDVI values for the start and end of the wet and dry seasons were calculated.

### 2.4 Statistical Analyses

The elephant tracking data contained individuals that differed in the length of their tracking period, causing lower revisitation numbers for elephants with for example, only 1 year of data, even though they might revisit some sites frequently. Therefore, for each elephant the number of revisitations per site were rescaled to the revisitations per site in each of the seasons, which we refer to as the revisitation rate for that site. Since the distribution of revisitation rates was skewed, with many low values and fewer high values, we assessed the best‐fitting distribution by comparing the normal, log‐normal, Weibull and gamma distributions, using the fitdistr package (Delignette‐Muller and Dutang 2015). The log‐normal distribution suited the data best, so the logarithm of the revisitation rate was used as the response variable in all models.

The correlations between the different variables were assessed. As nitrogen and phosphorus were highly correlated (> 0.5), and selection of phosphorus‐rich forage is more important for elephants (Pretorius et al. 2012), nitrogen was left out of the analyses. So, the results obtained from the phosphorus analysis could maybe also be partly explained by a nitrogen effect. No strong correlations were found between any of the other variables.

#### 2.4.1 Environmental Conditions of Available and Revisited Sites

To analyse the non‐randomness in the locations of the revisited sites, we generated 100,000 random points using complete spatial randomness in the area denoted as the home range of the elephants. Assessing the non‐randomness by generating random points within the home range has proven effective in previous studies (Sitompul et al. 2013). We defined the home range using an adaptive local convex hull (a‐LoCoH) method, as this method accounts for spatial structure and barriers within the movement data (Getz et al. 2007). The radius was set to the maximum observed distance between points, ensuring that local hulls were constructed using all elephant data. An isopleth level of 1 was used to encompass 100% of the estimated home range. The 500 m buffered water area was excluded from the home range area before generating the random points. These random points were then also given randomly selected timestamps of entrance time from the revisitation data, as this was necessary to obtain the dynamic environmental values for these random points. Afterwards, the environmental values for the random points were obtained the same way as those for the revisitation sites. To assess whether there was a difference between the available sites (the random data) and the revisitation sites (the elephant data), the occurrence density of available and selected revisitation sites over the gradient of each variable were compared. To test for a difference between the occurrence density of the random sites and the elephant revisitation sites, a Kolmogorov test was used. The Kolmogorov test was applied in a similar way in other ecological studies, such as Drees and Shea (2024) and Somveille and Ellis‐Soto (2022). This test assesses the maximum distance (*D*) between the cumulative frequency distributions of two groups, taking into account the sample sizes of those groups. However, due to the extremely high sample size of the elephant and the random data, the *p* values of the Kolmogorov test will be highly significant even if the distance between the cumulative frequency distributions is very small, and thus has no biological relevance. To correct for this, and in order to get equal sample sizes of the elephant and random data, the Kolmogorov test was performed 10,000 times on random sub‐samples of the data, consisting of 100 elephant and 100 random datapoints.

#### 2.4.2 Environmental Predictors of Revisitation Rates

The relation between the separate environmental variables and the (log)revisitation rate was analysed, using linear models with environmental factors as explanatory variables. Since there is less data at the extremes of the environmental variables, for example, very high or very low phosphorus concentration, the statistical analyses would be dominated by the intermediate data. To account for this imbalance in the data, per environmental variable the data was split into a 1000 bins with an equal interval over the range of the variable, and the mean revisitation rate per bin was used in all regression analyses. Binning data has proven effective in previous studies (Kalkuhl and Wenz 2020).

#### 2.4.3 Factors Influencing Revisitation Rate

To assess which environmental variables influence the foraging site revisitation rate most, a linear mixed effects model including all environmental variables was fitted, with the individual elephants included as random effects. Prior to analyses, all predictor variables were scaled to zero mean and unit variance, allowing for comparing the relative importance of the coefficients. We performed model selection, using backward selection, based on the AIC values. This backward selection method is frequently used in ecological studies (e.g., Walker et al. 2014). Since we hypothesised that the relation between the revisitation rate and the environmental variables differs between the sexes and seasons (Figure 1), we fitted models separately for the sexes and seasons.

To account for the aforementioned imbalance in the data, that is, the high amount of data at intermediate values and low amount of data at the extremes, the models were fitted on resampled data that was obtained by stratified sampling over a gradient of the environmental variables. The *R*‐squared of the models were obtained by fitting the models 10,000 times on different resamples and taking the average. The marginal *R*‐squared ($R_{m}^{2}$) considers only the variance of the fixed effects, and the conditional *R*‐squared ($R_{c}^{2}$) considers both the variance of the fixed and the random effects, using the formulation of *R* ^2^ for mixed models as described by Nakagawa and Schielzeth (2013).

For both sexes and seasons, the variables included in the full model were distance to water, EVI, phosphorus, temperature and slope. For all these variables, a quadratic form was included. In addition to the fixed effects, the elephant ID was included as a random effect, allowing for random intercepts. Since the models that fall within 2 ΔAIC from the model with the best AIC are considered to be equally good (Burnham and Anderson 2002), the simplest/most parsimonious model of these models within 2 ΔAIC was chosen to be the best model. After model selection on the models without interactions, interactions between environmental variables were added to improve the models. We only added interactions that ecologically made sense because of computational restrictions and to prevent data mining. Only interactions between variables that showed an interaction in interaction plots (Appendix 5) were added to keep the models simple. Afterwards, model selection was performed again.

## 3 Results

### 3.1 Environmental Conditions of Available and Revisited Sites

**FIGURE 3:**
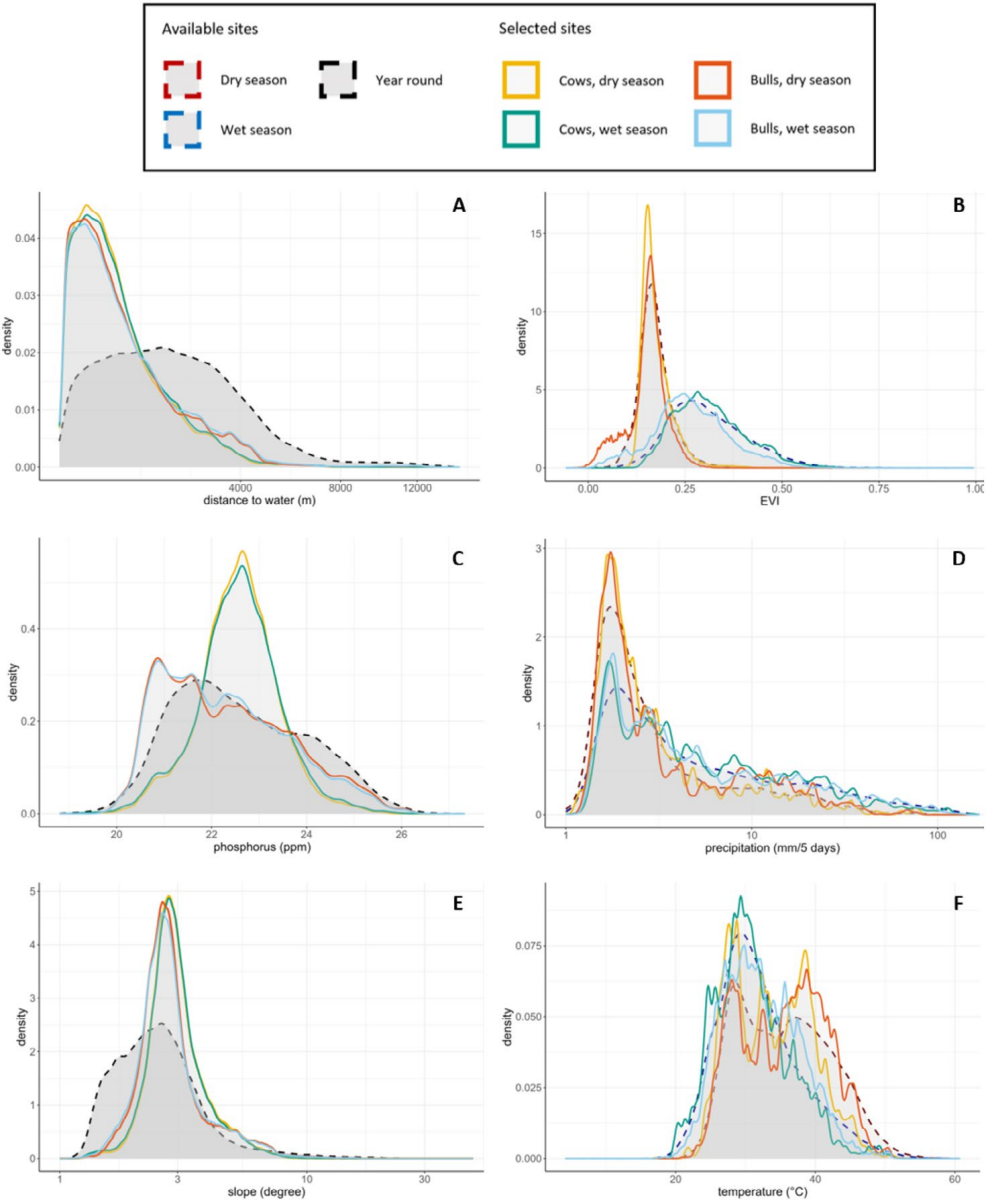
The occurrence density of sites, of all available sites (dotted lines) and revisitation sites (solid lines) over (A) distance to water, (B) EVI, (C) phosphorus, (D) precipitation, (E) slope and (F) temperature. For the dynamic variables, the available sites differ between the seasons, which means that there are two lines (red and blue) for the available sites. However, since the static variables are year round the same, these only have one line (black) for the available sites. Note that the distance to water (A) starts at a distance of 500 m, as sites closer to water were excluded from the analyses.

The occurrence density of the revisitation sites, selected by the elephants, was higher close to water than the density of the available sites (Figure 3A). The elephants thus selected revisitation sites closer to water (two‐sample Kolmogorov–Smirnov test, *D* = 0.427 ± 0.059, *p* = 4.8e‐05 ± 0.001; mean ± SD since the tests were performed 10,000 times on bootstraps of the data). Only the test results of the cows in the dry season are shown here; the test results for the bulls and wet season were similar to those of the cows in the dry season (Appendix 2).

For phosphorus, bulls occupied similar sites as there were available in both wet and dry season (two‐sample Kolmogorov–Smirnov test, *D* = 0.160 ± 0.049, *p* = 0.274 ± 0.271, and *D* = 0.162 ± 0.050, *p* = 0.262 ± 0.266, for the dry and wet season respectively). However, cows occupied more sites with an intermediate phosphorus concentration, while the available sites had a slightly higher occurrence density on the more extreme phosphorus concentrations (two‐sample Kolmogorov–Smirnov test, *D* = 0.236 ± 0.042, *p* = 0.024 ± 0.043, *D* = 0.227 ± 0.042, *p* = 0. 035 ± 0.059, for the dry and wet season respectively).

In terms of topographic slope, elephants selected revisitation sites at more intermediate slopes, while lower slopes were available for both seasons and sexes (two‐sample Kolmogorov–Smirnov test, *D* = 0.358 ± 0.057, *p* = 5.3e‐04 ± 0.003). When considering EVI, precipitation and temperature, the occurrence densities of the revisitation sites and the available sites were similar over the environmental gradients (two‐sample Kolmogorov–Smirnov test, *D* = 0.142 ± 0.036, *p* = 0.346 ± 0.248, *D* = 0.122 ± 0.036, *p* = 0.502 ± 0.290 and *D* = 0.176 ± 0.049, *p* = 0.192 ± 0.223 respectively).

### 3.2 Environmental Predictors of Revisitation Rates

**FIGURE 4:**
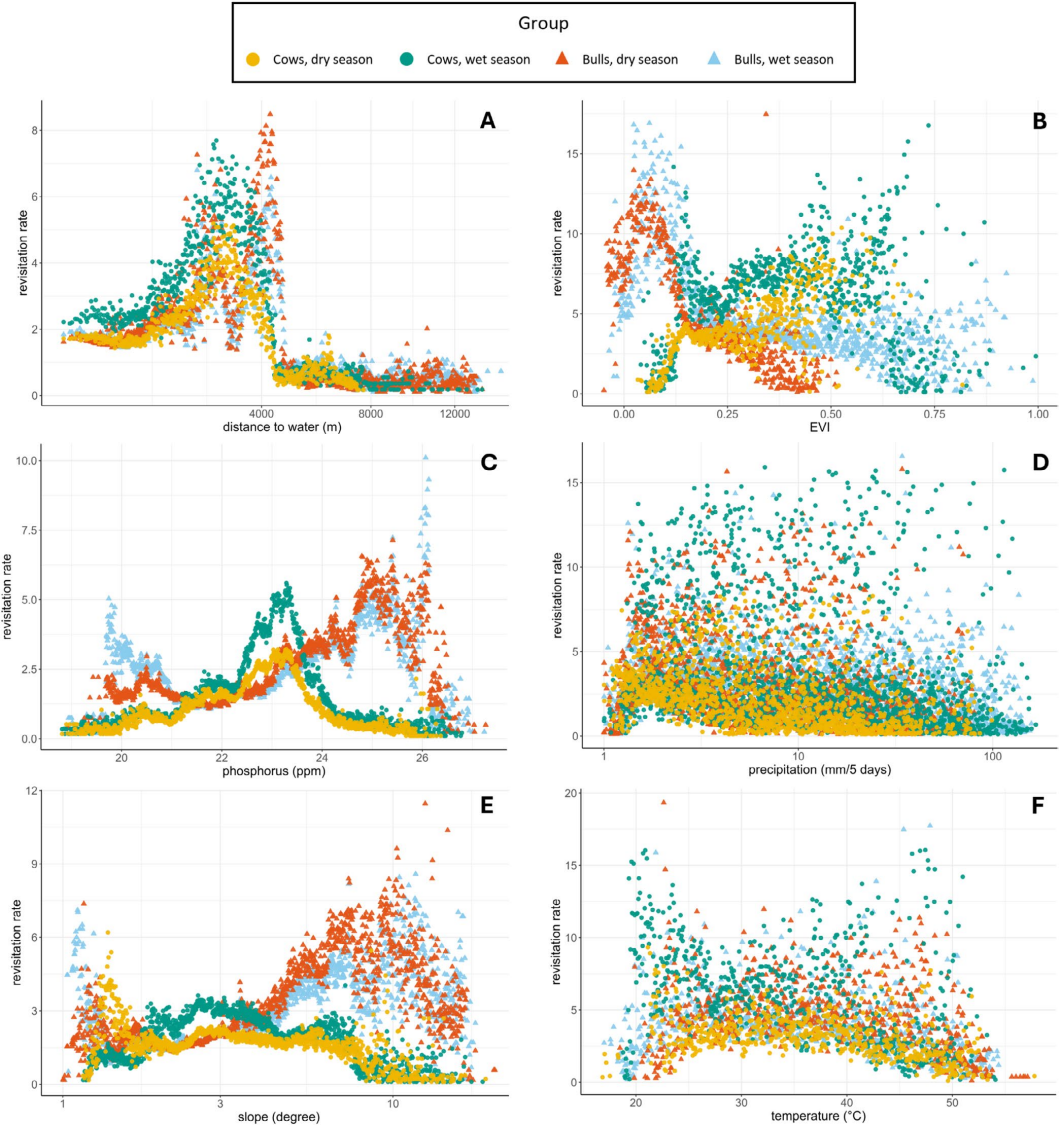
Relations between the mean revisitation rate per bin and the environmental variable; (A) distance to water, (B) EVI, (C) phosphorus, (D) precipitation, (E) slope and (F) temperature.

The mean revisitation rate increased at first with the distance to water (Figure 4A), before decreasing strongly, as was hypothesised (Table 2). For phosphorus, the revisitation rates of the bulls increased over increasing phosphorus concentration, also in line with our expectation, but the cows had the highest revisitation rate at intermediate phosphorus concentrations. Contrary to what was expected, the revisitation rates of the bulls had a negative relation with EVI (Table 2).

**TABLE 2:** Outcome linear regression models on the relations between the mean revisitation rate and the environmental variable. Including the hypothesised relations, and the plotted model predictions. Here the red lines represent the dry season, the blue lines represent the wet season and the brown line represents both seasons. Additionally, the dotted lines represent the cows, and the solid lines represent the bulls.

		Cows, dry season	Cows, wet season	Bulls, dry season	Bulls, wet season	Expected	Model predictions
Distance to water	*X*	2.385	1.365	0.253	0.427	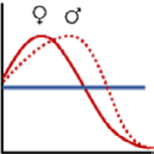	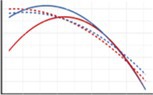
	*X* ^2^	−2.873	−2.182	−1.019	−1.088		
	*R* ^2^	0.681	0.708	0.529	0.591		
EVI	*X*	1.412	2.070	−0.253	−0.623	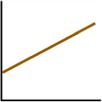	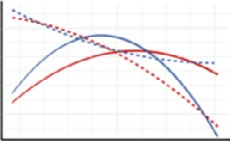
	*X* ^2^	−1.035	−2.182	−0.521	0.227		
	*R* ^2^	0.501	0.377	0.687	0.410		
Phosphorus	*X*	17.450	18.075	4.873	4.873	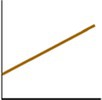	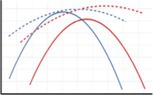
	*X* ^2^	−17.487	−18.126	−4.589	−4.701		
	*R* ^2^	0.736	0.774	0.315	0.165		
Precipitation	*X*	−0.229	0.978	0.728	0.693	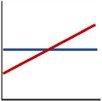	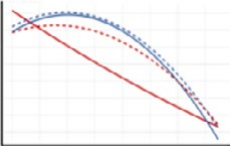
	*X* ^2^	−0.263	−1.431	−1.111	−1.036		
	*R* ^2^	0.278	0.295	0.218	0.296		
Slope	*X*	1.020	2.161	1.012	0.298	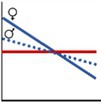	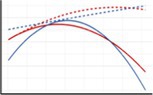
	*X* ^2^	−1.457	−2.593	−0.683	−0.021		
	*R* ^2^	0.533	0.702	0. 434	0.313		
Temperature	*X*	1.887	0.849	3.243	2.188	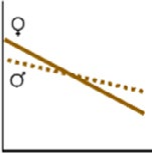	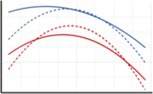
	*X* ^2^	−2.132	−1.142	−3.461	−2.426		
	*R* ^2^	0.352	0.200	0.435	0.397		

The data for precipitation (Figure 4D) did not show a clear relation with the revisitation rate. The linear regression models on precipitation also explain the data poorly; for both seasons and sexes, the *R*‐squared was below 0.25. As precipitation is not expected to be of direct importance to the elephants but mainly has its effect through vegetation (Rousvel et al. 2013), which is already represented in the data by EVI, precipitation was left out of further analyses.

**FIGURE 5:**
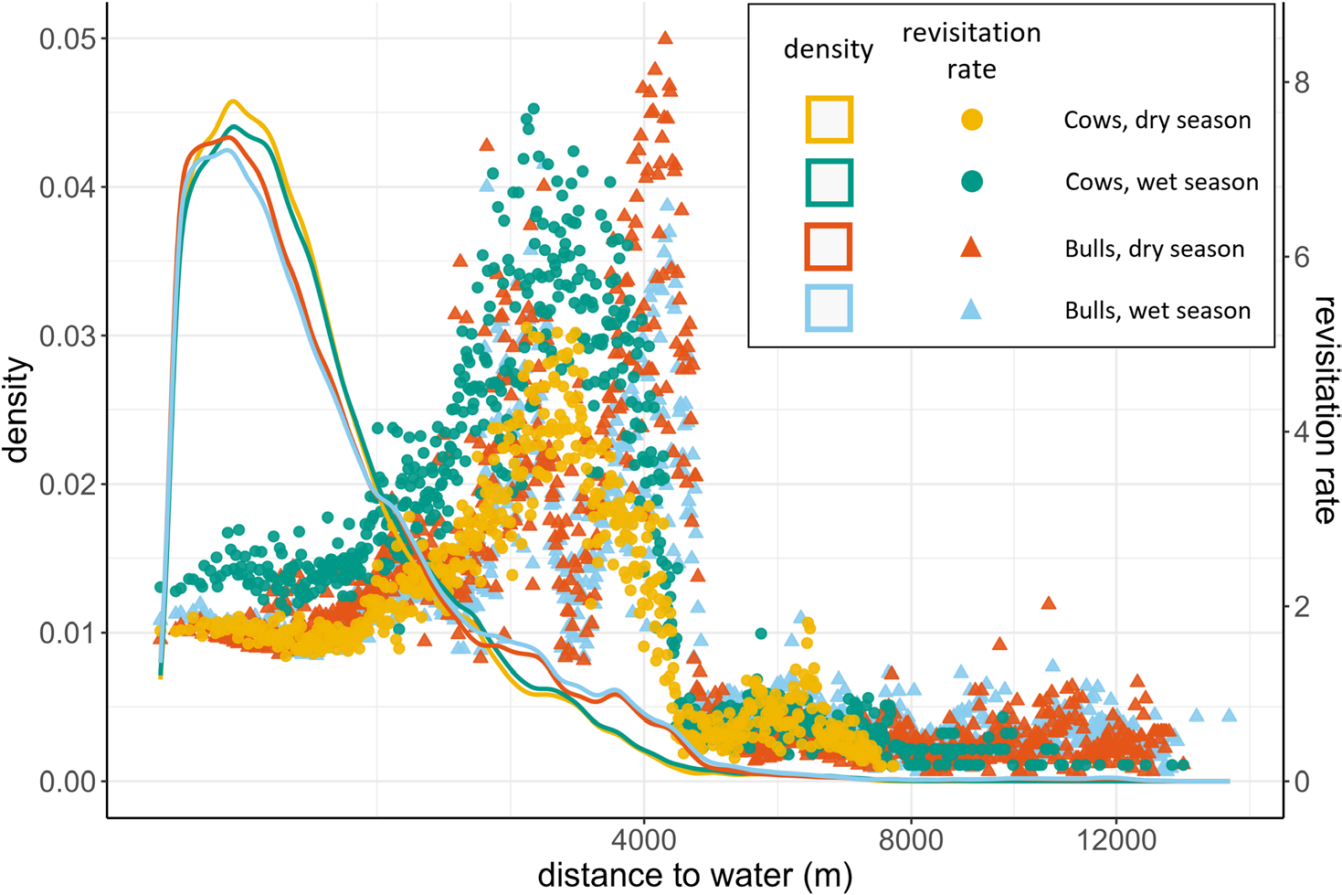
The occurrence density of selected revisitation sites, over distance to water, and the relation between the mean revisitation rate per bin and distance to water. The lines, belonging to the left *y*‐axis, represent the occurrence density of sites. The points, belonging to the right *y*‐axis, represent the revisitation rate per site.

For most environmental variables, the revisitation rate was highest where the occurrence density of the selected revisitation sites was low (Figure 5). The density of revisitation sites close to water is high, yet the revisitation rate for those sites is low. On the other hand, even though there were fewer revisitation sites at intermediate distances from water, these sites were revisited more often. For the patterns of other variables, see Appendix 3.

### 3.3 Factors Influencing Revisitation Rate

For both cows and bulls in both the dry and the wet season (Table 3), temperature is not important for selecting what sites to revisit. Phosphorus seems the main factor driving the revisitations, as the scaled coefficient of phosphorus is larger than those of the other variables, except for bulls in the dry season. Especially for cows, higher phosphorus concentrations appeared to be related to higher revisitation rates. Yet, the model also indicated a non‐linear relationship, as the squared phosphorus was retained in the model, suggesting a decrease in revisitation rate after a certain threshold of phosphorus concentration. Distance to water, EVI and slope are also important for the revisitation rate, though they are less important than phosphorus.

**TABLE 3:** Fitted models falling within 2 ΔAIC form the model with the lowest AIC, per group (cows/bulls, dry/wet). Per group, the red box highlights the most parsimonious model (the model with the lowest number of predictors, yet the smallest ΔAIC), which is chosen as the best model. For the environmental variables, the values represent the standardised regression coefficients. The *R*‐squared values of the most parsimonious models are the following: $R_{m}^{2}$ = 0.155 ± 0.014, $R_{c}^{2}$ = 0.480 ± 0.018 (cows, dry season), $R_{m}^{2}$ = 0.087 ± 0.011, $R_{c}^{2}$ = 0.576 ± 0.015 (cows, wet season), $R_{m}^{2}$ = 0.057 ± 0.010, $R_{c}^{2}$ = 0.421 ± 0.020 (bulls, dry season), $R_{m}^{2}$ = 0.059 ± 0.010, $R_{c}^{2}$ = 0.406 ± 0.023 (bulls, wet season).

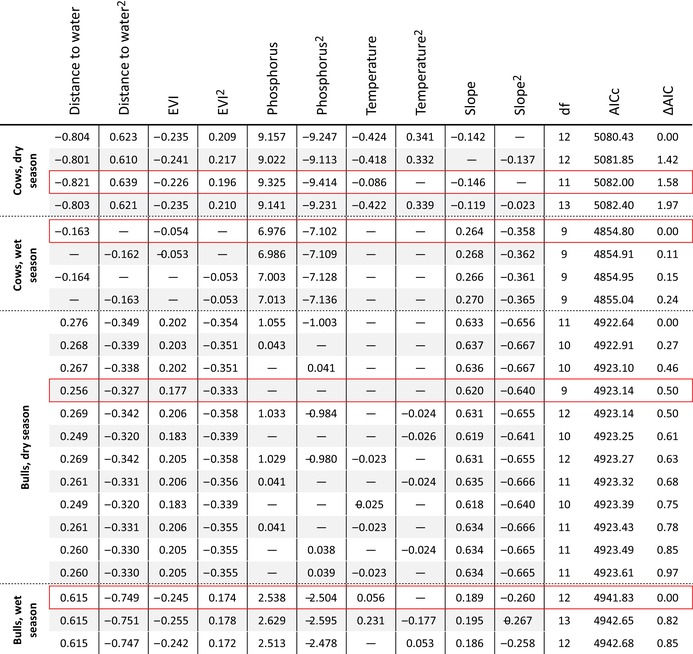

For the cows in the dry season (Appendix 4), the interaction between EVI and slope was included in the best model, meaning that the revisitation rate was higher at sites with high EVI values when these sites are also on low slopes. The interaction between distance to water and slope was also included in this model, as the cows revisited sites close to water more often when these were on the lowest slopes. For the cows in the wet season (Appendix 4), only the interaction between distance to water and slope turned out to improve the model, as it did for the bulls in the dry season (Appendix 4). For the bulls in the wet season, the interaction between the EVI and phosphorus improved the model, as the revisitation rate was higher at sites with low EVI values when the phosphorus concentration was higher. Additionally, the interaction between distance to water and phosphorus also improved the model for the bulls in the wet season, as they had higher revisitation rates at longer distances from water when the phosphorus concentration was higher (Appendix 4).

## 4 Discussion

This study aimed to gain further insight into the spatial patterns of site revisitations by elephants, by analysing the spatial non‐randomness of revisited sites in terms of environmental covariates, and by analysing how environmental covariates influence the revisitation rate, that is, the revisitations per site in each of the seasons. The results indicate that the environmental covariates related to the selection of the revisitation sites differ from the environmental covariates related to the revisitation rate. The majority of the revisitation sites are located close to water and at intermediate slopes; however, the revisitation rate is higher for sites at intermediate distances from water and higher phosphorus concentrations. The results also indicate that cows revisited sites with greener vegetation more, while bulls frequently revisit sites with less green vegetation and higher slopes. In addition, we found that phosphorus is the most important factor driving the patterns of site revisitations.

Similar to studies on habitat selection, the elephants were found to select for revisitation sites close to permanent waterbodies in the dry season (Harris et al. 2008). However, unlike previous studies, the elephants maintained the same spatial distribution with regard to water in the wet season. This might be due to familiarity, as elephants are known to show high fidelity to locations over long periods of time (Fishlock et al. 2016; Presotto et al. 2019), even when these locations are preferred neither for quality nor accessibility. Similar examples are known from other species as well. For example, bison keep revisiting the same areas, even when these are of poor quality and more profitable areas are readily available, as their familiarity with those sites gives them an advantage in dominance, foraging efficiently, escaping predators effectively, etc. (Merkle et al. 2015).

The elephants thus selected a lot of revisitation sites close to water, but when assessing the revisitation rate, the results show that, as expected, there is a quadratic relation between the distance to water and the revisitation rate. Since the elephant density is high close to water sources, and there is thus more competition in these areas, woody vegetation is browsed upon more at closer distances to water, the so‐called piosphere effect (De Beer et al. 2006). Therefore, it is likely more profitable to revisit sites at greater distance from water, as there will be more food available there. The revisitation rate starts declining after 4 km, which aligns with previous findings showing that, on average, elephants start moving towards water when they are 4.6 km away (Polansky et al. 2015).

In terms of topographic slope, the difference between the available sites and revisitation sites does not support our hypothesis that elephants would select revisitation sites with lower slopes. Contrary to our expectations, the elephants' revisitation sites show a lower occurrence density on the lowest slopes (< 2). This also differs from the patterns of habitat selection, as previous studies on habitat selection found that both African and Sumatran elephants select for low slopes (Moßbrucker et al. 2016; Roever et al. 2012). This avoidance of the lowest slopes might be an artefact of the data, as there is comparatively less tracking data and collared elephant occurrences where the slopes are the lowest, as depicted within the Greater Kruger National Park. The lowest slopes were mainly found within the north‐eastern regions of the Kruger National Park, while the highest density of elephant tracking data, where elephants have been tracked for the longest, is to the west of the Kruger National Park. In addition, Asian elephants prefer slopes between 0° and 10° (Wilson et al. 2021), which is also the range that the elephants of this study inhabit, and the range that most of the Greater Kruger National Park falls within. Consistent with the hypothesised difference between cows and bulls, the cows have their highest revisitation rate at lower slopes than the bulls. The bulls do revisit the sites on the relatively steep slopes (> 10°) more often; this might be to avoid competition with family units (Evans and Harris 2012), as family units have difficulties navigating steep slopes due to the presence of young.

In contrast to what was expected, the elephants did not select for revisitation sites with higher‐than‐average phosphorus concentrations. The cows did show selection for intermediate phosphorus concentrations, while the bulls did not show selection for phosphorus concentrations. However, when assessing the revisitation rate, the elephants do revisit sites with higher phosphorus levels more often, which aligns with previous findings that elephants, throughout the year, maximise their intake of phosphorus (Greyling 2004; Pretorius et al. 2012). However, as phosphorus and nitrogen concentrations were positively correlated, these findings should be interpreted with care as part of the phosphorus effects could also be explained by nitrogen.

Tsalyuk et al. (2019) state that elephants choose patches with higher‐than‐average annual productivity and grass biomass. Consistent with previous studies showing that elephants consistently select greener than average vegetation throughout the year (Loarie et al. 2009), the cows had a higher revisitation rate at sites with relatively high EVI values, despite there being no difference between the EVI values of the selected revisitation sites and the available sites. Unlike what was expected, bulls revisited sites that had lower EVI values more often. It is known that there is spatial segregation between sexes, and bulls select for lower quality forage but more volume (Greyling 2004; Shannon et al. 2006). Since bulls do not experience the same social and energetic constraints as cows, they can feed for longer, having the chance to utilise and digest, due to their longer gut transit times, the more abundant but lower quality forage (Greyling 2004; Shannon et al. 2006).

Our findings reveal that phosphorus is the main environmental variable influencing the revisitation rate, which supports the hypothesis that nutrients are the most important factor for determining revisitations. In their study, Pretorius et al. (2012), who looked at the same area, found that elephants maximised the intake of phosphorus throughout the year, possibly due to the deficiency of this nutrient in the area. In addition, elephants living in areas with higher nutrient concentrations, including phosphorus, have been shown to have a smaller home range (Sach et al. 2020). Despite this, for the bulls in the dry season, phosphorus was not present in the best model; this might be because of their need to satisfy larger energy requirements and their capability to forage less selectively (Shannon et al. 2006).

The north‐western region of the Greater Kruger National Park, characterised by Mopane‐dominated woodlands on granite with patches of bushwillow and acacia species, has very low phosphorus concentrations. Similarly, the north‐eastern region, dominated by open savanna grasslands with stunted mopane, also exhibits low phosphorus levels. In contrast, regions in the south, which include mixed thornveld, marula woodlands, and thorn thickets, have relatively high phosphorus concentrations. However, phosphorus concentrations also differ on a smaller scale. Elephants are known to forage more on vegetation growing on termite mounds (Holdo and McDowell 2004), which adds to the evidence suggesting that nutrients are the most important factor for frequent revisitations to a site. Termite mounds create ‘islands of fertility’ (Levick et al. 2010). Plants on termite mounds have higher levels of calcium, magnesium, potassium and phosphorus and are known to be more intensely fed upon by elephants (Holdo and McDowell 2004). Additionally, certain plant species accumulate more phosphorus than others. Pretorius et al. (2011) found that elephants select patches with higher nutrient content in leaves from mopane trees, and maximise phosphorus intake on an annual basis (Pretorius et al. 2012). More research on field data is needed to see if elephants indeed return to sites with the most nutritious plants.

In addition to the nutrient intake through vegetation, elephants are also known to exhibit geophagy, or soil consumption. It is thought that this behaviour aids elephants in meeting their mineral requirements (Sach et al. 2019), especially in the dry season. Holdø et al. (2002) found that nutrient levels in browse species are lower in the dry season compared to the wet season, and suggest that they are insufficient to meet requirements.

Distance to water, EVI and slope also proved influential to the revisitation rate. Yet, distance to water is less important than phosphorus in determining how often sites are revisited; this might be because water mainly determines what areas elephants select for, but not which ones they return to. In addition, EVI might be more influential to the time the animals stay in a foraging patch (Engelen 2023) than to where they frequently return to forage, as elephants have more tortuous movement in habitat types that have favourable resources such as forage (Duffy et al. 2011).

The results showed that temperature was not important for determining the revisitation rate, even though thermoregulation is essential for elephants, and large herbivores tend to avoid open areas during hotter periods (Valeix et al. 2008). However, as Mashintonio et al. (2014) stated in their research ‘a preference for sheltering under trees does not necessarily imply a fondness for continuous forest’. This is also one of the aspects that can be improved in any future analyses, as selection of different variables occurs at different scales. In addition, the selection of a variable is also dependent on the availability of that variable at a broader scale, that is, the functional response in habitat selection. Moreover, elephants prefer sites that have high neighbourhood quality (De Knegt et al. 2011; Mashintonio et al. 2014). Both the habitat selection based on environmental qualities at multiple scales and the preference for sites with a high neighbourhood quality were not considered in this study, which might explain the low *R*‐squared of the models. More research is needed to incorporate selection on different scales in the analyses and to assess what other factors drive site revisitations in the field.

Another reason why the models did not explain the data very well might be the large differences between individual elephants. This is supported by the substantial variation accounted for by the random effects, which include elephant identity. Additionally, Fishlock et al. (2016) showed that elephant fidelity to particular sites arises through social learning and results in traditional behaviour over generations, which can weaken relationships between resource quality and site preferences. This might also increase the individual differences, since the weakened relationships between resource quality and site preferences can lead to different families passing along these preferences to their offspring, thus creating fidelity towards different sites.

By comparing the selected revisitation sites to the available sites, we showed that elephants select for revisitation sites close to water and sites at intermediate slopes. The cows also select revisitation sites with intermediate phosphorus concentrations. However, the elephants revisit sites more frequently when these are at intermediate distances from water and higher phosphorus concentrations. Cows revisit sites with greener vegetation more frequently, while bulls more often revisit sites with less green vegetation and higher slopes. The results show that phosphorus is the main environmental variable influencing the rate of revisitation. Our findings indicate that the relation between the environmental variables and the selection of the revisitation sites differs from the relation between these environmental variables and the revisitation rate, as the most revisited sites are not necessarily on the environmental variable values elephants select revisitation sites for. Additionally, our findings indicate that the relation between the environmental variables and the revisitation patterns varies from the relation between environmental variables and habitat selection found in other studies. Looking at revisitations thus gives more insight into the foraging behaviour of elephants and improves our understanding of spatial patterns of habitat use by elephants.

## Author Contributions

**Suzanne Antoinette Jacob:** conceptualization (equal), formal analysis (lead), writing – original draft (lead), writing – review and editing (equal). **Willem Frederik De Boer:** conceptualization (equal), formal analysis (supporting), writing – original draft (supporting), writing – review and editing (equal). **Henrik Johan De Knegt:** conceptualization (equal), formal analysis (supporting), writing – original draft (supporting), writing – review and editing (equal). **Cassander Cassijn Engelen:** conceptualization (equal), formal analysis (supporting), writing – original draft (supporting), writing – review and editing (equal). **Michelle Henley:** conceptualization (equal), formal analysis (supporting), writing – original draft (supporting), writing – review and editing (equal).

## Conflicts of Interest

The authors declare no conflicts of interest.

## Supporting information


Appendix S1

## Figures and Tables

**FIGURE A1:**
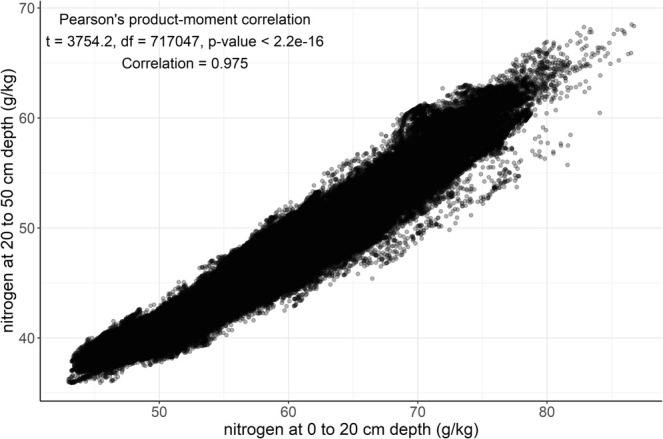
Correlation between the nitrogen concentrations at the two depths.

**FIGURE A2:**
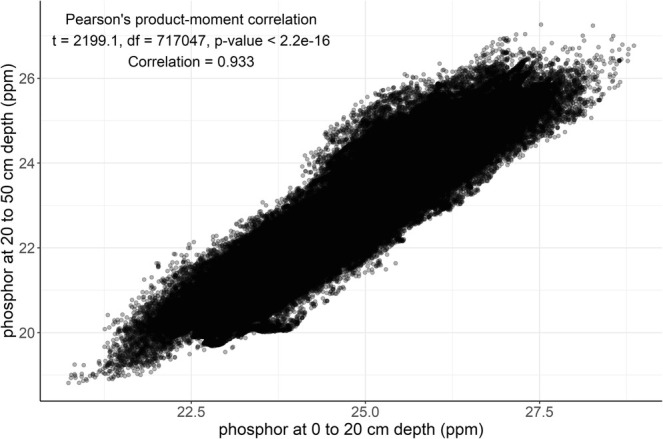
Correlation between the phosphorus concentrations at the two depths.

**FIGURE A3:**
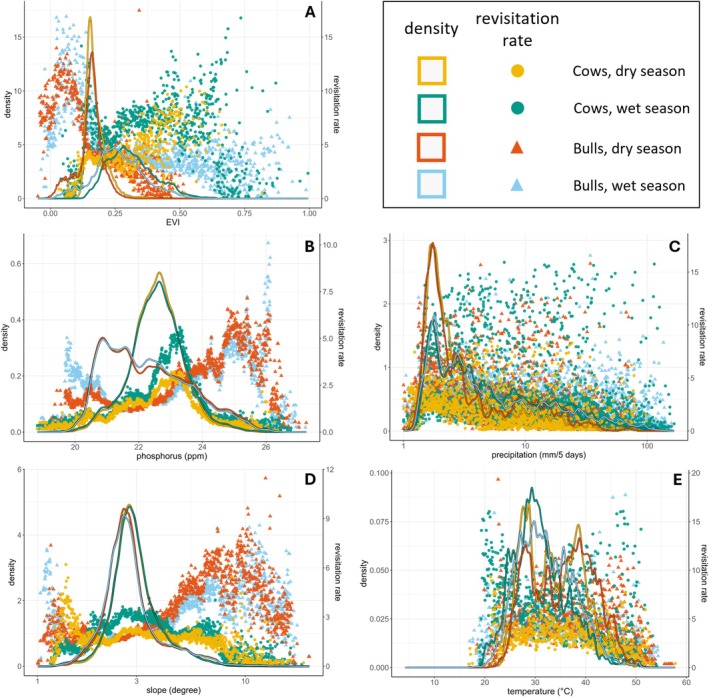
Density, which is the occurrence density of selected sites, over the environmental variables, and the relation between the mean revisitation rate per bin and that environmental variable. The lines, belonging to the left *y*‐axis, represent the occurrence density of sites. The points, belonging to the right *y*‐axis, represent the revisitation rate per site.

**FIGURE A4:**
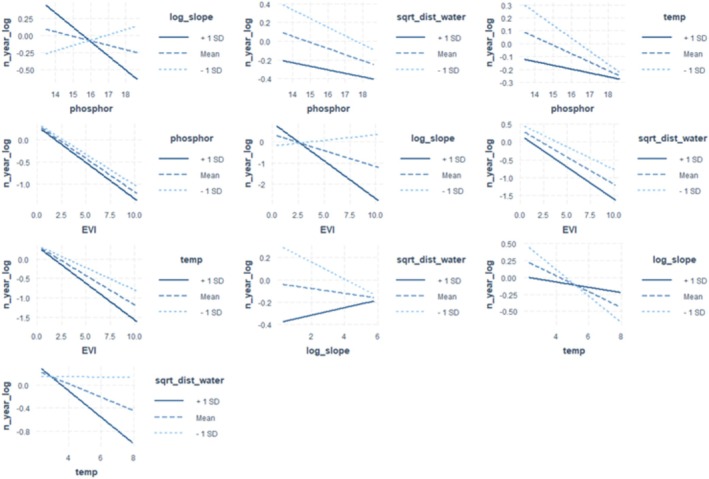
Interactions between the variables of the selected best model without interaction, for the cows in the dry season.

**FIGURE A5:**
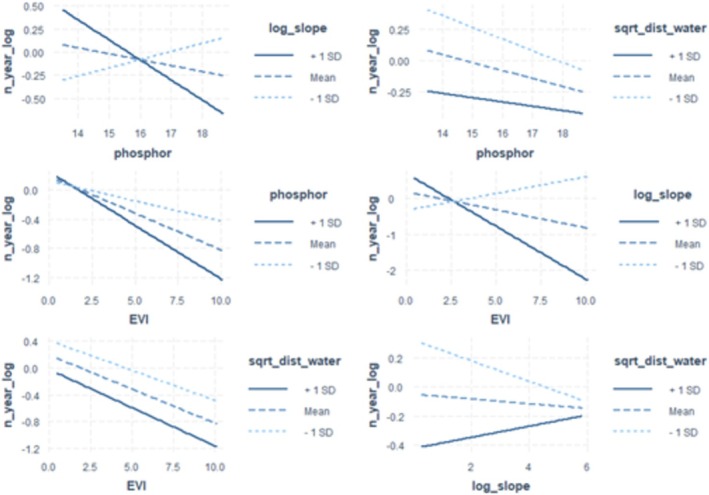
Interactions between the variables of the selected best model without interaction, for the cows in the wet season.

**FIGURE A6:**
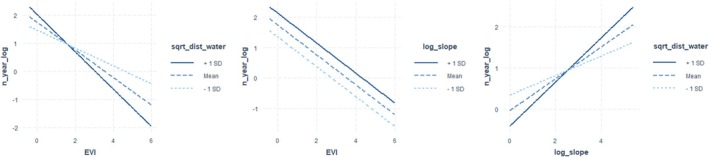
Interactions between the variables of the selected best model without interaction, for the bulls in the dry season.

**FIGURE A7:**
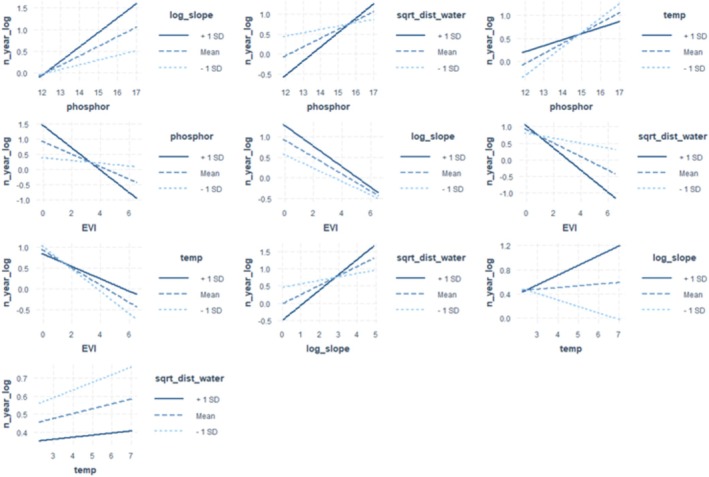
Interactions between the variables of the selected best model without interaction, for the bulls in the wet season.

## Data Availability

We refrain from publicly sharing any data regarding the animal's locations (GPS tracking data and home range data) and their revisitation sites due to security restrictions involving collared elephants. The R code that was used, and the metadata, are uploaded as Appendix S1.

## Appendix 1

The correlation between nutrient levels at 0–20 cm depth and 20–50 cm depth for nitrogen (Figure A1) and phosphorus (Figure A2).

## Appendix 2

Below, the output of the Kolmogorov tests for both sexes and seasons is listed (Table A1). The results are given for the Kolmogorov test on the whole data set, and for the sub‐sampled data.

**TABLE A1:** Difference between the occurrence density of the available sites and the elephant revisitation sites, using the two‐sample Kolmogorov–Smirnov test.

			Cows, dry season	Cows, wet season	Bulls, dry season	Bulls, wet season
Distance to water	All data	*D*	0.395	0.379	0.350	0.334
		*p*	< 2.2e‐16	< 2.2e‐16	< 2.2e‐16	< 2.2e‐16
	Sub‐sample	*D*	0.427 ± 0.059	0.414 ± 0.059	0.390 ± 0.059	0.372 ± 0.060
		*p*	4.8e‐05 ± 0.001	5.6e‐05 ± 0.001	2.2e‐04 ± 0.003	4.6e‐04 ± 0.005
EVI	All data	*D*	0.087	0.044	0.125	0.156
		*p*	< 2.2e‐16	< 2.2e‐16	< 2.2e‐16	< 2.2e‐16
	Sub‐sample	*D*	0.142 ± 0.036	0.125 ± 0.040	0.192 ± 0.050	0.216 ± 0.056
		*p*	0.346 ± 0.248	0. 487 ± 0.300	0.130 ± 0.173	0.084 ± 0.151
Phosphorus	All data	*D*	0.176	0.166	0.099	0.096
		*p*	< 2.2e‐16	< 2.2e‐16	< 2.2e‐16	< 2.2e‐16
	Sub‐sample	*D*	0.236 ± 0.042	0.227 ± 0.042	0.160 ± 0.049	0.162 ± 0.050
		*p*	0.024 ± 0.043	0. 035 ± 0.059	0.274 ± 0.271	0.262 ± 0.266
Precipitation	All data	*D*	0.043	0.055	0.063	0.032
		*p*	< 2.2e‐16	< 2.2e‐16	< 2.2e‐16	< 2.2e‐16
	Sub‐sample	*D*	0.122 ± 0.036	0.130 ± 0.041	0.131 ± 0.039	0.121 ± 0.038
		*p*	0.502 ± 0.290	0.455 ± 0.299	0.438 ± 0.287	0.513 ± 0.296
Slope	All data	*D*	0.326	0.317	0.247	0.228
		*p*	< 2.2e‐16	< 2.2e‐16	< 2.2e‐16	< 2.2e‐16
	Sub‐sample	*D*	0.358 ± 0.057	0.350 ± 0.057	0.279 ± 0.056	0.263 ± 0.056
		*p*	5.3e‐04 ± 0.003	7.7e‐04 ± 0.005	0.011 ± 0.034	0.021 ± 0.060
Temperature	All data	*D*	0.132	0.065	0.045	0.053
		*p*	< 2.2e‐16	< 2.2e‐16	< 2.2e‐16	< 2.2e‐16
	Sub‐sample	*D*	0.176 ± 0.049	0.138 ± 0.045	0.128 ± 0.039	0.129 ± 0.042
		*p*	0.192 ± 0.223	0.403 ± 0.303	0.462 ± 0.289	0.460 ± 0.300

## Appendix 3

The highest revisitation rates for EVI, phosphorus and slope are at different values of these environmental variables than the highest density of selected sites (Figure A3). For the cows, the sites they revisit most often have higher EVI values and slightly higher phosphorus concentrations than the sites they select most. The bulls have an even clearer difference in phosphorus concentration, and also revisit sites most at lower EVI values and higher slopes than the sites they select for.

## Appendix 4

Model tables of models with interactions. For the cows in the dry season (Table A2), the cows in the wet season (Table A3), the bulls in the dry season (Table A4) and the bulls in the wet season (Table A5).

**TABLE A2:** Fitted models falling within 2 ΔAIC form the model with the lowest AIC. The underlined numbers belong to the most parsimonious model (the model with the lowest number of predictors, yet the smallest ΔAIC), which is chosen as the best model. For the environmental variables, the values represent the standardised regression coefficients. The *R*‐squared values of the most parsimonious models; $R_{m}^{2}$ = 0.175 ± 0.014, $R_{c}^{2}$ = 0.493 ± 0.018.

Distance to water	Distance to water^2^	EVI	EVI^2^	Phosphorus	Phosphorus^2^	Slope	Temperature	Slope × Distance to water	Slope × EVI	Slope × Phosphorus	Slope × Temperature	df	AICc	ΔAIC
−0.885	0.392	0.205	—	7.455	−7.632	−0.148	−0.101	0.137	−0.109	—	—	12	4954.92	0.00
−0.883	0.387	0.203	—	7.448	−7.625	−0.279	−0.157	0.138	−0.108	—	0.026	13	4955.37	0.45
−0.882	0.392	0.112	0.081	7.439	−7.617	−0.164	−0.103	0.136	−0.101	—	—	13	4955.90	0.98
−0.880	0.387	0.098	0.091	7.429	−7.608	−0.309	−0.165	0.137	−0.099	—	0.028	14	4956.09	1.17
−0.887	0.388	0.202	—	7.563	−7.766	−0.373	−0.101	0.140	−0.109	0.014	—	13	4956.54	1.62

**TABLE A3:** Fitted models falling within 2 ΔAIC form the model with the lowest AIC. The underlined numbers belong to the most parsimonious model (the model with the lowest number of predictors, yet the smallest ΔAIC), which is chosen as the best model. For the environmental variables, the values represent the standardised regression coefficients. The *R*‐squared values of the most parsimonious models; $R_{m}^{2}$ = 0.088 ± 0.010, $R_{c}^{2}$ = 0.587 ± 0.015.

Distance to water	EVI	Phosphorus	Phosphorus^2^	Slope	Slope^2^	EVI × Phosphorus	EVI × Slope	Slope × Distance to water	Slope × Phosphorus	df	AICc	ΔAIC
−0.297	−0.065	7.295	−7.405	0.159	−0.479	—	—	0.081	—	10	4940.41	0.00
−0.296	−0.373	7.349	−7.388	0.161	−0.479	−0.028	—	0.081	—	11	4940.67	0.26
−0.304	−0.065	7.465	−7.465	−0.140	−0.507	—	—	0.084	0.020	11	4941.69	1.28
−0.303	−0.353	7.497	−7.570	−0.105	−0.503	−0.026	—	0.083	0.018	12	4942.12	1.71

**TABLE A4:** Fitted models falling within 2 ΔAIC form the model with the lowest AIC. The underlined numbers belong to the most parsimonious model (the model with the lowest number of predictors, yet the smallest ΔAIC), which is chosen as the best model. For the environmental variables, the values represent the standardised regression coefficients. The *R*‐squared values of the most parsimonious models; $R_{m}^{2}$ = 0.059 ± 0.009, $R_{c}^{2}$ = 0.422 ± 0.020.

Distance to water	Distance to water^2^	EVI	EVI^2^	Slope	Slope^2^	Distance to water × EVI	Distance to water × Slope	df	AICc	ΔAIC
−0.025	−0.254	0.155	−0.340	0.201	−0.450	—	0.106	10	5076.21	0.00
−0.060	−0.257	0.114	−0.333	0.192	−0.449	0.016	0.108	11	5077.80	1.59

**TABLE A5:** Fitted models falling within 2 ΔAIC form the model with the lowest AIC. The underlined numbers belong to the most parsimonious model (the model with the lowest number of predictors, yet the smallest ΔAIC), which is chosen as the best model. For the environmental variables, the values represent the standardised regression coefficients. The *R*‐squared values of the most parsimonious models; $R_{m}^{2}$ = 0.068 ± 0.010, $R_{c}^{2}$ = 0.425 ± 0.022.

Distance to water	Distance to water^2^	EVI	EVI^2^	Phosphorus	Phosphorus^2^	Slope	Slope^2^	Temperature	EVI × Phosphorus	EVI × Distance to water	Phosphorus × Distance to water	Slope × Temperature	df	AICc	ΔAIC
−1.008	−0.429	0.815	0.181	1.726	−1.812	0.169	−0.256	0.045	−0.071	—	0.094	—	14	5109.26	0.00
−1.096	−0.425	0.806	0.179	1.735	−1.823	0.093	−0.257	0.012	−0.070	—	0.094	0.017	15	5110.61	1.35
−1.125	−0.432	0.822	0.176	1.852	−1.954	—	−0.098	0.045	−0.071	—	0.097	—	13	5110.72	1.46
−1.065	−0.429	0.842	0.178	1.732	−1.815	0.168	−0.255	0.045	−0.071	−0.006	0.093	—	15	5111.22	1.96

## Appendix 5

After the first model selection, of the models without interactions, interaction plots were made to visually assess what interactions are present between the variables of the selected best model. These interactions were then added to the new models.

### Cows, Dry Season

Phosphorus and slope, slope and EVI, temperature and slope and slope and distance to water have the highest angle between the +1 SD and the −1 SD lines (Figure A4). These interactions were therefore included in the model.

### Cows, Wet Season

Phosphorus and slope, EVI and phosphorus, EVI and slope and slope and distance to water have the highest angle between the +1 SD and the −1 SD lines (Figure A5). These interactions were therefore included in the model.

### Bulls, Dry Season

EVI and distance to water and slope and distance to water have the highest angle between the +1 SD and the −1 SD lines (Figure A6). These interactions were therefore included in the model.

### Bulls, Wet Season

EVI and phosphorus, phosphorus and distance to water, slope and temperature and EVI and distance to water have the highest angle between the +1 SD and the −1 SD lines (Figure A7). These interactions were therefore included in the model.
