# Supplementary material for: Environmental Determinants of Foraging Site Revisitation by African Elephants ( Loxodonta africana )
Source: Ecol Evol. 2025 Jun 3;15(6):e71506. doi: 10.1002/ece3.71506 (PMC12134087; doi:10.1002/ece3.71506)
Supplement: Supplementary file 1 — Appendix S1 [file ECE3-15-e71506-s001.zip › R-Code-Data-Preparation.docx]

R Code - Data Preparation

Jacob, S.A.

2025-05-07

## Revisitation analyses

#### Find optimal circle size for the recursion analyses, using the variance

Load packages and data

*# ZnstaL L packages*

if(!require(pacman)){ install.packages(”pacman“) library(pacman)

*# Load packages*

pacman: :p_load(tidyverse, recurse, lubridate,

gridExtra, sf, data.table)

*# Load data*

data_raw <- read_csv(“Data/EA/EA_WAG_elelocs_20120101-20221021_clean.csv”)

Select the data for the recursion analyses

*# SeLect the necessary coLunns*

elephant.df_rec <- data_raw %>% subset(groupby_col == ”Proud“) %>%

dplyr: :select(extra easting, extra northing, fixtime, groupby_col) %>% as.data.frame()

Get recursion statistics


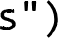


*# Get recurs ion statistics for each circ Le size* to *be tested*

elephant_rec1 <- getRecursions(elephant.df_rec, radius = 100, threshold = 12, timeunits = "hour rec_stats1 <- elephant_rec1$revisitStats

elephant_rec2 <- getRecursions(elephant.df_rec, radius = 200, threshold = 12, timeunits = "hour s..)

rec_stats2 <- elephant_rec2$revisitStats

elephant_rec2.5 <- getRecursions(elephant.df_rec, radius = 250, threshold = 12, timeunits = “hou rs“)

rec_stats2.5 <- elephant_rec2.5$revisitStats

| elephant_rec3 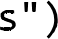 | <- getRecursions(elephant.df_rec, radius | = | 300, | threshold | = | 12, | timeunits | = | "hour |
| --- | --- | --- | --- | --- | --- | --- | --- | --- | --- |
| rec_stats3 <-  elephant_rec4 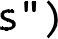 | elephant_rec3$revisitStats  <- getRecursions(elephant.df_rec, radius | = | 400, | threshold | = | 12, | timeunits | = | "hour |
| rec_stats4 <- | elephant_rec4$revisitStats |  |  |  |  |  |  |  |  |
| elephant_rec5 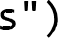 | <- getRecursions(elephant.df_rec, radius | = | 500, | threshold | = | 12, | timeunits | = | "hour |
| rec_stats5 <- | elephant_rec5$revisitStats |  |  |  |  |  |  |  |  |

*# Get the nunber of- rev!s i laibons per site*

meanl <- rec_stats1 %>% group_by(coordIdx) %>% summarise(maxvisit = max(visitIdx))

mean2 <- rec_stats2 %>%

group_by(coordIdx) g›g

summarise (maxvis1t = max(v1s1tIdx) )

mean2.5 ‹- rec_stats2.5 %>% group_by(coordIdx) %>% summarise(maxvisit = max(visitIdx))

mean3 <- rec_stats3 %>% group_by(coordIdx) %>% summarise(maxvisit = max(visitIdx))

mean4 <- rec_stats4 %>% group_by(coordIdx) %>% summarise(maxvisit = max(visitIdx))

mean5 <- rec_stats5 %>% group_by(coordIdx) %>% summarise(maxvisit = max(visitIdx))

#### Comparing the mean, max, and variance of the number of

revisitions

plotl ‹- ggplot() +

geom_point(aes(x = 100, y = mean(mean1$maxvisit))) + geom_point(aes(x = 200, y = mean(mean2$maxvisit))) + geom_point(aes(x = 250, y = mean(mean2.5$maxvisit))) + geom_point(aes(x = 300, y = mean(mean3$maxvisit))) + geom_point(aes(x = 400, y = mean(mean4$maxvisit))) + geom_point(aes(x = 500, y = mean(mean5$maxvisit))) + labs(title = "mean revisitations", x = "circle size (m)",

y = "mean revisitations")

plot2 <- ggplot() +

geom_point(aes(x = 100, y = max(mean1$maxvisit))) + geom_point(aes(x = 200, y = max(mean2$maxvisit))) + geom_point(aes(x = 250, y = max(mean2.5$maxvisit))) + geom_point(aes(x = 300, y = max(mean3$maxvisit))) + geom_point(aes(x = 400, y = max(mean4$maxvisit))) + geom_point(aes(x = 500, y = max(mean5$maxvisit))) + labs(title = "max revisitations", x = "circle size (m)",

y = "max revisitations")

plot3 ‹- ggplot() +

geom_point(aes(x = 100, y = var(log10(mean1$maxvisit)))) + geom_point(aes(x = 200, y = var(log10(mean2$maxvisit)))) + geom_point(aes(x = 250, y = var(log10(mean2.5$maxvisit)))) + geom_point(aes(x = 300, y = var(log10(mean3$maxvisit)))) + geom_point(aes(x = 400, y = var(log10(mean4$maxvisit)))) + geom_point(aes(x = 500, y = var(log10(mean5$maxvisit)))) + labs(title = "variance revisitations", x = "circle size (m)",

y = "variance revisitations")

grid.arrange(plotl, plot2, plot3, ncol=3)

Preform the revisitation analyses

*# Load data*

data_raw <- read_csv(“Data/EA/EA_WAG_elelocs_20120101-20221021_clean.csv”)

*# SeL ect coLunns*

recursions_df <- data_raw %>%

dplyr::select(extra easting, extra northing, fixtime, lon, lat) %>%

rename(utme = extra easting, utmn = extra northing, timestamp = fixtime, name = groupby_co l)%>%

as.data.frame()

Create function to automatically extract recursions

get_rec ‹- function(id){

*# Create dy*

recursions_df ‹- elephant_data %>% dplyr::select(utme, utmn, timestamp, name) %>% subset(name == id) %>%

as.data.frame()

*# Get recurs Rons*

recursions ‹- getRecursions(recursions_df, radius = 250, timeunits = “hours",

threshold = 12)

*# Get recurs hon stats*

recursion_stats <- as_tibble(recursions$revisitStats)

*# Return stats*

return(recursion_stats)

*# Zni mate enpty t!bbLe*

full_rec_stats <- tibble() %>% mutate(id = ”“)

Extract the recursions for each elephant

*# Run and attach the recurs ion statistics of- aLL eLephants*

for(i in unique(recursions_df$name)){

if(i==1){

full_rec_stats <- full_rec_stats %>% full_join(get_rec(i), by=c(“name”))

else(

print(paste0(“trying“, i)) try(full_rec_stats <- full_rec_stats %>%

bind_rows(get_rec(i)))

Write to csv

write_csv(“Data/all_data.csv“)

# Waterbodies

Filter out all revisitation sites with their center within 250m from

### waterbodies

*# Load data*

dat ‹- read_csv(“Data/all_data.csv“)

water <- st_read(“Data/Water_shp/All_water_500m_utm_updated.shp”)

*# Create data to safe coordinates for Later*

dat ‹- dat 8›%

mutate(to_join = paste(id, coordIdx, visitldx, timeSinceLastVisit, sep = "-"))

data_to_join <- dat %>% select(x, y, to_join)

*# Identify points ins ide and outs ide buffered water area*

points ‹- st_as_sf(dat, coords = c(”x“,“y”), crs=st_crs(water))

dat2 ‹- points %>%

mutate(inside = (lengths(st_intersects(points, water)) > 0)) %>% left_join(data_to_join, by = "to_join") %>%

as.data.frame() %>%

select(! c(to_join, geometry))

*# SpL it points k/ithin and outs ide buffered water area*

dat_near_water <- dat2 %>% filter(inside == "TRUE")

dat_land <- dat2 %>% filter(inside == "FALSE")

Save file with revisitation sites >250 away from waterbodies for the further analyses

write_csv(”Data/All_temp_points_land_utm.csv")

# Joining environmental data to the revisitation data

### Get Ion and lat from utm

This was needed to extract the dynamic variables from GEE

*# Load data*

data <- read_csv(“Data/All_temp_points_land_utm.csv“)

*# CLean the data*

dat2 ‹- dat %›%

mutate(ID = paste(coordIdx, id, visitIdx, sep=”-")) %>% select(ID, x, y, entranceTime) %>%

rename(fixtime = entranceTime, easting = x,

northing = y,

groupby_col = ID)

*# Transform to Lon Lab*

lat.long.df <- data.frame(dat2$easting, dat2$northing) str(lat.long.df)

coordinates(lat.long.df) ‹- -dat2.easting + dat2.northing str(lat.long.df)

proj4string(lat.long.df)

proj4string(lat.long.df) ‹- CRS(“+init=epsg:32736“) head(lat.long.df)

dist.location <- spTransform(lat.long.df, CRS(”+proj=longlat“)) dist.location

dat_lonlat <- data.frame(groupby_col = dat2$groupby_col,

lat = dist.location$dat2.northing, lon = dist.location$dat2.easting, fixtime = dat2$fixtime)

Get unique sites

This was done to speed up the extract of data from GEE for the static variables, these do not change over time so the timestamp is not needed

*# Load data*

dat ‹- read_csv(“Data/All_temp_points_land_utm.csv")

*# Get nunber of-* revtsttottons *per s ite, and onLy izeep one row per* stte

dat ‹- dat %›%

mutate(ID = paste(coordIdx, id, sep = ”-")) %>% group_by(ID) %>%

count (x, y) %›%

ungroup()

Write csv to use in GEE

write_csv(“Data/All_temp_points_land_lonlat.csv") write_csv(“Data/All_unique_points_land_utm.csv“)

Environmental data

The above file is used in Google Earth Engine (GEE) and ArcGis to extract the data of the environmental variables, which gave the following files;

Data from ArcGIS

The euclidian distance was calculated in ArcGIS

*# water*

dat ‹- read_csv(“Data/GIS/All_unique_points_land_utm.csv“) dist_to_water <- read.csv2(”Data/GIS/Dist_to_water_all_dat.csv“)

dist_water <- dat %>% left_join(dist_to_water, by = ”ID”) %>% dplyr: :select(ID, x, y, n, MIN) %>% rename(dist_to_water = MIN)

write.csv(“Data/GIS/Dist_to_water_all_dat_neat.csv“)

Static data from GEE

slope <- read_csv(“Data/GEE/elevation_all_dat.csv“) nit ‹- read_csv(“Data/GEE/N_20_and_50_all_dat.csv“) phos ‹- read_csv(“Data/GEE/P_20_and_50_all_dat.csv")

Check correlation between nutrient concentrations at the different depths

# *Nitrogen*

cor.test(nit$nitrogen_0_20, nit$nitrogen_20_50)

ggplot() +

geom_point(data = nit, aes(x = nitrogen_0_20, y = nitrogen_20_50), alpha = 0.3) +

labs(x = “nitrogen at 0 to 20 cm depth (g/kg)”, y = “nitrogen at 20 to 50 cm depth (g/kg)”) + theme(axis.title.x = element_text(size = 16),

axis.text.x = element_text(size = 14),

axis.title.y = element_text(size = 16), axis.text.y = element_text(size = 14),

axis.line.x.bottom = element_line(colour = “black"),

axis.line.y.left = element_line(colour = "black"), axis.line.y.right = element_line(colour = "black"), panel.background = element_rect(fill = "white"), panel.grid.major = element_line(color = "grey92"), panel.grid.minor = element_line(color = "grey92")) +

annotate(”text”, x=52, y=69, label= c(”Pearson's product-moment correlation"), size = 5) + annotate(”text“, x=52, y=67, label= c(”t = 3754.2, df = 717047, p-value < 2.2e-16“), size = 5)

annotate(”text“, x=52, y=65, label= c(”Correlation = 0.975“), size = 5)

*# Phosphor*

cor.test(phos$phosphor_0_20, phos$phosphor_20_50)

ggplot() +

geom_point(data = phos, aes(x = phosphor_0_20, y = phosphor_20_50), alpha = 0.3) +

labs(x = “phosphor at 0 to 20 cm depth (ppm)”, y = “phosphor at 20 to 50 cm depth (ppm)”) + theme(axis.title.x = element_text(size = 16),

axis.text.x = element_text(size = 14),

axis.title.y = element_text(size = 16), axis.text.y = element_text(size = 14),

axis.line.x.bottom = element_line(colour = “black"), axis.line.y.left = element_line(colour = "black"), axis.line.y.right = element_line(colour = "black"), panel.background = element_rect(fill = "white"), panel.grid.major = element_line(color = "grey92"), panel.grid.minor = element_line(color = "grey92")) +

annotate(”text”, x=22.5, y=27.4, label= c(“Pearson's product-moment correlation”), size = 5) + annotate(”text“, x=22.5, y=26.9, label= c(“t = 2199.1, df = 717047, p-value ‹ 2.2e-16“), size

' s)*

annotate(”text“, x=22.5, y=26.4, label= c(“Correlation = 0.933“), size = 5)

## Seasonality

Load data

season <- read_csv(”Data/season_per_day.csv”)

## Create one file, with sex and season

Load data

temp ‹- read_csv(“Data/GEE/Temperature_all_temp_points_lonlat.csv“) evi <- read_csv(“Data/GEE/EVI_all_temp_points_lonlat.csv“)

precip <- read_csv(“Data/GEE/Precipitation_all_temp_points_lonlat.csv”)

water ‹- read_csv(“Data/GIS/Dist_to_water_all_dat_neat.csv”) slope <- read_csv(“Data/GEE/elevation_all_dat.csv“)

nit <- read_csv(“Data/GEE/N_20_and_50_all_dat.csv“)

phos ‹- read_csv(“Data/GEE/P_20_and_50_all_dat.csv")

Get list of individual elephants with their sex

dat ‹- read_csv(“Data/EA/EA_WAG_elelocs_20120101-20221021_clean.csv“)

dat ‹- dat g›%

dplyr::select(groupby_col, extra subject sex)

dat <- dat[!duplicated(dat$groupby_col), ] write.csv(“Data/sex_per_groupby_col.csv“)

Join data sets

temp ‹- temp %›% mutate(ID2 = groupby_col) %>%

separate(groupby_col, c(“coordIdx“, "groupby_col", "visitldx"), sep=”-") %>% mutate(ID = paste(coordIdx, groupby_col, sep="-“)) %>%

dplyr::select(ID2, ID, LST_Day_1km, fixtime)

evi ‹- evi %›% rename(ID2 = groupby_col) %›% dplyr::select(ID2, EVI)

precip ‹- precip %>% rename(ID2 = groupby_col) %>% dplyr::select(ID2, precipitation)

slope <- slope %>% dplyr::select(ID, slope)

nit ‹- nit %›% dplyr::select(ID, nitrogen_20_50) phos <- phos %>% dplyr::select(ID, phosphor_20_50) water <- water %>% dplyr::select(ID, dist_to_water)

temp ‹- temp[!duplicated(temp$ID2), ] evi ‹- evi[!duplicated(evi$ID2), ]

precip ‹- precip[!duplicated(precip$ID2), ]

dat_tot ‹- temp %>% left_join(evi, by = "ID2") %>%

left_join(precip, by = "ID2") #%>% left_join(slope, by = "ID") %>% left_join(nit, by = "ID") %›% left_join(phos, by = "ID") %›% left_join(water, by = "ID")

write.csv(x=dat_tot,"Plot_data/dat_tot.csv“)

Add sex column

sex ‹- read_csv(“Data/sex_per_groupby_col.csv“)

dat_tot <- dat_tot %>%

separate(ID2, c(“coordIdx“, "groupby_col", "visitIdx"), sep=“-")

dat_tot ‹- dat_tot %>%

left_join(sex, by = "groupby_col") %>% rename(sex = extra subject sex)

Add season

season ‹- season %›%

mutate(date = format(date, "%Y-%m-%d"))

dat_tot ‹ - dat_tot %›%

mutate(date = format(fixtime, "%Y-%m-%d")) %>% left_join(season, by= "date")

Get number of revisitations

dat_tot ‹ - dat_tot %›fi

mutate(ID2 = paste(coordIdx, groupby_col, season, sep=“-“))

data_to_join ‹- dat_tot %>%

dplyr::select(ID2, groupby_col, season, sex, LST_Day_1km, EVI,

precipitation, slope, nitrogen_20_50, phosphor_20_50, dist_to_water)

dat_tot ‹- dat_tot %>% group_by(ID2) %>% count(coordIdx) %›% ungroup()

dat_tot ‹- dat_tot %>% left_join(data_to_join, by=“ID2”)

Transformations

*# Get per eLephant the nunber of days they are in the data*

dat_raW <- read_csv("Data/EA/EA_WAG_elelocs_20120101-20221021_clean.csv“) time_in_dat ‹- dat_raw %›%

mutate(date2 = format(fixtime, "%Y-%m-%d")) %>% group_by(groupby_col) %>%

summarise(count = n_distinct(date2))

*# Get rev is i tation* rate *per year*

dat_tot ‹- dat_tot %›%

left_join(time_in_dat, by = "groupby_col") %>% mutate(n_per_year = n/(count/365)) %>% dplyr::select(! count)

*# Transfor*/r *environnentaL variables uhen needed*

dat_adj <- dat_tot %>%

mutate(LST_Day_1km = LST_Day_1km*0.02-273, log_precip = (log10(precipitation+1)), log_slope = (log10(slope+1)), sqrt_dist_water = (sqrt(dist_to_water+250)), seasonl = season,

sex1 = sex,

season_sex = paste(season1, sex1, sep="-")) %›%

rename(nitrogen = nitrogen_20_50,

phosphor = phosphor_20_50) %>%

dplyr::select(ID2, n_per_year, groupby_col, season, sex, season_sex, LST_Day_1km, EVI, log_precip, log_slope, nitrogen, phosphor, sqrt_dist_water)

Save file for the statistical analyses

write.csv(x=dat_adj,"Plot_data/All_env_vars_tot_dat_with_temporal_all.csv“)

## Home range for random points

Load data

dat_ele <- read_csv("Data/EA/EA_WAG_elelocs_20120101-20221021_clean.csv“)

Estimate home range with a_LoCoh

*# Nabe the data an sf object*

dat_sf <- as_sf(dat_ele)

*# Checb the extent*

terra::ext(dat_sf)

*# Create enpty raster*

emptyRast ‹- terra::rast(terra: :ext(dat_sf), res=100, crs="EPSG:32736“)

*# Create vector points*

PointsAsVect ‹- vect(dat_sf)

*# Raster!ze the vector points*

rast ‹- terra::rasterize(x = PointsAsVect, y = emptyRast)

*# Nabe it into an ant tracb*

new_coordinates <- as.data.frame(rast, xy=T) %>% make_track(.x = x, .y = y, crs=32736)

*# Run with reduced data*

new_coordinates2 <- new_coordinates %>% dplyr::filter(y_ ‹ 7275985 I y > 7430554)

distances ‹- dist(new_coordinates2) MaxDistance ‹- max(distances)

*# Est!nate the hone range with a_LoCoh*

aLoc ‹- new_coordinates %>%

hr_locoh(n = MaxDistance, type = ”a“, levels = 1)

*# Extract geometry Iron aLoc object*

aLoc_geometry <- aLoc$locoh$geometry

Save file for the statistical analyses

st_write(aLoc_geometry, ”Data/GIS/Area_random_points.shp“)
