# Supplementary material for: Environmental Determinants of Foraging Site Revisitation by African Elephants ( Loxodonta africana )
Source: Ecol Evol. 2025 Jun 3;15(6):e71506. doi: 10.1002/ece3.71506 (PMC12134087; doi:10.1002/ece3.71506)
Supplement: Supplementary file 1 — Appendix S1 [file ECE3-15-e71506-s001.zip › R-Code-Statistical-Analyses.docx]

R Code - Statistical Analyses

Jacob, S.A.

2025-05-07

Load packages and data

*# Install packages*

**if**(!**require**(pacman)){

install.packages("pacman")

**library**(pacman)

}

*# Load packages*

pacman::p_load(tidyverse, recurse, lubridate, lme4, recipes, MuMIn, rsample, lmerTest, MASS, PerformanceAnalytics, r2glmm, factoextra, gridExtra, sf, data.table, sp,

ggmap, broom, interactions, ggsignif)

*# Load data*

dat <- read_csv("Data/All_env_vars_tot_dat_with_temporal_all.csv")

# Correlation between variables

cor_dat <- dat %>% group_by(ID2) %>%

slice_sample(n=1) %>%

ungroup() %>%

dplyr::select(LST_Day_1km, EVI, log_precip, log_slope, nitrogen, phosphor, sqrt_dist_water, sqrt_dist_road,

sqrt_dist_build, sqrt_dist_rail, sqrt_dist_fence) %>% rename(temperature = LST_Day_1km,

log_precipitation = log_precip,

sqrt_dist_railways = sqrt_dist_rail,

sqrt_dist_buildings = sqrt_dist_build, sqrt_dist_roads = sqrt_dist_road,

sqrt_dist_fences = sqrt_dist_fence)

resampled_data <- cor_dat[sample(nrow(cor_dat), size = nrow(cor_dat)/(nrow(cor_dat)/10000), r eplace = T), ]

chart.Correlation(resampled_data, histogram=TRUE)

Available vs selected sites

Generating random data

*# Load the aLoCoh home range around the elephant data, with a 1km buffer added and without th e buffered water area*

area <- st_read("Data/GIS/Area_random_points_no_water.shp")

*# Generate random points within the polygon*

points <- sf::st_sample(area, size=100000)

rand_point <- as.data.frame(st_coordinates(points))

rand_point <- rand_point %>% rename(easting = X, northing = Y) %>% mutate(ID = seq(1:100000))

*# Plot using the ggplot geom_sf function.*

ggplot() +

geom_sf(aes(), data=area) + geom_sf(aes(), data=points)

*# Safe the data*

write.csv("Data/Random generated points/100000_random_points_whole_area_land.csv")

Connect random time to points

*# Load elephant data to randomly select timestamps from*

dat_raw <- read_csv("Data/EA/EA_WAG_elelocs_20120101-20221021_clean.csv")

dat_rand <- read_csv("Data/Random generated points/100000_random_points_whole_area_land.cs v")

*# Get random timestamps from elephant data*

sub_dat <- sample_n(dat_raw, 100000)

sub_dat <- sub_dat %>% dplyr::select(fixtime)

*# Connect the timestamps to the data points*

rand_point <- cbind(dat_rand, sub_dat)

Prepare dataset for GEE

*# Transform utm to lon/lat*

lat.long.df <- data.frame(rand_point$easting, rand_point$northing) str(lat.long.df)

coordinates(lat.long.df) <- ~rand_point.easting + rand_point.northing str(lat.long.df)

proj4string(lat.long.df)

proj4string(lat.long.df) <- CRS("+init=epsg:32736") head(lat.long.df)

dist.location <- spTransform(lat.long.df, CRS("+proj=longlat"))

dist.location

*# Save as dataframe*

dat_lonlat <- data.frame(lat = dist.location$rand_point.northing,

lon = dist.location$rand_point.easting, fixtime = rand_point$fixtime)

*# Save data*

write.csv("Data/Random generated points/100000_random_points_whole_area_fixtime.csv")

Afterwards, the environmental values for the random points were obtained the same way as those for the revisitation sites.

### Difference between the available sites (the random data) and the selected sites (the elephant data)

A new dataset was made for the static data, where every revisitation site was only once included, as this analysis is not about the number of revisitations, and the sites do not change in values for the static

environmental variables.

*# Load data*

dat <- read_csv("Data/All_env_vars_tot_dat_with_temporal_all.csv")

*# Remove duplicate measurements*

dat <- dat[!duplicated(dat$ID2), ]

*# Save data*

write.csv("Data/All_env_vars_tot_dat_adjusted.csv")

#### Distance to water

*# Load data*

dat <- read_csv("Data/All_env_vars_tot_dat_adjusted.csv")

water_rand <- read_csv2("Data/Random generated points/Dist_to_water_rand_points.csv")

*# Plot*

ggplot() +

geom_density(data=water_rand, aes(x=(MIN +250)),

fill = "lightgray", alpha=0.7, linetype=2, linewidth=1) +

geom_density(data=dat %>% filter(season_sex == "dry-female"), aes(x=sqrt_dist_water^2), fill = "lightgray", alpha=0.15, linewidth=1, color="#F2B701") +

geom_density(data=dat %>% filter(season_sex == "wet-female"), aes(x=sqrt_dist_water^2), fill = "lightgray", alpha=0.15, linewidth=1, color="#009988") +

geom_density(data=dat %>% filter(season_sex == "dry-male"), aes(x=sqrt_dist_water^2), fill = "lightgray", alpha=0.15, linewidth=1, color="#E65518") +

geom_density(data=dat %>% filter(season_sex == "wet-male"), aes(x=sqrt_dist_water^2),

fill = "lightgray", alpha=0.15, linewidth=1, color="#88CCEE") + scale_x_sqrt()+

scale_y_continuous("density") +

labs(x = "distance to water (m)") +

theme( axis.title.x = element_text(size = 16), axis.text.x = element_text(size = 14), axis.title.y = element_text(size = 16), axis.text.y = element_text(size = 14),

axis.line.x.bottom = element_line(colour = "black"), axis.line.y.left = element_line(colour = "black"),

axis.line.y.right = element_line(colour = "black"),

panel.background = element_rect(fill = "white"), panel.grid.major = element_line(color = "grey92"), panel.grid.minor = element_line(color = "grey92"))

Kolmogorov test

*# Transform random data*

water_rand <- water_rand %>% mutate(sqrt_dist_water = sqrt(MIN+250))

*# Cows, dry season*

obs_dat <- dat %>% filter(season_sex == "dry-female") obs_dat <- obs_dat$sqrt_dist_water

rand_dat <- water_rand$sqrt_dist_water

ks.test(obs_dat, rand_dat)

*# Visualization*

plot(ecdf((obs_dat)),

xlim = range(c((obs_dat), (rand_dat))), col = "blue",

xlab="distance to water (sqrt(m))",ylab="cumulative percentage", main="Cows, dry seaso

n", cex.main=1)

plot(ecdf((rand_dat)), add = TRUE,

lty = "dashed",

col = "red")

legend("bottomright", legend=c("Observed data", "Random data"), col=c("blue", "red"), lty=1, cex=0.8)

*# Define the number of iterations*

n_iterations <- 10000

*# Create an empty vector to store the R2 values*

p_value <- numeric(n_iterations) D <- numeric(n_iterations)

ktest_values <- as.data.frame(cbind(D, p_value))

*# Looping the Kolmogorov test*

dat_wf <- dat %>% filter(season_sex == "dry-female")

**for** (i **in** 1:n_iterations) {

*# Get subsample*

obs_dat <- sample(dat_wf$sqrt_dist_water, size = 100, replace = F)

rand_dat <- sample(water_rand$sqrt_dist_water, size = 100, replace = F)

*# Kolmogorof test*

k_test <- ks.test((obs_dat), (rand_dat))

*# Calculate the values and store it in the vector*

ktest_values[i,1] <- k_test$statistic ktest_values[i,2] <- k_test$p.value

}

*# Test values*

mean(ktest_values$p_value) sd(ktest_values$p_value)

mean(ktest_values$D) sd(ktest_values$D)

*# Cows, wet season*

obs_dat <- dat %>% filter(season_sex == "wet-female") obs_dat <- obs_dat$sqrt_dist_water

rand_dat <- water_rand$sqrt_dist_water

ks.test(obs_dat, rand_dat)

*# Visualization*

plot(ecdf((obs_dat)),

xlim = range(c((obs_dat), (rand_dat))), col = "blue",

xlab="distance to water (sqrt(m))",ylab="cumulative percentage", main="Cows, wet seaso

n", cex.main=1)

plot(ecdf((rand_dat)), add = TRUE,

lty = "dashed",

col = "red")

legend("bottomright", legend=c("Observed data", "Random data"), col=c("blue", "red"), lty=1, cex=0.8)

*# Define the number of iterations*

n_iterations <- 10000

*# Create an empty vector to store the R2 values*

p_value <- numeric(n_iterations) D <- numeric(n_iterations)

ktest_values <- as.data.frame(cbind(D, p_value))

*# Looping the Kolmogorov test*

dat_wf <- dat %>% filter(season_sex == "wet-female")

**for** (i **in** 1:n_iterations) {

*# Get subsample*

obs_dat <- sample(dat_wf$sqrt_dist_water, size = 100, replace = F)

rand_dat <- sample(water_rand$sqrt_dist_water, size = 100, replace = F)

*# Kolmogorof test*

k_test <- ks.test((obs_dat), (rand_dat))

*# Calculate the values and store it in the vector*

ktest_values[i,1] <- k_test$statistic ktest_values[i,2] <- k_test$p.value

}

*# Test values*

mean(ktest_values$p_value) sd(ktest_values$p_value)

mean(ktest_values$D) sd(ktest_values$D)

*# Bulls, dry season*

obs_dat <- dat %>% filter(season_sex == "dry-male") obs_dat <- obs_dat$sqrt_dist_water

rand_dat <- water_rand$sqrt_dist_water

ks.test(obs_dat, rand_dat)

*# Visualization*

plot(ecdf((obs_dat)),

xlim = range(c((obs_dat), (rand_dat))), col = "blue",

xlab="distance to water (sqrt(m))",ylab="cumulative percentage", main="Bulls, dry seaso

n", cex.main=1)

plot(ecdf((rand_dat)), add = TRUE,

lty = "dashed",

col = "red")

legend("bottomright", legend=c("Observed data", "Random data"), col=c("blue", "red"), lty=1, cex=0.8)

*# Define the number of iterations*

n_iterations <- 10000

*# Create an empty vector to store the R2 values*

p_value <- numeric(n_iterations) D <- numeric(n_iterations)

ktest_values <- as.data.frame(cbind(D, p_value))

*# Looping the Kolmogorov test*

dat_wf <- dat %>% filter(season_sex == "dry-male")

**for** (i **in** 1:n_iterations) {

*# Get subsample*

obs_dat <- sample(dat_wf$sqrt_dist_water, size = 100, replace = F)

rand_dat <- sample(water_rand$sqrt_dist_water, size = 100, replace = F)

*# Kolmogorof test*

k_test <- ks.test((obs_dat), (rand_dat))

*# Calculate the values and store it in the vector*

ktest_values[i,1] <- k_test$statistic ktest_values[i,2] <- k_test$p.value

}

*# Test values*

mean(ktest_values$p_value) sd(ktest_values$p_value)

mean(ktest_values$D) sd(ktest_values$D)

*# Bulls, wet season*

obs_dat <- dat %>% filter(season_sex == "wet-male") obs_dat <- obs_dat$sqrt_dist_water

rand_dat <- water_rand$sqrt_dist_water

ks.test(obs_dat, rand_dat)

*# Visualization*

plot(ecdf((obs_dat)),

xlim = range(c((obs_dat), (rand_dat))), col = "blue",

xlab="distance to water (sqrt(m))",ylab="cumulative percentage", main="Bulls, wet seaso

n", cex.main=1)

plot(ecdf((rand_dat)), add = TRUE,

lty = "dashed",

col = "red")

legend("bottomright", legend=c("Observed data", "Random data"), col=c("blue", "red"), lty=1, cex=0.8)

*# Define the number of iterations*

n_iterations <- 10000

*# Create an empty vector to store the R2 values*

p_value <- numeric(n_iterations) D <- numeric(n_iterations)

ktest_values <- as.data.frame(cbind(D, p_value))

*# Looping the Kolmogorov test*

dat_wf <- dat %>% filter(season_sex == "wet-male")

**for** (i **in** 1:n_iterations) {

*# Get subsample*

obs_dat <- sample(dat_wf$sqrt_dist_water, size = 100, replace = F)

rand_dat <- sample(water_rand$sqrt_dist_water, size = 100, replace = F)

*# Kolmogorof test*

k_test <- ks.test((obs_dat), (rand_dat))

*# Calculate the values and store it in the vector*

ktest_values[i,1] <- k_test$statistic ktest_values[i,2] <- k_test$p.value

}

*# Test values*

mean(ktest_values$p_value) sd(ktest_values$p_value)

mean(ktest_values$D) sd(ktest_values$D)

#### EVI

*# Load data*

dat <- read_csv("Data/All_env_vars_tot_dat_with_temporal_all.csv")

rand_evi <- read_csv("Data/Random generated points/EVI_rand_point.csv") season <- read_csv("Data/season_per_day.csv")

*# Get season in random data*

season <- season %>%

mutate(date = format(date, "%d-%m-%Y"))

rand_evi <- rand_evi %>%

dplyr::select(fixtime, EVI) %>%

separate(fixtime, c("date", "time"), sep = " ") %>% separate(date, c("year", "month", "day"), sep="-")

rand_evi$day <- as.integer(rand_evi$day)

rand_evi$month <- as.integer(rand_evi$month)

rand_evi <- rand_evi %>% mutate(zero = "0",

day2 = ifelse(day<10, paste(zero, day, sep = ""), day),

month2 = ifelse(month<10, paste(zero, month, sep = ""), month), date = paste(day2, month2, year, sep = "-")) %>%

left_join(season, by= "date") %>%

mutate(id = paste(date, time, EVI))

rand_evi <- rand_evi[!duplicated(rand_evi$id), ] evi_rand <- na.omit(rand_evi)

*# Plot*

ggplot() +

geom_density(data=evi_rand%>% filter(season == "dry"), aes(x=(EVI*0.0001)),

fill = "lightgray", alpha=0.25, linetype=2, linewidth=1, color="#600000") + geom_density(data=evi_rand%>% filter(season == "wet"), aes(x=(EVI*0.0001)),

fill = "lightgray", alpha=0.25, linetype=2, linewidth=1, color="darkblue") +

geom_density(data=dat %>% filter(season_sex == "dry-female"), aes(x=EVI*0.0001), fill = "lightgray", alpha=0.15, linewidth=1, color="#F2B701") +

geom_density(data=dat %>% filter(season_sex == "wet-female"), aes(x=EVI*0.0001), fill = "lightgray", alpha=0.15, linewidth=1, color="#009988") +

geom_density(data=dat %>% filter(season_sex == "dry-male"), aes(x=EVI*0.0001),

fill = "lightgray", alpha=0.15, linewidth=1, color="#E65518") + geom_density(data=dat %>% filter(season_sex == "wet-male"), aes(x=EVI*0.0001),

fill = "lightgray", alpha=0.15, linewidth=1, color="#88CCEE") + scale_y_continuous("density") +

labs(x = "EVI") +

theme(axis.title.x = element_text(size = 16), axis.text.x = element_text(size = 14), axis.title.y = element_text(size = 16), axis.text.y = element_text(size = 14),

axis.line.x.bottom = element_line(colour = "black"), axis.line.y.left = element_line(colour = "black"),

axis.line.y.right = element_line(colour = "black"), panel.background = element_rect(fill = "white"),

panel.grid.major = element_line(color = "grey92"), panel.grid.minor = element_line(color = "grey92"))

Kolmogorov test

*# Cows, dry season*

obs_dat <- dat %>% filter(season_sex == "dry-female") obs_dat <- obs_dat$EVI

rand_dat <- evi_rand %>% filter(season == "dry")

rand_dat <- rand_dat$EVI

ks.test(obs_dat, rand_dat)

*# Visualization*

plot(ecdf((obs_dat)),

xlim = range(c((obs_dat), (rand_dat))), col = "blue",

xlab="EVI",ylab="cumulative percentage", main="Cows, dry season", cex.main=1) plot(ecdf((rand_dat)),

add = TRUE,

lty = "dashed", col = "red")

legend("bottomright", legend=c("Observed data", "Random data"), col=c("blue", "red"), lty=1, cex=0.8)

*# Define the number of iterations*

n_iterations <- 10000

*# Create an empty vector to store the R2 values*

p_value <- numeric(n_iterations) D <- numeric(n_iterations)

ktest_values <- as.data.frame(cbind(D, p_value))

*# Looping the Kolmogorov test*

dat_wf <- dat %>% filter(season_sex == "dry-female") rand_dry <- evi_rand %>% filter(season == "dry")

**for** (i **in** 1:n_iterations) {

*# Get subsample*

obs_dat <- sample(dat_wf$EVI, size = 100, replace = F)

rand_dat <- sample(rand_dry$EVI, size = 100, replace = F)

*# Kolmogorof test*

k_test <- ks.test((obs_dat), (rand_dat))

*# Calculate the values and store it in the vector*

ktest_values[i,1] <- k_test$statistic ktest_values[i,2] <- k_test$p.value

}

*# Test values*

mean(ktest_values$p_value) sd(ktest_values$p_value)

mean(ktest_values$D) sd(ktest_values$D)

*# Cows, wet season*

obs_dat <- dat %>% filter(season_sex == "wet-female") obs_dat <- obs_dat$EVI

rand_dat <- evi_rand %>% filter(season == "wet")

rand_dat <- rand_dat$EVI

ks.test(obs_dat, rand_dat)

*# Visualization*

plot(ecdf((obs_dat)),

xlim = range(c((obs_dat), (rand_dat))), col = "blue",

xlab="EVI",ylab="cumulative percentage", main="Cows, wet season", cex.main=1) plot(ecdf((rand_dat)),

add = TRUE,

lty = "dashed", col = "red")

legend("bottomright", legend=c("Observed data", "Random data"), col=c("blue", "red"), lty=1, cex=0.8)

*# Define the number of iterations*

n_iterations <- 10000

*# Create an empty vector to store the R2 values*

p_value <- numeric(n_iterations) D <- numeric(n_iterations)

ktest_values <- as.data.frame(cbind(D, p_value))

*# Looping the Kolmogorov test*

dat_wf <- dat %>% filter(season_sex == "wet-female") rand_dry <- evi_rand %>% filter(season == "wet")

**for** (i **in** 1:n_iterations) {

*# Get subsample*

obs_dat <- sample(dat_wf$EVI, size = 100, replace = F)

rand_dat <- sample(rand_dry$EVI, size = 100, replace = F)

*# Kolmogorof test*

k_test <- ks.test((obs_dat), (rand_dat))

*# Calculate the values and store it in the vector*

ktest_values[i,1] <- k_test$statistic ktest_values[i,2] <- k_test$p.value

}

*# Test values*

mean(ktest_values$p_value) sd(ktest_values$p_value)

mean(ktest_values$D) sd(ktest_values$D)

*# Bulls, dry season*

obs_dat <- dat %>% filter(season_sex == "dry-male") obs_dat <- obs_dat$EVI

rand_dat <- evi_rand %>% filter(season == "dry")

rand_dat <- rand_dat$EVI

ks.test(obs_dat, rand_dat)

*# Visualization*

plot(ecdf((obs_dat)),

xlim = range(c((obs_dat), (rand_dat))), col = "blue",

xlab="EVI",ylab="cumulative percentage", main="Bulls, dry season", cex.main=1) plot(ecdf((rand_dat)),

add = TRUE,

lty = "dashed", col = "red")

legend("bottomright", legend=c("Observed data", "Random data"), col=c("blue", "red"), lty=1, cex=0.8)

*# Define the number of iterations*

n_iterations <- 10000

*# Create an empty vector to store the R2 values*

p_value <- numeric(n_iterations) D <- numeric(n_iterations)

ktest_values <- as.data.frame(cbind(D, p_value))

*# Looping the Kolmogorov test*

dat_wf <- dat %>% filter(season_sex == "dry-male") rand_dry <- evi_rand %>% filter(season == "dry")

**for** (i **in** 1:n_iterations) {

*# Get subsample*

obs_dat <- sample(dat_wf$EVI, size = 100, replace = F)

rand_dat <- sample(rand_dry$EVI, size = 100, replace = F)

*# Kolmogorof test*

k_test <- ks.test((obs_dat), (rand_dat))

*# Calculate the values and store it in the vector*

ktest_values[i,1] <- k_test$statistic ktest_values[i,2] <- k_test$p.value

}

*# Test values*

mean(ktest_values$p_value) sd(ktest_values$p_value)

mean(ktest_values$D) sd(ktest_values$D)

*# Bulls, wet season*

obs_dat <- dat %>% filter(season_sex == "wet-male") obs_dat <- obs_dat$EVI

rand_dat <- evi_rand %>% filter(season == "wet")

rand_dat <- rand_dat$EVI

ks.test(obs_dat, rand_dat)

*# Visualization*

obs_dat <- na.omit(obs_dat) plot(ecdf((obs_dat)),

xlim = range(c((obs_dat), (rand_dat))),

col = "blue",

xlab="EVI",ylab="cumulative percentage", main="Bulls, wet season", cex.main=1) plot(ecdf((rand_dat)),

add = TRUE,

lty = "dashed", col = "red")

legend("bottomright", legend=c("Observed data", "Random data"),

col=c("blue", "red"), lty=1, cex=0.8)

*# Define the number of iterations*

n_iterations <- 10000

*# Create an empty vector to store the R2 values*

p_value <- numeric(n_iterations) D <- numeric(n_iterations)

ktest_values <- as.data.frame(cbind(D, p_value))

*# Looping the Kolmogorov test*

dat_wf <- dat %>% filter(season_sex == "wet-male") rand_dry <- evi_rand %>% filter(season == "wet")

**for** (i **in** 1:n_iterations) {

*# Get subsample*

obs_dat <- sample(dat_wf$EVI, size = 100, replace = F)

rand_dat <- sample(rand_dry$EVI, size = 100, replace = F)

*# Kolmogorof test*

k_test <- ks.test((obs_dat), (rand_dat))

*# Calculate the values and store it in the vector*

ktest_values[i,1] <- k_test$statistic ktest_values[i,2] <- k_test$p.value

}

*# Test values*

mean(ktest_values$p_value) sd(ktest_values$p_value)

mean(ktest_values$D)

sd(ktest_values$D)

#### Phosphor

*# Load data*

dat <- read_csv("Data/All_env_vars_tot_dat_adjusted.csv")

phos_rand <- read_csv("Data/Random generated points/P_20_and_50_rand_point.csv")

*# Plot*

ggplot() +

geom_density(data=phos_rand, aes(x=(phosphor_20_50)),

fill = "lightgray", alpha=0.7, linetype=2, linewidth=1) +

geom_density(data=dat %>% filter(season_sex == "dry-female"), aes(x=phosphor), fill = "lightgray", alpha=0.15, linewidth=1, color="#F2B701") +

geom_density(data=dat %>% filter(season_sex == "wet-female"), aes(x=phosphor), fill = "lightgray", alpha=0.15, linewidth=1, color="#009988") +

geom_density(data=dat %>% filter(season_sex == "dry-male"), aes(x=phosphor), fill = "lightgray", alpha=0.15, linewidth=1, color="#E65518") +

geom_density(data=dat %>% filter(season_sex == "wet-male"), aes(x=phosphor), fill = "lightgray", alpha=0.15, linewidth=1, color="#88CCEE") +

scale_y_continuous("density") + labs(x = "phosphorus (ppm)") +

theme(axis.title.x = element_text(size = 16),

axis.text.x = element_text(size = 14), axis.title.y = element_text(size = 16), axis.text.y = element_text(size = 14),

axis.line.x.bottom = element_line(colour = "black"), axis.line.y.left = element_line(colour = "black"),

axis.line.y.right = element_line(colour = "black"),

panel.background = element_rect(fill = "white"), panel.grid.major = element_line(color = "grey92"), panel.grid.minor = element_line(color = "grey92"))

Kolmogorov test

*# Cows, dry season*

obs_dat <- dat %>% filter(season_sex == "dry-female") obs_dat <- obs_dat$phosphor

rand_dat <- phos_rand$phosphor_20_50

ks.test(obs_dat, rand_dat)

*# Visualization*

plot(ecdf((obs_dat)),

xlim = range(c((obs_dat), (rand_dat))), col = "blue",

xlab="phosphor (ppm)",ylab="cumulative percentage", main="Cows, dry season", cex.main=1)

plot(ecdf((rand_dat)), add = TRUE,

lty = "dashed", col = "red")

legend("bottomright", legend=c("Observed data", "Random data"), col=c("blue", "red"), lty=1, cex=0.8)

*# Define the number of iterations*

n_iterations <- 10000

*# Create an empty vector to store the R2 values*

p_value <- numeric(n_iterations) D <- numeric(n_iterations)

ktest_values <- as.data.frame(cbind(D, p_value))

*# Looping the Kolmogorov test*

dat_wf <- dat %>% filter(season_sex == "dry-female")

**for** (i **in** 1:n_iterations) {

*# Get subsample*

obs_dat <- sample(dat_wf$phosphor, size = 100, replace = F)

rand_dat <- sample(phos_rand$phosphor_20_50, size = 100, replace = F)

*# Kolmogorof test*

k_test <- ks.test((obs_dat), (rand_dat))

*# Calculate the values and store it in the vector*

ktest_values[i,1] <- k_test$statistic ktest_values[i,2] <- k_test$p.value

}

*# Test values*

mean(ktest_values$p_value) sd(ktest_values$p_value)

mean(ktest_values$D)

sd(ktest_values$D)

*# Cows, wet season*

obs_dat <- dat %>% filter(season_sex == "wet-female") obs_dat <- obs_dat$phosphor

rand_dat <- phos_rand$phosphor_20_50

ks.test(obs_dat, rand_dat)

*# Visualization*

plot(ecdf((obs_dat)),

xlim = range(c((obs_dat), (rand_dat))), col = "blue",

xlab="phosphor (ppm)",ylab="cumulative percentage", main="Cows, wet season", cex.main=1)

plot(ecdf((rand_dat)), add = TRUE,

lty = "dashed", col = "red")

legend("bottomright", legend=c("Observed data", "Random data"), col=c("blue", "red"), lty=1, cex=0.8)

*# Define the number of iterations*

n_iterations <- 10000

*# Create an empty vector to store the R2 values*

p_value <- numeric(n_iterations) D <- numeric(n_iterations)

ktest_values <- as.data.frame(cbind(D, p_value))

*# Looping the Kolmogorov test*

dat_wf <- dat %>% filter(season_sex == "wet-female")

**for** (i **in** 1:n_iterations) {

*# Get subsample*

obs_dat <- sample(dat_wf$phosphor, size = 100, replace = F)

rand_dat <- sample(phos_rand$phosphor_20_50, size = 100, replace = F)

*# Kolmogorof test*

k_test <- ks.test((obs_dat), (rand_dat))

*# Calculate the values and store it in the vector*

ktest_values[i,1] <- k_test$statistic ktest_values[i,2] <- k_test$p.value

}

*# Test values*

mean(ktest_values$p_value) sd(ktest_values$p_value)

mean(ktest_values$D)

sd(ktest_values$D)

*# Bulls, dry season*

obs_dat <- dat %>% filter(season_sex == "dry-male") obs_dat <- obs_dat$phosphor

rand_dat <- phos_rand$phosphor_20_50

ks.test(obs_dat, rand_dat)

*# Visualization*

plot(ecdf((obs_dat)),

xlim = range(c((obs_dat), (rand_dat))), col = "blue",

xlab="phosphor (ppm)",ylab="cumulative percentage", main="Bulls, dry season", cex.main=

1)

plot(ecdf((rand_dat)), add = TRUE,

lty = "dashed",

col = "red")

legend("bottomright", legend=c("Observed data", "Random data"), col=c("blue", "red"), lty=1, cex=0.8)

*# Define the number of iterations*

n_iterations <- 10000

*# Create an empty vector to store the R2 values*

p_value <- numeric(n_iterations) D <- numeric(n_iterations)

ktest_values <- as.data.frame(cbind(D, p_value))

*# Looping the Kolmogorov test*

dat_wf <- dat %>% filter(season_sex == "dry-male")

**for** (i **in** 1:n_iterations) {

*# Get subsample*

obs_dat <- sample(dat_wf$phosphor, size = 100, replace = F)

rand_dat <- sample(phos_rand$phosphor_20_50, size = 100, replace = F)

*# Kolmogorof test*

k_test <- ks.test((obs_dat), (rand_dat))

*# Calculate the values and store it in the vector*

ktest_values[i,1] <- k_test$statistic ktest_values[i,2] <- k_test$p.value

}

*# Test values*

mean(ktest_values$p_value) sd(ktest_values$p_value)

mean(ktest_values$D) sd(ktest_values$D)

*# Bulls, wet season*

obs_dat <- dat %>% filter(season_sex == "wet-male") obs_dat <- obs_dat$phosphor

rand_dat <- phos_rand$phosphor_20_50

ks.test(obs_dat, rand_dat)

*# Visualization*

plot(ecdf((obs_dat)),

xlim = range(c((obs_dat), (rand_dat))), col = "blue",

xlab="phosphor (ppm)",ylab="cumulative percentage", main="Bulls, wet season", cex.main=

1)

plot(ecdf((rand_dat)), add = TRUE,

lty = "dashed",

col = "red")

legend("bottomright", legend=c("Observed data", "Random data"), col=c("blue", "red"), lty=1, cex=0.8)

*# Define the number of iterations*

n_iterations <- 10000

*# Create an empty vector to store the R2 values*

p_value <- numeric(n_iterations) D <- numeric(n_iterations)

ktest_values <- as.data.frame(cbind(D, p_value))

*# Looping the Kolmogorov test*

dat_wf <- dat %>% filter(season_sex == "wet-male")

**for** (i **in** 1:n_iterations) {

*# Get subsample*

obs_dat <- sample(dat_wf$phosphor, size = 100, replace = F)

rand_dat <- sample(phos_rand$phosphor_20_50, size = 100, replace = F)

*# Kolmogorof test*

k_test <- ks.test((obs_dat), (rand_dat))

*# Calculate the values and store it in the vector*

ktest_values[i,1] <- k_test$statistic ktest_values[i,2] <- k_test$p.value

}

*# Test values*

mean(ktest_values$p_value) sd(ktest_values$p_value)

mean(ktest_values$D) sd(ktest_values$D)

#### Precipitation

*# Load data*

dat <- read_csv("Data/All_env_vars_tot_dat_with_temporal_all.csv")

rand_precip <- read_csv("Data/Random generated points/precip_rand_point.csv") season <- read_csv("Data/season_per_day.csv")

*# Get season in random data*

season <- season %>%

mutate(date = format(date, "%d-%m-%Y"))

rand_precip <- rand_precip %>%

dplyr::select(fixtime, precipitation) %>%

separate(fixtime, c("date", "time"), sep = " ") %>% separate(date, c("year", "month", "day"), sep="-")

rand_precip$day <- as.integer(rand_precip$day)

rand_precip$month <- as.integer(rand_precip$month)

rand_precip <- rand_precip %>% mutate(zero = "0",

day2 = ifelse(day<10, paste(zero, day, sep = ""), day),

month2 = ifelse(month<10, paste(zero, month, sep = ""), month), date = paste(day2, month2, year, sep = "-")) %>%

left_join(season, by= "date") %>%

mutate(id = paste(date, time, precipitation))

rand_precip <- rand_precip[!duplicated(rand_precip$id), ] prec_rand <- na.omit(rand_precip)

*# Plot*

ggplot() +

geom_density(data=prec_rand%>% filter(season == "dry"), aes(x=(precipitation+1)),

fill = "lightgray", alpha=0.25, linetype=2, linewidth=1, color="#600000") + geom_density(data=prec_rand%>% filter(season == "wet"), aes(x=(precipitation+1)),

fill = "lightgray", alpha=0.25, linetype=2, linewidth=1, color="darkblue") +

geom_density(data=dat %>% filter(season_sex == "dry-female"), aes(x=10^log_precip), fill = "lightgray", alpha=0.15, linewidth=1, color="#F2B701") +

geom_density(data=dat %>% filter(season_sex == "wet-female"), aes(x=10^log_precip), fill = "lightgray", alpha=0.15, linewidth=1, color="#009988") +

geom_density(data=dat %>% filter(season_sex == "dry-male"), aes(x=10^log_precip),

fill = "lightgray", alpha=0.15, linewidth=1, color="#E65518") +

geom_density(data=dat %>% filter(season_sex == "wet-male"), aes(x=10^log_precip), fill = "lightgray", alpha=0.15, linewidth=1, color="#88CCEE") +

scale_y_continuous("density") +

scale_x_log10() +

labs(x = "precipitation (mm/5 days)") +

theme(axis.title.x = element_text(size = 16), axis.text.x = element_text(size = 14), axis.title.y = element_text(size = 16), axis.text.y = element_text(size = 14),

axis.line.x.bottom = element_line(colour = "black"), axis.line.y.left = element_line(colour = "black"),

axis.line.y.right = element_line(colour = "black"),

panel.background = element_rect(fill = "white"), panel.grid.major = element_line(color = "grey92"), panel.grid.minor = element_line(color = "grey92"))

Kolmogorov test

*# Cows, dry season*

obs_dat <- dat %>% filter(season_sex == "dry-female") obs_dat <- obs_dat$log_precip

rand_dat <- prec_rand %>% filter(season == "dry")

rand_dat <- rand_dat$precipitation ks.test(obs_dat, log10(rand_dat+1))

*# Visualization*

plot(ecdf(obs_dat),

xlim = range(c(obs_dat, obs_dat)), col = "blue",

xlab="precipitation (mm/5 days)",ylab="cumulative percentage", main="Cows, dry season", cex.main=1)

plot(ecdf(log10(rand_dat+1)), add = TRUE,

lty = "dashed", col = "red")

legend("bottomright", legend=c("Observed data", "Random data"),

col=c("blue", "red"), lty=1, cex=0.8)

*# Define the number of iterations*

n_iterations <- 10000

*# Create an empty vector to store the R2 values*

p_value <- numeric(n_iterations) D <- numeric(n_iterations)

ktest_values <- as.data.frame(cbind(D, p_value))

*# Looping the Kolmogorov test*

dat_wf <- dat %>% filter(season_sex == "dry-female") rand_w <- prec_rand %>% filter(season == "dry")

**for** (i **in** 1:n_iterations) {

*# Get subsample*

obs_dat <- sample(dat_wf$log_precip, size = 100, replace = F)

rand_dat <- sample(rand_w$precipitation, size = 100, replace = F)

*# Kolmogorof test*

k_test <- ks.test(obs_dat, log10(rand_dat+1))

*# Calculate the values and store it in the vector*

ktest_values[i,1] <- k_test$statistic ktest_values[i,2] <- k_test$p.value

}

*# Test values*

mean(ktest_values$p_value) sd(ktest_values$p_value)

mean(ktest_values$D)

sd(ktest_values$D)

*# Cows, wet season*

obs_dat <- dat %>% filter(season_sex == "wet-female") obs_dat <- obs_dat$log_precip

rand_dat <- prec_rand %>% filter(season == "wet")

rand_dat <- rand_dat$precipitation ks.test(obs_dat, log10(rand_dat+1))

*# Visualization*

plot(ecdf(obs_dat),

xlim = range(c(obs_dat, rand_dat)), col = "blue",

xlab="precipitation (mm/5 days)",ylab="cumulative percentage", main="Cows, wet season", cex.main=1)

plot(ecdf(log10(rand_dat+1)), add = TRUE,

lty = "dashed", col = "red")

legend("bottomright", legend=c("Observed data", "Random data"),

col=c("blue", "red"), lty=1, cex=0.8)

*# Define the number of iterations*

n_iterations <- 10000

*# Create an empty vector to store the R2 values*

p_value <- numeric(n_iterations) D <- numeric(n_iterations)

ktest_values <- as.data.frame(cbind(D, p_value))

*# Looping the Kolmogorov test*

dat_wf <- dat %>% filter(season_sex == "wet-female") rand_w <- prec_rand %>% filter(season == "wet")

**for** (i **in** 1:n_iterations) {

*# Get subsample*

obs_dat <- sample(dat_wf$log_precip, size = 100, replace = F)

rand_dat <- sample(rand_w$precipitation, size = 100, replace = F)

*# Kolmogorof test*

k_test <- ks.test(obs_dat, log10(rand_dat+1))

*# Calculate the values and store it in the vector*

ktest_values[i,1] <- k_test$statistic ktest_values[i,2] <- k_test$p.value

}

*# Test values*

mean(ktest_values$p_value) sd(ktest_values$p_value)

mean(ktest_values$D)

sd(ktest_values$D)

*# Bulls, dry season*

obs_dat <- dat %>% filter(season_sex == "dry-male") obs_dat <- obs_dat$log_precip

rand_dat <- prec_rand %>% filter(season == "dry")

rand_dat <- rand_dat$precipitation ks.test(obs_dat, log10(rand_dat+1))

*# Visualization*

plot(ecdf(obs_dat),

xlim = range(c(obs_dat, obs_dat)), col = "blue",

xlab="precipitation (mm/5 days)",ylab="cumulative percentage", main="Bulls, dry season", cex.main=1)

plot(ecdf(log10(rand_dat+1)), add = TRUE,

lty = "dashed", col = "red")

legend("bottomright", legend=c("Observed data", "Random data"),

col=c("blue", "red"), lty=1, cex=0.8)

*# Define the number of iterations*

n_iterations <- 10000

*# Create an empty vector to store the R2 values*

p_value <- numeric(n_iterations) D <- numeric(n_iterations)

ktest_values <- as.data.frame(cbind(D, p_value))

*# Looping the Kolmogorov test*

dat_wf <- dat %>% filter(season_sex == "dry-male") rand_w <- prec_rand %>% filter(season == "dry")

**for** (i **in** 1:n_iterations) {

*# Get subsample*

obs_dat <- sample(dat_wf$log_precip, size = 100, replace = F)

rand_dat <- sample(rand_w$precipitation, size = 100, replace = F)

*# Kolmogorof test*

k_test <- ks.test(obs_dat, log10(rand_dat+1))

*# Calculate the values and store it in the vector*

ktest_values[i,1] <- k_test$statistic ktest_values[i,2] <- k_test$p.value

}

*# Test values*

mean(ktest_values$p_value) sd(ktest_values$p_value)

mean(ktest_values$D)

sd(ktest_values$D)

*# Bulls, wet season*

obs_dat <- dat %>% filter(season_sex == "wet-male") obs_dat <- obs_dat$log_precip

rand_dat <- water_rand %>% filter(season == "wet")

rand_dat <- rand_dat$precipitation ks.test(obs_dat, log10(rand_dat+1))

*# Visualization*

plot(ecdf(obs_dat),

xlim = range(c(obs_dat, obs_dat)), col = "blue",

xlab="precipitation (mm/5 days)",ylab="cumulative percentage", main="Bulls, wet season", cex.main=1)

plot(ecdf(log10(rand_dat+1)), add = TRUE,

lty = "dashed", col = "red")

legend("bottomright", legend=c("Observed data", "Random data"),

col=c("blue", "red"), lty=1, cex=0.8)

*# Define the number of iterations*

n_iterations <- 10000

*# Create an empty vector to store the R2 values*

p_value <- numeric(n_iterations) D <- numeric(n_iterations)

ktest_values <- as.data.frame(cbind(D, p_value))

*# Looping the Kolmogorov test*

dat_wf <- dat %>% filter(season_sex == "wet-male") rand_w <- water_rand %>% filter(season == "wet")

**for** (i **in** 1:n_iterations) {

*# Get subsample*

obs_dat <- sample(dat_wf$log_precip, size = 100, replace = F)

rand_dat <- sample(rand_w$precipitation, size = 100, replace = F)

*# Kolmogorof test*

k_test <- ks.test(obs_dat, log10(rand_dat+1))

*# Calculate the values and store it in the vector*

ktest_values[i,1] <- k_test$statistic ktest_values[i,2] <- k_test$p.value

}

*# Test values*

mean(ktest_values$p_value) sd(ktest_values$p_value)

mean(ktest_values$D)

sd(ktest_values$D)

#### Slope

*# Load data*

dat <- read_csv("Data/All_env_vars_tot_dat_adjusted.csv")

slope_rand <- read_csv("Data/Random generated points/elevation_rand_point.csv")

*# Plot*

ggplot() +

geom_density(data=slope_rand, aes(x=(slope+1)),

fill = "lightgray", alpha = 0.7, linetype=2, linewidth=1) +

geom_density(data=dat %>% filter(season_sex == "dry-female"), aes(x=10^log_slope), fill = "lightgray", alpha=0.15, linewidth=1.4, color="#F2B701") +

geom_density(data=dat %>% filter(season_sex == "wet-female"), aes(x=10^log_slope), fill = "lightgray", alpha=0.15, linewidth=1, color="#009988") +

geom_density(data=dat %>% filter(season_sex == "dry-male"), aes(x=10^log_slope), fill = "lightgray", alpha=0.15, linewidth=1.2, color="#E65518") +

geom_density(data=dat %>% filter(season_sex == "wet-male"), aes(x=10^log_slope), fill = "lightgray", alpha=0.15, linewidth=1, color="#88CCEE") +

scale_x_log10() +

scale_y_continuous("density") + labs(x = "slope (degree)") +

theme(axis.title.x = element_text(size = 16), axis.text.x = element_text(size = 14), axis.title.y = element_text(size = 16), axis.text.y = element_text(size = 14),

axis.line.x.bottom = element_line(colour = "black"), axis.line.y.left = element_line(colour = "black"),

axis.line.y.right = element_line(colour = "black"), panel.background = element_rect(fill = "white"),

panel.grid.major = element_line(color = "grey92"),

panel.grid.minor = element_line(color = "grey92"))

Kolmogorov test

*# Transform random data*

slope_rand <- slope_rand %>% mutate(log_slope = log10(slope +1))

*# Cows, dry season*

obs_dat <- dat %>% filter(season_sex == "dry-female") obs_dat <- obs_dat$log_slope

rand_dat <- slope_rand$log_slope

ks.test(obs_dat, rand_dat)

*# Visualization*

plot(ecdf((obs_dat)),

xlim = range(c((obs_dat), (rand_dat))), col = "blue",

xlab="slope (log10(º))",ylab="cumulative percentage", main="Cows, dry season", cex.main=

1)

plot(ecdf((rand_dat)), add = TRUE,

lty = "dashed",

col = "red")

legend("bottomright", legend=c("Observed data", "Random data"), col=c("blue", "red"), lty=1, cex=0.8)

*# Define the number of iterations*

n_iterations <- 10000

*# Create an empty vector to store the R2 values*

p_value <- numeric(n_iterations) D <- numeric(n_iterations)

ktest_values <- as.data.frame(cbind(D, p_value))

*# Looping the Kolmogorov test*

dat_wf <- dat %>% filter(season_sex == "dry-female")

**for** (i **in** 1:n_iterations) {

*# Get subsample*

obs_dat <- sample(dat_wf$log_slope, size = 100, replace = F)

rand_dat <- sample(slope_rand$log_slope, size = 100, replace = F)

*# Kolmogorof test*

k_test <- ks.test((obs_dat), (rand_dat))

*# Calculate the values and store it in the vector*

ktest_values[i,1] <- k_test$statistic ktest_values[i,2] <- k_test$p.value

}

*# Test values*

mean(ktest_values$p_value) sd(ktest_values$p_value)

mean(ktest_values$D) sd(ktest_values$D)

*# Cows, wet season*

obs_dat <- dat %>% filter(season_sex == "wet-female") obs_dat <- obs_dat$log_slope

rand_dat <- slope_rand$log_slope

ks.test(obs_dat, rand_dat)

*# Visualization*

plot(ecdf((obs_dat)),

xlim = range(c((obs_dat), (rand_dat))), col = "blue",

xlab="slope (log10(º))",ylab="cumulative percentage", main="Cows, wet season", cex.main=

1)

plot(ecdf((rand_dat)), add = TRUE,

lty = "dashed",

col = "red")

legend("bottomright", legend=c("Observed data", "Random data"), col=c("blue", "red"), lty=1, cex=0.8)

*# Define the number of iterations*

n_iterations <- 10000

*# Create an empty vector to store the R2 values*

p_value <- numeric(n_iterations) D <- numeric(n_iterations)

ktest_values <- as.data.frame(cbind(D, p_value))

*# Looping the Kolmogorov test*

dat_wf <- dat %>% filter(season_sex == "wet-female")

**for** (i **in** 1:n_iterations) {

*# Get subsample*

obs_dat <- sample(dat_wf$log_slope, size = 100, replace = F)

rand_dat <- sample(slope_rand$log_slope, size = 100, replace = F)

*# Kolmogorof test*

k_test <- ks.test((obs_dat), (rand_dat))

*# Calculate the values and store it in the vector*

ktest_values[i,1] <- k_test$statistic ktest_values[i,2] <- k_test$p.value

}

*# Test values*

mean(ktest_values$p_value) sd(ktest_values$p_value)

mean(ktest_values$D) sd(ktest_values$D)

*# Bulls, dry season*

obs_dat <- dat %>% filter(season_sex == "dry-male") obs_dat <- obs_dat$log_slope

rand_dat <- slope_rand$log_slope

ks.test(obs_dat, rand_dat)

*# Visualization*

plot(ecdf((obs_dat)),

xlim = range(c((obs_dat), (rand_dat))), col = "blue",

xlab="slope (log10(º))",ylab="cumulative percentage", main="Bulls, dry season", cex.main

=1)

plot(ecdf((rand_dat)), add = TRUE,

lty = "dashed",

col = "red")

legend("bottomright", legend=c("Observed data", "Random data"), col=c("blue", "red"), lty=1, cex=0.8)

*# Define the number of iterations*

n_iterations <- 10000

*# Create an empty vector to store the R2 values*

p_value <- numeric(n_iterations) D <- numeric(n_iterations)

ktest_values <- as.data.frame(cbind(D, p_value))

*# Looping the Kolmogorov test*

dat_wf <- dat %>% filter(season_sex == "dry-male")

**for** (i **in** 1:n_iterations) {

*# Get subsample*

obs_dat <- sample(dat_wf$log_slope, size = 100, replace = F)

rand_dat <- sample(slope_rand$log_slope, size = 100, replace = F)

*# Kolmogorof test*

k_test <- ks.test((obs_dat), (rand_dat))

*# Calculate the values and store it in the vector*

ktest_values[i,1] <- k_test$statistic ktest_values[i,2] <- k_test$p.value

}

*# Test Values*

mean(ktest_values$p_value) sd(ktest_values$p_value)

mean(ktest_values$D) sd(ktest_values$D)

*# Bulls, wet season*

obs_dat <- dat %>% filter(season_sex == "wet-male") obs_dat <- obs_dat$log_slope

rand_dat <- slope_rand$log_slope

ks.test(obs_dat, rand_dat)

*# Visualization*

plot(ecdf((obs_dat)),

xlim = range(c((obs_dat), (rand_dat))), col = "blue",

xlab="slope (log10(º))",ylab="cumulative percentage", main="Bulls, wet season", cex.main

=1)

plot(ecdf((rand_dat)), add = TRUE,

lty = "dashed",

col = "red")

legend("bottomright", legend=c("Observed data", "Random data"), col=c("blue", "red"), lty=1, cex=0.8)

*# Define the number of iterations*

n_iterations <- 10000

*# Create an empty vector to store the R2 values*

p_value <- numeric(n_iterations) D <- numeric(n_iterations)

ktest_values <- as.data.frame(cbind(D, p_value))

*# Looping the Kolmogorov test*

dat_wf <- dat %>% filter(season_sex == "wet-male")

**for** (i **in** 1:n_iterations) {

*# Get subsample*

obs_dat <- sample(dat_wf$log_slope, size = 100, replace = F)

rand_dat <- sample(slope_rand$log_slope, size = 100, replace = F)

*# Kolmogorof test*

k_test <- ks.test((obs_dat), (rand_dat))

*# Calculate the values and store it in the vector*

ktest_values[i,1] <- k_test$statistic ktest_values[i,2] <- k_test$p.value

}

*# Test values*

mean(ktest_values$p_value) sd(ktest_values$p_value)

mean(ktest_values$D) sd(ktest_values$D)

#### Temperature

*# Load data*

dat <- read_csv("Data/All_env_vars_tot_dat_with_temporal_all.csv")

rand_temp <- read_csv("Data/Random generated points/temp_rand_point.csv") season <- read_csv("Data/season_per_day.csv")

*# Get season in random data*

season <- season %>%

mutate(date = format(date, "%d-%m-%Y"))

rand_temp <- rand_temp %>%

dplyr::select(fixtime, LST_Day_1km) %>%

separate(fixtime, c("date", "time"), sep = " ") %>% separate(date, c("year", "month", "day"), sep="-")

rand_temp$day <- as.integer(rand_temp$day)

rand_temp$month <- as.integer(rand_temp$month)

rand_temp <- rand_temp %>% mutate(zero = "0",

day2 = ifelse(day<10, paste(zero, day, sep = ""), day),

month2 = ifelse(month<10, paste(zero, month, sep = ""), month), date = paste(day2, month2, year, sep = "-")) %>%

left_join(season, by= "date") %>%

mutate(id = paste(date, time, LST_Day_1km))

rand_temp <- rand_temp[!duplicated(rand_temp$id), ] temp_rand <- na.omit(rand_temp)

*# Plot*

ggplot() +

geom_density(data=temp_rand%>% filter(season == "dry"), aes(x=(LST_Day_1km*0.02-273)),

fill = "lightgray", alpha=0.25, linetype=2, linewidth=1, color="#600000") + geom_density(data=temp_rand%>% filter(season == "wet"), aes(x=(LST_Day_1km*0.02-273)),

fill = "lightgray", alpha=0.25, linetype=2, linewidth=1, color="darkblue") +

geom_density(data=dat %>% filter(season_sex == "dry-female"), aes(x=LST_Day_1km), fill = "lightgray", alpha=0.15, linewidth=1, color="#F2B701") +

geom_density(data=dat %>% filter(season_sex == "wet-female"), aes(x=LST_Day_1km), fill = "lightgray", alpha=0.15, linewidth=1, color="#009988") +

geom_density(data=dat %>% filter(season_sex == "dry-male"), aes(x=LST_Day_1km),

fill = "lightgray", alpha=0.15, linewidth=1, color="#E65518") +

geom_density(data=dat %>% filter(season_sex == "wet-male"), aes(x=LST_Day_1km), fill = "lightgray", alpha=0.15, linewidth=1, color="#88CCEE") +

scale_y_continuous("density") +

labs(x = "temperature (°C)") +

theme(axis.title.x = element_text(size = 16), axis.text.x = element_text(size = 14), axis.title.y = element_text(size = 16), axis.text.y = element_text(size = 14),

axis.line.x.bottom = element_line(colour = "black"), axis.line.y.left = element_line(colour = "black"),

axis.line.y.right = element_line(colour = "black"), panel.background = element_rect(fill = "white"),

panel.grid.major = element_line(color = "grey92"), panel.grid.minor = element_line(color = "grey92"))

Kolmogorov test

dat <- dat %>%

rename(temp = LST_Day_1km) %>% drop_na(temp)

temp_rand <- temp_rand %>% mutate(temp = LST_Day_1km*0.02-273)

*# Cows, dry season*

obs_dat <- dat %>% filter(season_sex == "dry-female") obs_dat <- obs_dat$temp

rand_dat <- temp_rand %>% filter(season == "dry")

rand_dat <- rand_dat$temp ks.test(obs_dat, rand_dat)

*# Visualization*

plot(ecdf((obs_dat)),

xlim = range(c((obs_dat), (rand_dat))), col = "blue",

xlab="temperature (°C)",ylab="cumulative percentage", main="Cows, dry season", cex.main=

1)

plot(ecdf((rand_dat)), add = TRUE,

lty = "dashed", col = "red")

legend("bottomright", legend=c("Observed data", "Random data"),

col=c("blue", "red"), lty=1, cex=0.8)

*# Define the number of iterations*

n_iterations <- 10000

*# Create an empty vector to store the R2 values*

p_value <- numeric(n_iterations) D <- numeric(n_iterations)

ktest_values <- as.data.frame(cbind(D, p_value))

*# Looping the Kolmogorov test*

dat_wf <- dat %>% filter(season_sex == "dry-female") rand_dry <- temp_rand %>% filter(season == "dry")

**for** (i **in** 1:n_iterations) {

*# Get subsample*

obs_dat <- sample(dat_wf$temp, size = 100, replace = F)

rand_dat <- sample(rand_dry$temp, size = 100, replace = F)

*# Kolmogorof test*

k_test <- ks.test((obs_dat), (rand_dat))

*# Calculate the values and store it in the vector*

ktest_values[i,1] <- k_test$statistic ktest_values[i,2] <- k_test$p.value

}

*# Test values*

mean(ktest_values$p_value) sd(ktest_values$p_value)

mean(ktest_values$D)

sd(ktest_values$D)

*# Cows, wet season*

obs_dat <- dat %>% filter(season_sex == "wet-female") obs_dat <- obs_dat$temp

rand_dat <- temp_rand %>% filter(season == "wet")

rand_dat <- rand_dat$temp ks.test(obs_dat, rand_dat)

*# Visualization*

plot(ecdf((obs_dat)),

xlim = range(c((obs_dat), (rand_dat))), col = "blue",

xlab="temperature (°C)",ylab="cumulative percentage", main="Cows, wet season", cex.main=

1)

plot(ecdf((rand_dat)), add = TRUE,

lty = "dashed", col = "red")

legend("bottomright", legend=c("Observed data", "Random data"),

col=c("blue", "red"), lty=1, cex=0.8)

*# Define the number of iterations*

n_iterations <- 10000

*# Create an empty vector to store the R2 values*

p_value <- numeric(n_iterations) D <- numeric(n_iterations)

ktest_values <- as.data.frame(cbind(D, p_value))

*# Looping the Kolmogorov test*

dat_wf <- dat %>% filter(season_sex == "wet-female") rand_dry <- temp_rand %>% filter(season == "wet")

**for** (i **in** 1:n_iterations) {

*# Get subsample*

obs_dat <- sample(dat_wf$temp, size = 100, replace = F)

rand_dat <- sample(rand_dry$temp, size = 100, replace = F)

*# Kolmogorof test*

k_test <- ks.test((obs_dat), (rand_dat))

*# Calculate the values and store it in the vector*

ktest_values[i,1] <- k_test$statistic ktest_values[i,2] <- k_test$p.value

}

*# Test values*

mean(ktest_values$p_value) sd(ktest_values$p_value)

mean(ktest_values$D)

sd(ktest_values$D)

*# Bulls, dry season*

obs_dat <- dat %>% filter(season_sex == "dry-male") obs_dat <- obs_dat$temp

rand_dat <- temp_rand %>% filter(season == "dry")

rand_dat <- rand_dat$temp ks.test(obs_dat, rand_dat)

*# Visualization*

plot(ecdf((obs_dat)),

xlim = range(c((obs_dat), (rand_dat))), col = "blue",

xlab="temperature (°C)",ylab="cumulative percentage", main="Bulls, dry season", cex.main

=1)

plot(ecdf((rand_dat)), add = TRUE,

lty = "dashed", col = "red")

legend("bottomright", legend=c("Observed data", "Random data"),

col=c("blue", "red"), lty=1, cex=0.8)

*# Define the number of iterations*

n_iterations <- 10000

*# Create an empty vector to store the R2 values*

p_value <- numeric(n_iterations) D <- numeric(n_iterations)

ktest_values <- as.data.frame(cbind(D, p_value))

*# Looping the Kolmogorov test*

dat_wf <- dat %>% filter(season_sex == "dry-male") rand_dry <- temp_rand %>% filter(season == "dry")

**for** (i **in** 1:n_iterations) {

*# Get subsample*

obs_dat <- sample(dat_wf$temp, size = 100, replace = F)

rand_dat <- sample(rand_dry$temp, size = 100, replace = F)

*# Kolmogorof test*

k_test <- ks.test((obs_dat), (rand_dat))

*# Calculate the values and store it in the vector*

ktest_values[i,1] <- k_test$statistic ktest_values[i,2] <- k_test$p.value

}

*# Test values*

mean(ktest_values$p_value) sd(ktest_values$p_value)

mean(ktest_values$D)

sd(ktest_values$D)

*# Bulls, wet season*

obs_dat <- dat %>% filter(season_sex == "wet-male") obs_dat <- obs_dat$temp

rand_dat <- temp_rand %>% filter(season == "wet")

rand_dat <- rand_dat$temp ks.test(obs_dat, rand_dat)

*# Visualization*

plot(ecdf((obs_dat)),

xlim = range(c((obs_dat), (rand_dat))), col = "blue",

xlab="temperature (°C)",ylab="cumulative percentage", main="Bulls, wet season", cex.main

=1)

plot(ecdf((rand_dat)), add = TRUE,

lty = "dashed", col = "red")

legend("bottomright", legend=c("Observed data", "Random data"),

col=c("blue", "red"), lty=1, cex=0.8)

*# Define the number of iterations*

n_iterations <- 10000

*# Create an empty vector to store the R2 values*

p_value <- numeric(n_iterations) D <- numeric(n_iterations)

ktest_values <- as.data.frame(cbind(D, p_value))

*# Looping the Kolmogorov test*

dat_wf <- dat %>% filter(season_sex == "wet-male") rand_dry <- temp_rand %>% filter(season == "wet")

**for** (i **in** 1:n_iterations) {

*# Get subsample*

obs_dat <- sample(dat_wf$temp, size = 100, replace = F)

rand_dat <- sample(rand_dry$temp, size = 100, replace = F)

*# Kolmogorof test*

k_test <- ks.test((obs_dat), (rand_dat))

*# Calculate the values and store it in the vector*

ktest_values[i,1] <- k_test$statistic ktest_values[i,2] <- k_test$p.value

}

*# Test values*

mean(ktest_values$p_value) sd(ktest_values$p_value)

mean(ktest_values$D)

sd(ktest_values$D)

# Revisitation regressions

### Distribution of revisitation rate

dat <- read_csv("Data/All_env_vars_tot_dat_adjusted.csv")

resampled_data <- dat[sample(nrow(dat), size = nrow(dat)/(nrow(dat)/10000), replace = T), ]

*# What distribution fits the n-per-year*

fits <- list(nor = fitdistr(resampled_data$n_per_year, "normal"), wei = fitdistr(resampled_data$n_per_year, "weibull"), gam = fitdistr(resampled_data$n_per_year, "gamma"),

log = fitdistr(resampled_data$n_per_year, "log-normal"))

sapply(fits, **function**(i) i$loglik)

The log-normal distribution was the best fit, thus the regression models were made with log(n_per_year).

### Distance to water

*# Load data*

dat <- <- read_csv("Data/All_env_vars_tot_dat_adjusted.csv")

*# Get mean revisitation rate per bin # Cows, dry season*

dat_made <- data.frame()

**try** <- c(seq(min(dat$sqrt_dist_water), max(dat$sqrt_dist_water), by=0.1))

bins <- data.frame(sqrt_dist_water = c(seq(min(dat$sqrt_dist_water), max(dat$sqrt_dist_wate r), by=0.1)))

**for**(i **in** 1:length(**try**)) {

bin <- subset(dat%>%filter(season_sex=="dry-female"), sqrt_dist_water>**try**[i] & sqrt_dist_wa ter<**try**[i]+0.1)

mean_slope <- mean(bin$n_per_year)

dat_made[i,1] <- mean_slope

}

dat_made <- as.data.frame(dat_made) dat_made <- cbind(dat_made, bins)

dat_made_df <- dat_made %>% drop_na(V1)

*# Cows, wet season*

dat_made <- data.frame()

**try** <- c(seq(min(dat$sqrt_dist_water), max(dat$sqrt_dist_water), by=0.1))

bins <- data.frame(sqrt_dist_water = c(seq(min(dat$sqrt_dist_water), max(dat$sqrt_dist_wate r), by=0.1)))

**for**(i **in** 1:length(**try**)) {

bin <- subset(dat%>%filter(season_sex=="wet-female"), sqrt_dist_water>**try**[i] & sqrt_dist_wa ter<**try**[i]+0.1)

mean_slope <- mean(bin$n_per_year)

dat_made[i,1] <- mean_slope

}

dat_made <- as.data.frame(dat_made) dat_made <- cbind(dat_made, bins)

dat_made_wf <- dat_made %>% drop_na(V1)

*# Bulls, dry season*

dat_made <- data.frame()

**try** <- c(seq(min(dat$sqrt_dist_water), max(dat$sqrt_dist_water), by=0.1))

bins <- data.frame(sqrt_dist_water = c(seq(min(dat$sqrt_dist_water), max(dat$sqrt_dist_wate r), by=0.1)))

**for**(i **in** 1:length(**try**)) {

bin <- subset(dat%>%filter(season_sex=="dry-male"), sqrt_dist_water>**try**[i] & sqrt_dist_wate r<**try**[i]+0.1)

mean_slope <- mean(bin$n_per_year)

dat_made[i,1] <- mean_slope

}

dat_made <- as.data.frame(dat_made) dat_made <- cbind(dat_made, bins)

dat_made_dm <- dat_made %>% drop_na(V1)

*# Bulls, wet season*

dat_made <- data.frame()

**try** <- c(seq(min(dat$sqrt_dist_water), max(dat$sqrt_dist_water), by=0.1))

bins <- data.frame(sqrt_dist_water = c(seq(min(dat$sqrt_dist_water), max(dat$sqrt_dist_wate r), by=0.1)))

**for**(i **in** 1:length(**try**)) {

bin <- subset(dat%>%filter(season_sex=="wet-male"), sqrt_dist_water>**try**[i] & sqrt_dist_wate r<**try**[i]+0.1)

mean_slope <- mean(bin$n_per_year)

dat_made[i,1] <- mean_slope

}

dat_made <- as.data.frame(dat_made) dat_made <- cbind(dat_made, bins)

dat_made_wm <- dat_made %>% drop_na(V1)

*# Join data*

dat_made_wf <- dat_made_wf %>%

mutate(Group = "Cows, wet season") dat_made_df <- dat_made_df %>%

mutate(Group = "Cows, dry season") dat_made_wm <- dat_made_wm %>%

mutate(Group = "Bulls, wet season")

dat_made_dm <- dat_made_dm %>%

mutate(Group = "Bulls, dry season")

sum_water <- rbind(dat_made_wf, dat_made_df, dat_made_wm, dat_made_dm)

*# Plot*

ggplot() +

geom_point(data = sum_water %>% filter(Group == "Bulls, wet season"),

aes(x = sqrt_dist_water^2, y = V1), color="#88CCEE", shape=24, fill = "#88CCEE", alpha = 0.9, size=2) +

geom_point(data = sum_water %>% filter(Group == "Bulls, dry season"),

aes(x = sqrt_dist_water^2, y = V1), color="#E65518", shape=24, fill = "#E65518", alpha = 0.9, size=2) +

geom_point(data = sum_water %>% filter(Group == "Cows, wet season"),

aes(x = sqrt_dist_water^2, y = V1), color="#009988", shape=21, fill = "#009988", alpha = 0.9, size=2) +

geom_point(data = sum_water %>% filter(Group == "Cows, dry season"),

aes(x = sqrt_dist_water^2, y = V1), color="#F2B701", shape=21, fill = "#F2B701", alpha = 0.9, size=2) +

scale_x_sqrt()+

labs(x = "distance to water (m)", y="revisitation rate") + theme(axis.title.x = element_text(size = 16),

axis.text.x = element_text(size = 14),

axis.title.y = element_text(size = 16), axis.text.y = element_text(size = 14),

axis.line.x.bottom = element_line(colour = "black"), axis.line.y.left = element_line(colour = "black"),

axis.line.y.right = element_line(colour = "black"), panel.background = element_rect(fill = "white"),

panel.grid.major = element_line(color = "grey92"), panel.grid.minor = element_line(color = "grey92"))

*# Regression models*

sum_water <- sum_water %>% mutate(sqrt_dist_water2 = sqrt_dist_water^2)

*## Cows, dry season*

water_mod <- sum_water %>% filter(Group == "Cows, dry season") %>% mutate(n_year_log = log(V1))

my_recipe <- recipe(n_year_log ~ sqrt_dist_water + sqrt_dist_water2, data = water_mod) my_recipe <- my_recipe %>% step_scale(all_numeric_predictors())

prep_recipe <- prep(my_recipe)

scaled_data <- bake(prep_recipe, new_data = water_mod)

water_model <- lm(n_year_log ~ sqrt_dist_water + sqrt_dist_water2, data = scaled_data) summary(water_model)

*## Cows, wet season*

water_mod <- sum_water %>% filter(Group == "Cows, wet season") %>% mutate(n_year_log = log(V1))

my_recipe <- recipe(n_year_log ~ sqrt_dist_water + sqrt_dist_water2, data = water_mod) my_recipe <- my_recipe %>% step_scale(all_numeric_predictors())

prep_recipe <- prep(my_recipe)

scaled_data <- bake(prep_recipe, new_data = water_mod)

water_model <- lm(n_year_log ~ sqrt_dist_water + sqrt_dist_water2, data = scaled_data) summary(water_model)

*## Bulls, dry season*

water_mod <- sum_water %>% filter(Group == "Bulls, dry season") %>% mutate(n_year_log = log(V1))

my_recipe <- recipe(n_year_log ~ sqrt_dist_water + sqrt_dist_water2, data = water_mod) my_recipe <- my_recipe %>% step_scale(all_numeric_predictors())

prep_recipe <- prep(my_recipe)

scaled_data <- bake(prep_recipe, new_data = water_mod)

water_model <- lm(n_year_log ~ sqrt_dist_water + sqrt_dist_water2, data = scaled_data) summary(water_model)

*## Bulls, wet season*

water_mod <- sum_water %>% filter(Group == "Bulls, wet season") %>% mutate(n_year_log = log(V1))

my_recipe <- recipe(n_year_log ~ sqrt_dist_water + sqrt_dist_water2, data = water_mod) my_recipe <- my_recipe %>% step_scale(all_numeric_predictors())

prep_recipe <- prep(my_recipe)

scaled_data <- bake(prep_recipe, new_data = water_mod)

water_model <- lm(n_year_log ~ sqrt_dist_water + sqrt_dist_water2, data = scaled_data) summary(water_model)

### EVI

*# Load data*

dat_temp <- read_csv("Data/All_env_vars_tot_dat_with_temporal.csv")

*# Get mean revisitation rate per bin*

dat_temp <- dat_temp %>% drop_na(EVI)

*# Cows, dry season*

dat_made <- data.frame()

**try** <- c(seq(min(dat_temp$EVI), max(dat_temp$EVI), by=9))

bins <- data.frame(EVI = c(seq(min(dat_temp$EVI), max(dat_temp$EVI), by=9)))

**for**(i **in** 1:length(**try**)) {

bin <- subset(dat_temp%>%filter(season_sex=="dry-female"), EVI>**try**[i] & EVI<**try**[i]+9) mean_slope <- mean(bin$n_per_year)

dat_made[i,1] <- mean_slope

}

dat_made <- as.data.frame(dat_made) dat_made <- cbind(dat_made, bins)

dat_made_df <- dat_made %>% drop_na(V1)

*# Cows, wet season*

dat_made <- data.frame()

**try** <- c(seq(min(dat_temp$EVI), max(dat_temp$EVI), by=9))

bins <- data.frame(EVI = c(seq(min(dat_temp$EVI), max(dat_temp$EVI), by=9)))

**for**(i **in** 1:length(**try**)) {

bin <- subset(dat_temp%>%filter(season_sex=="wet-female"), EVI>**try**[i] & EVI<**try**[i]+9) mean_slope <- mean(bin$n_per_year)

dat_made[i,1] <- mean_slope

}

dat_made <- as.data.frame(dat_made) dat_made <- cbind(dat_made, bins)

dat_made_wf <- dat_made %>% drop_na(V1)

*# Bulls, dry season*

dat_made <- data.frame()

**try** <- c(seq(min(dat_temp$EVI), max(dat_temp$EVI), by=9))

bins <- data.frame(EVI = c(seq(min(dat_temp$EVI), max(dat_temp$EVI), by=9)))

**for**(i **in** 1:length(**try**)) {

bin <- subset(dat_temp%>%filter(season_sex=="dry-male"), EVI>**try**[i] & EVI<**try**[i]+9) mean_slope <- mean(bin$n_per_year)

dat_made[i,1] <- mean_slope

}

dat_made <- as.data.frame(dat_made) dat_made <- cbind(dat_made, bins)

dat_made_dm <- dat_made %>% drop_na(V1)

*# Bulls, wet season*

dat_made <- data.frame()

**try** <- c(seq(min(dat_temp$EVI), max(dat_temp$EVI), by=9))

bins <- data.frame(EVI = c(seq(min(dat_temp$EVI), max(dat_temp$EVI), by=9)))

**for**(i **in** 1:length(**try**)) {

bin <- subset(dat_temp%>%filter(season_sex=="wet-male"), EVI>**try**[i] & EVI<**try**[i]+9) mean_slope <- mean(bin$n_per_year)

dat_made[i,1] <- mean_slope

}

dat_made <- as.data.frame(dat_made) dat_made <- cbind(dat_made, bins)

dat_made_wm <- dat_made %>% drop_na(V1)

*# Join data*

dat_made_wf <- dat_made_wf %>%

mutate(Group = "Cows, wet season") dat_made_df <- dat_made_df %>%

mutate(Group = "Cows, dry season") dat_made_wm <- dat_made_wm %>%

mutate(Group = "Bulls, wet season")

dat_made_dm <- dat_made_dm %>%

mutate(Group = "Bulls, dry season")

sum_evi <- rbind(dat_made_wf, dat_made_df, dat_made_wm, dat_made_dm)

*# Plot*

ggplot() +

geom_point(data = sum_evi %>% filter(Group == "Bulls, wet season", V1 < 20), aes(x = EVI*0.0001, y = V1), color="#88CCEE", shape=24,

fill = "#88CCEE", alpha = 0.9, size=2) +

geom_point(data = sum_evi %>% filter(Group == "Bulls, dry season", V1 < 20), aes(x = EVI*0.0001, y = V1), color="#E65518", shape=24,

fill = "#E65518", alpha = 0.9, size=2) +

geom_point(data = sum_evi %>% filter(Group == "Cows, wet season", V1 < 20), aes(x = EVI*0.0001, y = V1), color="#009988", shape=21,

fill = "#009988", alpha = 0.9, size=2) +

geom_point(data = sum_evi %>% filter(Group == "Cows, dry season", V1 < 20), aes(x = EVI*0.0001, y = V1), color="#F2B701", shape=21,

fill = "#F2B701", alpha = 0.9, size=2) +

labs(x = "EVI", y="revisitation rate") +

theme(axis.title.x = element_text(size = 16), axis.text.x = element_text(size = 14), axis.title.y = element_text(size = 16), axis.text.y = element_text(size = 14),

axis.line.x.bottom = element_line(colour = "black"), axis.line.y.left = element_line(colour = "black"),

axis.line.y.right = element_line(colour = "black"), panel.background = element_rect(fill = "white"),

panel.grid.major = element_line(color = "grey92"), panel.grid.minor = element_line(color = "grey92"))

*# Regression models*

sum_evi <- sum_evi %>% mutate(EVI2 = EVI^2)

*## Cows, dry season*

evi_mod <- sum_evi %>% filter(Group == "Cows, dry season") %>% mutate(n_year_log = log(V1))

my_recipe <- recipe(n_year_log ~ EVI + EVI2, data = evi_mod)

my_recipe <- my_recipe %>% step_scale(all_numeric_predictors()) prep_recipe <- prep(my_recipe)

scaled_data <- bake(prep_recipe, new_data = evi_mod)

evi_model <- lm(n_year_log ~ EVI + EVI2, data = scaled_data) summary(evi_model)

*## Cows, wet season*

evi_mod <- sum_evi %>% filter(Group == "Cows, wet season") %>% mutate(n_year_log = log(V1))

my_recipe <- recipe(n_year_log ~ EVI + EVI2, data = evi_mod)

my_recipe <- my_recipe %>% step_scale(all_numeric_predictors()) prep_recipe <- prep(my_recipe)

scaled_data <- bake(prep_recipe, new_data = evi_mod)

evi_model <- lm(n_year_log ~ EVI + EVI2, data = scaled_data) summary(evi_model)

*## Bulls, dry season*

evi_mod <- sum_evi %>% filter(Group == "Bulls, dry season") %>% mutate(n_year_log = log(V1))

my_recipe <- recipe(n_year_log ~ EVI + EVI2, data = evi_mod)

my_recipe <- my_recipe %>% step_scale(all_numeric_predictors()) prep_recipe <- prep(my_recipe)

scaled_data <- bake(prep_recipe, new_data = evi_mod)

evi_model <- lm(n_year_log ~ EVI + EVI2, data = scaled_data) summary(evi_model)

*## Bulls, wet season*

evi_mod <- sum_evi %>% filter(Group == "Bulls, wet season") %>% mutate(n_year_log = log(V1))

my_recipe <- recipe(n_year_log ~ EVI + EVI2, data = evi_mod)

my_recipe <- my_recipe %>% step_scale(all_numeric_predictors()) prep_recipe <- prep(my_recipe)

scaled_data <- bake(prep_recipe, new_data = evi_mod)

evi_model <- lm(n_year_log ~ EVI + EVI2, data = scaled_data) summary(evi_model)

### Phosphor

*# Load data*

dat <- <- read_csv("Data/All_env_vars_tot_dat_adjusted.csv")

*# Get mean revisitation rate per bin # Cows, dry season*

dat_made <- data.frame()

**try** <- c(seq(min(dat$phosphor), max(dat$phosphor), by=0.007))

bins <- data.frame(phosphor = c(seq(min(dat$phosphor), max(dat$phosphor), by=0.007)))

**for**(i **in** 1:length(**try**)) {

bin <- subset(dat%>%filter(season_sex=="dry-female"), phosphor>**try**[i] & phosphor<**try**[i]+0.0 07)

mean_slope <- mean(bin$n_per_year)

dat_made[i,1] <- mean_slope

}

dat_made <- as.data.frame(dat_made) dat_made <- cbind(dat_made, bins)

dat_made_df <- dat_made %>% drop_na(V1)

*# Cows, wet season*

dat_made <- data.frame()

**try** <- c(seq(min(dat$phosphor), max(dat$phosphor), by=0.007))

bins <- data.frame(phosphor = c(seq(min(dat$phosphor), max(dat$phosphor), by=0.007)))

**for**(i **in** 1:length(**try**)) {

bin <- subset(dat%>%filter(season_sex=="wet-female"), phosphor>**try**[i] & phosphor<**try**[i]+0.0 07)

mean_slope <- mean(bin$n_per_year)

dat_made[i,1] <- mean_slope

}

dat_made <- as.data.frame(dat_made) dat_made <- cbind(dat_made, bins)

dat_made_wf <- dat_made %>% drop_na(V1)

*# Bulls, dry season*

dat_made <- data.frame()

**try** <- c(seq(min(dat$phosphor), max(dat$phosphor), by=0.007))

bins <- data.frame(phosphor = c(seq(min(dat$phosphor), max(dat$phosphor), by=0.007)))

**for**(i **in** 1:length(**try**)) {

bin <- subset(dat%>%filter(season_sex=="dry-male"), phosphor>**try**[i] & phosphor<**try**[i]+0.00 7)

mean_slope <- mean(bin$n_per_year)

dat_made[i,1] <- mean_slope

}

dat_made <- as.data.frame(dat_made) dat_made <- cbind(dat_made, bins)

dat_made_dm <- dat_made %>% drop_na(V1)

*# Bulls, wet season*

dat_made <- data.frame()

**try** <- c(seq(min(dat$phosphor), max(dat$phosphor), by=0.007))

bins <- data.frame(phosphor = c(seq(min(dat$phosphor), max(dat$phosphor), by=0.007)))

**for**(i **in** 1:length(**try**)) {

bin <- subset(dat%>%filter(season_sex=="wet-male"), phosphor>**try**[i] & phosphor<**try**[i]+0.00 7)

mean_slope <- mean(bin$n_per_year)

dat_made[i,1] <- mean_slope

}

dat_made <- as.data.frame(dat_made) dat_made <- cbind(dat_made, bins)

dat_made_wm <- dat_made %>% drop_na(V1)

*# Join data*

dat_made_wf <- dat_made_wf %>%

mutate(Group = "Cows, wet season") dat_made_df <- dat_made_df %>%

mutate(Group = "Cows, dry season") dat_made_wm <- dat_made_wm %>%

mutate(Group = "Bulls, wet season") dat_made_dm <- dat_made_dm %>%

mutate(Group = "Bulls, dry season")

sum_phos <- rbind(dat_made_wf, dat_made_df, dat_made_wm, dat_made_dm)

*# Plot*

ggplot() +

geom_point(data = sum_phos %>% filter(Group == "Bulls, wet season"), aes(x = phosphor, y = V1), color="#88CCEE", shape=24,

fill = "#88CCEE", alpha = 0.9, size=2) +

geom_point(data = sum_phos %>% filter(Group == "Bulls, dry season"), aes(x = phosphor, y = V1), color="#E65518", shape=24,

fill = "#E65518", alpha = 0.9, size=2) +

geom_point(data = sum_phos %>% filter(Group == "Cows, wet season"), aes(x = phosphor, y = V1), color="#009988", shape=21,

fill = "#009988", alpha = 0.9, size=2) +

geom_point(data = sum_phos %>% filter(Group == "Cows, dry season"), aes(x = phosphor, y = V1), color="#F2B701", shape=21,

fill = "#F2B701", alpha = 0.9, size=2) + labs(x = "phosphorus (ppm)", y="revisitation rate") + theme( axis.title.x = element_text(size = 16),

axis.text.x = element_text(size = 14), axis.title.y = element_text(size = 16), axis.text.y = element_text(size = 14),

axis.line.x.bottom = element_line(colour = "black"), axis.line.y.left = element_line(colour = "black"),

axis.line.y.right = element_line(colour = "black"),

panel.background = element_rect(fill = "white"), panel.grid.major = element_line(color = "grey92"), panel.grid.minor = element_line(color = "grey92"))

*# Regression models*

sum_phos <- sum_phos %>% mutate(phosphor2 = phosphor^2)

*## Cows, dry season*

phos_mod <- sum_phos %>% filter(Group == "Cows, dry season") %>% mutate(n_year_log = log(V1))

my_recipe <- recipe(n_year_log ~ phosphor + phosphor2, data = phos_mod) my_recipe <- my_recipe %>% step_scale(all_numeric_predictors())

prep_recipe <- prep(my_recipe)

scaled_data <- bake(prep_recipe, new_data = phos_mod)

phos_model <- lm(n_year_log ~ phosphor + phosphor2, data = scaled_data) summary(phos_model)

*## Cows, wet season*

phos_mod <- sum_phos %>% filter(Group == "Cows, wet season") %>% mutate(n_year_log = log(V1))

my_recipe <- recipe(n_year_log ~ phosphor + phosphor2, data = phos_mod) my_recipe <- my_recipe %>% step_scale(all_numeric_predictors())

prep_recipe <- prep(my_recipe)

scaled_data <- bake(prep_recipe, new_data = phos_mod)

phos_model <- lm(n_year_log ~ phosphor + phosphor2, data = scaled_data) summary(phos_model)

*## Bulls, dry season*

phos_mod <- sum_phos %>% filter(Group == "Bulls, dry season") %>% mutate(n_year_log = log(V1))

my_recipe <- recipe(n_year_log ~ phosphor + phosphor2, data = phos_mod) my_recipe <- my_recipe %>% step_scale(all_numeric_predictors())

prep_recipe <- prep(my_recipe)

scaled_data <- bake(prep_recipe, new_data = phos_mod)

phos_model <- lm(n_year_log ~ phosphor + phosphor2, data = scaled_data) summary(phos_model)

*## Bulls, wet season*

phos_mod <- sum_phos %>% filter(Group == "Bulls, wet season") %>% mutate(n_year_log = log(V1))

my_recipe <- recipe(n_year_log ~ phosphor + phosphor2, data = phos_mod) my_recipe <- my_recipe %>% step_scale(all_numeric_predictors())

prep_recipe <- prep(my_recipe)

scaled_data <- bake(prep_recipe, new_data = phos_mod)

phos_model <- lm(n_year_log ~ phosphor + phosphor2, data = scaled_data) summary(phos_model)

### Precipitation

*# Load data*

dat_temp <- read_csv("Data/All_env_vars_tot_dat_with_temporal.csv")

*# Get mean revisitation rate per bin*

dat_temp <- dat_temp %>% drop_na(log_precip)

*# Cows, dry season*

dat_made <- data.frame()

**try** <- c(seq(min(dat_temp$log_precip), max(dat_temp$log_precip), by=0.001))

bins <- data.frame(log_precip= c(seq(min(dat_temp$log_precip), max(dat_temp$log_precip), by= 0.001)))

**for**(i **in** 1:length(**try**)) {

bin <- subset(dat_temp%>%filter(season_sex=="dry-female"), log_precip>**try**[i] & log_precip<**t ry**[i]+0.001)

mean_slope <- mean(bin$n_per_year)

dat_made[i,1] <- mean_slope

}

dat_made <- as.data.frame(dat_made) dat_made <- cbind(dat_made, bins)

dat_made_df <- dat_made %>% drop_na(V1)

*# Cows, wet season*

dat_made <- data.frame()

**try** <- c(seq(min(dat_temp$log_precip), max(dat_temp$log_precip), by=0.001))

bins <- data.frame(log_precip= c(seq(min(dat_temp$log_precip), max(dat_temp$log_precip), by= 0.001)))

**for**(i **in** 1:length(**try**)) {

bin <- subset(dat_temp%>%filter(season_sex=="wet-female"), log_precip>**try**[i] & log_precip<**t ry**[i]+0.001)

mean_slope <- mean(bin$n_per_year)

dat_made[i,1] <- mean_slope

}

dat_made <- as.data.frame(dat_made) dat_made <- cbind(dat_made, bins)

dat_made_wf <- dat_made %>% drop_na(V1)

*# Bulls, dry season*

dat_made <- data.frame()

**try** <- c(seq(min(dat_temp$log_precip), max(dat_temp$log_precip), by=0.001))

bins <- data.frame(log_precip= c(seq(min(dat_temp$log_precip), max(dat_temp$log_precip), by= 0.001)))

**for**(i **in** 1:length(**try**)) {

bin <- subset(dat_temp%>%filter(season_sex=="dry-male"), log_precip>**try**[i] & log_precip<**try** [i]+0.001)

mean_slope <- mean(bin$n_per_year)

dat_made[i,1] <- mean_slope

}

dat_made <- as.data.frame(dat_made) dat_made <- cbind(dat_made, bins)

dat_made_dm <- dat_made %>% drop_na(V1)

*# Bulls, wet season*

dat_made <- data.frame()

**try** <- c(seq(min(dat_temp$log_precip), max(dat_temp$log_precip), by=0.001))

bins <- data.frame(log_precip= c(seq(min(dat_temp$log_precip), max(dat_temp$log_precip), by= 0.001)))

**for**(i **in** 1:length(**try**)) {

bin <- subset(dat_temp%>%filter(season_sex=="wet-male"), log_precip>**try**[i] & log_precip<**try** [i]+0.001)

mean_slope <- mean(bin$n_per_year)

dat_made[i,1] <- mean_slope

}

dat_made <- as.data.frame(dat_made) dat_made <- cbind(dat_made, bins)

dat_made_wm <- dat_made %>% drop_na(V1)

*# Join data*

dat_made_wf <- dat_made_wf %>%

mutate(Group = "Cows, wet season") dat_made_df <- dat_made_df %>%

mutate(Group = "Cows, dry season") dat_made_wm <- dat_made_wm %>%

mutate(Group = "Bulls, wet season") dat_made_dm <- dat_made_dm %>%

mutate(Group = "Bulls, dry season")

sum_precip <- rbind(dat_made_wf, dat_made_df, dat_made_wm, dat_made_dm)

*# Plot*

ggplot() +

geom_point(data = sum_prec %>% filter(Group == "Bulls, wet season", V1<17), aes(x = 10^log_precip, y = V1), color="#88CCEE", shape=24,

fill = "#88CCEE", alpha = 0.9, size=2) +

geom_point(data = sum_prec %>% filter(Group == "Bulls, dry season", V1<17), aes(x = 10^log_precip, y = V1), color="#E65518", shape=24,

fill = "#E65518", alpha = 0.9, size=2) +

geom_point(data = sum_prec %>% filter(Group == "Cows, wet season", V1<17), aes(x = 10^log_precip, y = V1), color="#009988", shape=21,

fill = "#009988", alpha = 0.9, size=2) +

geom_point(data = sum_prec %>% filter(Group == "Cows, dry season", V1<17), aes(x = 10^log_precip, y = V1), color="#F2B701", shape=21,

fill = "#F2B701", alpha = 0.9, size=2) + scale_x_log10() +

labs(x = "precipitation (mm/5 days)", y="revisitation rate") + theme(axis.title.x = element_text(size = 16),

axis.text.x = element_text(size = 14),

axis.title.y = element_text(size = 16), axis.text.y = element_text(size = 14),

axis.line.x.bottom = element_line(colour = "black"), axis.line.y.left = element_line(colour = "black"),

axis.line.y.right = element_line(colour = "black"), panel.background = element_rect(fill = "white"),

panel.grid.major = element_line(color = "grey92"), panel.grid.minor = element_line(color = "grey92"))

*# Regression models*

sum_prec <- sum_prec %>% mutate(log_precip2 = log_precip^2)

*## Cows, dry season*

prec_mod <- sum_prec %>% filter(Group == "Cows, dry season") %>% mutate(n_year_log = log(V1))

my_recipe <- recipe(n_year_log ~ log_precip + log_precip2, data = prec_mod) my_recipe <- my_recipe %>% step_scale(all_numeric_predictors())

prep_recipe <- prep(my_recipe)

scaled_data <- bake(prep_recipe, new_data = prec_mod)

prec_model <- lm(n_year_log ~ log_precip + log_precip2, data = scaled_data) summary(prec_model)

*## Cows, wet season*

prec_mod <- sum_prec %>% filter(Group == "Cows, wet season") %>% mutate(n_year_log = log(V1))

my_recipe <- recipe(n_year_log ~ log_precip + log_precip2, data = prec_mod) my_recipe <- my_recipe %>% step_scale(all_numeric_predictors())

prep_recipe <- prep(my_recipe)

scaled_data <- bake(prep_recipe, new_data = prec_mod)

prec_model <- lm(n_year_log ~ log_precip + log_precip2, data = scaled_data) summary(prec_model)

*## Bulls, dry season*

prec_mod <- sum_prec %>% filter(Group == "Bulls, dry season") %>% mutate(n_year_log = log(V1))

my_recipe <- recipe(n_year_log ~ log_precip + log_precip2, data = prec_mod) my_recipe <- my_recipe %>% step_scale(all_numeric_predictors())

prep_recipe <- prep(my_recipe)

scaled_data <- bake(prep_recipe, new_data = prec_mod)

prec_model <- lm(n_year_log ~ log_precip + log_precip2, data = scaled_data) summary(prec_model)

*## Bulls, wet season*

prec_mod <- sum_prec %>% filter(Group == "Bulls, wet season") %>% mutate(n_year_log = log(V1))

my_recipe <- recipe(n_year_log ~ log_precip + log_precip2, data = prec_mod) my_recipe <- my_recipe %>% step_scale(all_numeric_predictors())

prep_recipe <- prep(my_recipe)

scaled_data <- bake(prep_recipe, new_data = prec_mod)

prec_model <- lm(n_year_log ~ log_precip + log_precip2, data = scaled_data) summary(prec_model)

### Slope

*# Load data*

dat <- read_csv("Data/All_env_vars_tot_dat_adjusted.csv")

*# Get mean revisitation rate per bin # Cows, dry season*

dat_made <- data.frame()

**try** <- c(seq(min(dat$log_slope), max(dat$log_slope), by=0.001))

bins <- data.frame(log_slope = c(seq(min(dat$log_slope), max(dat$log_slope), by=0.001)))

**for**(i **in** 1:length(**try**)) {

bin <- subset(dat%>%filter(season_sex=="dry-female"), log_slope>**try**[i] & log_slope<**try**[i]+ 0.001)

mean_slope <- mean(bin$n_per_year)

dat_made[i,1] <- mean_slope

}

dat_made <- as.data.frame(dat_made) dat_made <- cbind(dat_made, bins)

dat_made_df <- dat_made %>% drop_na(V1)

*# Cows, wet season*

dat_made <- data.frame()

**try** <- c(seq(min(dat$log_slope), max(dat$log_slope), by=0.001))

bins <- data.frame(log_slope = c(seq(min(dat$log_slope), max(dat$log_slope), by=0.001)))

**for**(i **in** 1:length(**try**)) {

bin <- subset(dat%>%filter(season_sex=="wet-female"), log_slope>**try**[i] & log_slope<**try**[i]+ 0.001)

mean_slope <- mean(bin$n_per_year)

dat_made[i,1] <- mean_slope

}

dat_made <- as.data.frame(dat_made) dat_made <- cbind(dat_made, bins)

dat_made_wf <- dat_made %>% drop_na(V1)

*# Bulls, dry season*

dat_made <- data.frame()

**try** <- c(seq(min(dat$log_slope), max(dat$log_slope), by=0.001))

bins <- data.frame(log_slope = c(seq(min(dat$log_slope), max(dat$log_slope), by=0.001)))

**for**(i **in** 1:length(**try**)) {

bin <- subset(dat%>%filter(season_sex=="dry-male"), log_slope>**try**[i] & log_slope<**try**[i]+0.0 01)

mean_slope <- mean(bin$n_per_year)

dat_made[i,1] <- mean_slope

}

dat_made <- as.data.frame(dat_made) dat_made <- cbind(dat_made, bins)

dat_made_dm <- dat_made %>% drop_na(V1)

*# Bulls, wet season*

dat_made <- data.frame()

**try** <- c(seq(min(dat$log_slope), max(dat$log_slope), by=0.001))

bins <- data.frame(log_slope = c(seq(min(dat$log_slope), max(dat$log_slope), by=0.001)))

**for**(i **in** 1:length(**try**)) {

bin <- subset(dat%>%filter(season_sex=="wet-male"), log_slope>**try**[i] & log_slope<**try**[i]+0.0 01)

mean_slope <- mean(bin$n_per_year)

dat_made[i,1] <- mean_slope

}

dat_made <- as.data.frame(dat_made) dat_made <- cbind(dat_made, bins)

dat_made_wm <- dat_made %>% drop_na(V1)

*# Join data*

dat_made_wf <- dat_made_wf %>% mutate(slope = 10^log_slope,

Group = "Cows, wet season")

dat_made_df <- dat_made_df %>% mutate(slope = 10^log_slope,

Group = "Cows, dry season") dat_made_wm <- dat_made_wm %>%

mutate(slope = 10^log_slope,

Group = "Bulls, wet season") dat_made_dm <- dat_made_dm %>%

mutate(slope = 10^log_slope,

Group = "Bulls, dry season")

sum_slope <- rbind(dat_made_wf, dat_made_df, dat_made_wm, dat_made_dm)

*# Plot*

ggplot() +

geom_point(data = sum_slope %>% filter(Group == "Bulls, wet season"), aes(x = slope, y = V1), color="#88CCEE", shape=24,

fill = "#88CCEE", alpha = 0.9, size=2) +

geom_point(data = sum_slope %>% filter(Group == "Bulls, dry season"), aes(x = slope, y = V1), color="#E65518", shape=24,

fill = "#E65518", alpha = 0.9, size=2) +

geom_point(data = sum_slope %>% filter(Group == "Cows, wet season"), aes(x = slope, y = V1), color="#009988", shape=21,

fill = "#009988", alpha = 0.9, size=2) +

geom_point(data = sum_slope %>% filter(Group == "Cows, dry season"), aes(x = slope, y = V1), color="#F2B701", shape=21,

fill = "#F2B701", alpha = 0.9, size=2) + scale_x_log10() +

labs(x = "slope (degree)", y="revisitation rate") + theme(axis.title.x = element_text(size = 16),

axis.text.x = element_text(size = 14),

axis.title.y = element_text(size = 16), axis.text.y = element_text(size = 14),

axis.line.x.bottom = element_line(colour = "black"), axis.line.y.left = element_line(colour = "black"),

axis.line.y.right = element_line(colour = "black"), panel.background = element_rect(fill = "white"),

panel.grid.major = element_line(color = "grey92"), panel.grid.minor = element_line(color = "grey92"))

*# Regression models*

sum_slope <- sum_slope %>% mutate(log_slope2 = log_slope^2)

*## Cows, dry season*

slope_mod <- sum_slope %>% filter(Group == "Cows, dry season") %>% mutate(n_year_log = log(V1))

my_recipe <- recipe(n_year_log ~ log_slope + log_slope2, data = slope_mod) my_recipe <- my_recipe %>% step_scale(all_numeric_predictors())

prep_recipe <- prep(my_recipe)

scaled_data <- bake(prep_recipe, new_data = slope_mod)

slope_model <- lm(n_year_log ~ log_slope + log_slope2, data = scaled_data) summary(slope_model)

*## Cows, wet season*

slope_mod <- sum_slope %>% filter(Group == "Cows, wet season") %>% mutate(n_year_log = log(V1))

my_recipe <- recipe(n_year_log ~ log_slope + log_slope2, data = slope_mod) my_recipe <- my_recipe %>% step_scale(all_numeric_predictors())

prep_recipe <- prep(my_recipe)

scaled_data <- bake(prep_recipe, new_data = slope_mod)

slope_model <- lm(n_year_log ~ log_slope + log_slope2, data = scaled_data) summary(slope_model)

*## Bulls, dry season*

slope_mod <- sum_slope %>% filter(Group == "Bulls, dry season") %>% mutate(n_year_log = log(V1))

my_recipe <- recipe(n_year_log ~ log_slope + log_slope2, data = slope_mod) my_recipe <- my_recipe %>% step_scale(all_numeric_predictors())

prep_recipe <- prep(my_recipe)

scaled_data <- bake(prep_recipe, new_data = slope_mod)

slope_model <- lm(n_year_log ~ log_slope + log_slope2, data = scaled_data) summary(slope_model)

*## Bulls, wet season*

slope_mod <- sum_slope %>% filter(Group == "Bulls, wet season") %>% mutate(n_year_log = log(V1))

my_recipe <- recipe(n_year_log ~ log_slope + log_slope2, data = slope_mod) my_recipe <- my_recipe %>% step_scale(all_numeric_predictors())

prep_recipe <- prep(my_recipe)

scaled_data <- bake(prep_recipe, new_data = slope_mod)

slope_model <- lm(n_year_log ~ log_slope + log_slope2, data = scaled_data) summary(slope_model)

### Temperature

*# Load data*

dat_temp <- <- read_csv("Data/All_env_vars_tot_dat_with_temporal.csv")

*# Get mean revisitation rate per bin*

dat_temp <- dat_temp %>% drop_na(LST_Day_1km)

*# Cows, dry season*

dat_made <- data.frame()

**try** <- c(seq(min(dat_temp$LST_Day_1km), max(dat_temp$LST_Day_1km), by=0.05))

bins <- data.frame(LST_Day_1km = c(seq(min(dat_temp$LST_Day_1km), max(dat_temp$LST_Day_1km), by=0.05)))

**for**(i **in** 1:length(**try**)) {

bin <- subset(dat_temp%>%filter(season_sex=="dry-female"), LST_Day_1km>**try**[i] & LST_Day_1km

<**try**[i]+0.05)

mean_slope <- mean(bin$n_per_year)

dat_made[i,1] <- mean_slope

}

dat_made <- as.data.frame(dat_made) dat_made <- cbind(dat_made, bins)

dat_made_df <- dat_made %>% drop_na(V1)

*# Cows, wet season*

dat_made <- data.frame()

**try** <- c(seq(min(dat_temp$LST_Day_1km), max(dat_temp$LST_Day_1km), by=0.05))

bins <- data.frame(LST_Day_1km = c(seq(min(dat_temp$LST_Day_1km), max(dat_temp$LST_Day_1km), by=0.05)))

**for**(i **in** 1:length(**try**)) {

bin <- subset(dat_temp%>%filter(season_sex=="wet-female"), LST_Day_1km>**try**[i] & LST_Day_1km

<**try**[i]+0.05)

mean_slope <- mean(bin$n_per_year)

dat_made[i,1] <- mean_slope

}

dat_made <- as.data.frame(dat_made) dat_made <- cbind(dat_made, bins)

dat_made_wf <- dat_made %>% drop_na(V1)

*# Bulls, dry season*

dat_made <- data.frame()

**try** <- c(seq(min(dat_temp$LST_Day_1km), max(dat_temp$LST_Day_1km), by=0.05))

bins <- data.frame(LST_Day_1km = c(seq(min(dat_temp$LST_Day_1km), max(dat_temp$LST_Day_1km), by=0.05)))

**for**(i **in** 1:length(**try**)) {

bin <- subset(dat_temp%>%filter(season_sex=="dry-male"), LST_Day_1km>**try**[i] & LST_Day_1km<**t ry**[i]+0.05)

mean_slope <- mean(bin$n_per_year)

dat_made[i,1] <- mean_slope

}

dat_made <- as.data.frame(dat_made) dat_made <- cbind(dat_made, bins)

dat_made_dm <- dat_made %>% drop_na(V1)

*# Bulls, wet season*

dat_made <- data.frame()

**try** <- c(seq(min(dat_temp$LST_Day_1km), max(dat_temp$LST_Day_1km), by=0.05))

bins <- data.frame(LST_Day_1km = c(seq(min(dat_temp$LST_Day_1km), max(dat_temp$LST_Day_1km), by=0.05)))

**for**(i **in** 1:length(**try**)) {

bin <- subset(dat_temp%>%filter(season_sex=="wet-male"), LST_Day_1km>**try**[i] & LST_Day_1km<**t ry**[i]+0.05)

mean_slope <- mean(bin$n_per_year)

dat_made[i,1] <- mean_slope

}

dat_made <- as.data.frame(dat_made) dat_made <- cbind(dat_made, bins)

dat_made_wm <- dat_made %>% drop_na(V1)

*# Join data*

dat_made_wf <- dat_made_wf %>%

mutate(Group = "Cows, wet season") dat_made_df <- dat_made_df %>%

mutate(Group = "Cows, dry season") dat_made_wm <- dat_made_wm %>%

mutate(Group = "Bulls, wet season") dat_made_dm <- dat_made_dm %>%

mutate(Group = "Bulls, dry season")

sum_temp <- rbind(dat_made_wf, dat_made_df, dat_made_wm, dat_made_dm)

*# Plot*

ggplot() +

geom_point(data = sum_temp %>% filter(Group == "Bulls, wet season", V1<20), aes(x = LST_Day_1km, y = V1), color="#88CCEE", shape=24,

fill = "#88CCEE", alpha = 0.9, size=2) +

geom_point(data = sum_temp %>% filter(Group == "Bulls, dry season", V1<20), aes(x = LST_Day_1km, y = V1), color="#E65518", shape=24,

fill = "#E65518", alpha = 0.9, size=2) +

geom_point(data = sum_temp %>% filter(Group == "Cows, wet season", V1<20), aes(x = LST_Day_1km, y = V1), color="#009988", shape=21,

fill = "#009988", alpha = 0.9, size=2) +

geom_point(data = sum_temp %>% filter(Group == "Cows, dry season", V1<20), aes(x = LST_Day_1km, y = V1), color="#F2B701", shape=21,

fill = "#F2B701", alpha = 0.9, size=2) + labs(x = "temperature (°C)", y="revisitation rate") + theme(axis.title.x = element_text(size = 16),

axis.text.x = element_text(size = 14), axis.title.y = element_text(size = 16), axis.text.y = element_text(size = 14),

axis.line.x.bottom = element_line(colour = "black"), axis.line.y.left = element_line(colour = "black"),

axis.line.y.right = element_line(colour = "black"),

panel.background = element_rect(fill = "white"), panel.grid.major = element_line(color = "grey92"), panel.grid.minor = element_line(color = "grey92"))

*# Regression models*

sum_temp <- sum_temp %>% mutate(LST_Day_1km2 = LST_Day_1km^2)

*## Cows, dry season*

temp_mod <- sum_temp %>% filter(Group == "Cows, dry season") %>% mutate(n_year_log = log(V1))

my_recipe <- recipe(n_year_log ~ LST_Day_1km + LST_Day_1km2, data = temp_mod) my_recipe <- my_recipe %>% step_scale(all_numeric_predictors())

prep_recipe <- prep(my_recipe)

scaled_data <- bake(prep_recipe, new_data = temp_mod)

temp_model <- lm(n_year_log ~ LST_Day_1km + LST_Day_1km2, data = scaled_data) summary(temp_model)

*## Cows, wet season*

temp_mod <- sum_temp %>% filter(Group == "Cows, wet season") %>% mutate(n_year_log = log(V1))

my_recipe <- recipe(n_year_log ~ LST_Day_1km + LST_Day_1km2, data = temp_mod) my_recipe <- my_recipe %>% step_scale(all_numeric_predictors())

prep_recipe <- prep(my_recipe)

scaled_data <- bake(prep_recipe, new_data = temp_mod)

temp_model <- lm(n_year_log ~ LST_Day_1km + LST_Day_1km2, data = scaled_data) summary(temp_model)

*## Bulls, dry season*

temp_mod <- sum_temp %>% filter(Group == "Bulls, dry season") %>% mutate(n_year_log = log(V1))

my_recipe <- recipe(n_year_log ~ LST_Day_1km + LST_Day_1km2, data = temp_mod) my_recipe <- my_recipe %>% step_scale(all_numeric_predictors())

prep_recipe <- prep(my_recipe)

scaled_data <- bake(prep_recipe, new_data = temp_mod)

temp_model <- lm(n_year_log ~ LST_Day_1km + LST_Day_1km2, data = scaled_data) summary(temp_model)

*## Bulls, wet season*

temp_mod <- sum_temp %>% filter(Group == "Bulls, wet season") %>% mutate(n_year_log = log(V1))

my_recipe <- recipe(n_year_log ~ LST_Day_1km + LST_Day_1km2, data = temp_mod) my_recipe <- my_recipe %>% step_scale(all_numeric_predictors())

prep_recipe <- prep(my_recipe)

scaled_data <- bake(prep_recipe, new_data = temp_mod)

temp_model <- lm(n_year_log ~ LST_Day_1km + LST_Day_1km2, data = scaled_data) summary(temp_model)

# Model

*# Load data*

dat <- read_csv("Data/All_env_vars_tot_dat_with_temporal_all.csv")

*# Prepare dataset for model*

dat <- dat %>%

drop_na(n_per_year) %>% drop_na(LST_Day_1km)%>% drop_na(EVI) %>%

rename(temp = LST_Day_1km) %>% mutate(temp2 = temp^2,

sqrt_dist_water2 = sqrt_dist_water^2,

EVI2 = EVI^2,

log_slope2 = log_slope^2, phosphor2 = phosphor^2) %>%

dplyr::select(ID2, n_per_year, groupby_col, season, sex, season_sex, temp, temp2, EVI, EVI

2, log_slope,

log_slope2, phosphor, phosphor2, sqrt_dist_water, sqrt_dist_water2)

*# Save data*

write.csv("Data/All_env_vars_tot_dat_with_temporal_all_adjusted.csv")

*# Load data*

dat <- read_csv("Data/All_env_vars_tot_dat_with_temporal_all_adjusted.csv")

dat <- dat %>%

mutate(n_year_log = log(n_per_year))

## Cows, dry season

#### Prepare for resampling

*# Get cuts*

dat_cuts <- dat %>%

filter(season_sex =="dry-female") %>%

mutate(cut_evi = cut(EVI, breaks = seq(min(EVI), max(EVI), length.out = 10 + 1), labels = FALSE, include.lowest = TRUE),

cut_temp = cut(temp, breaks = seq(min(temp), max(temp), length.out = 10 + 1),

labels = FALSE, include.lowest = TRUE),

cut_slope = cut(log_slope, breaks = seq(min(log_slope), max(log_slope), length.out =

10 + 1),

+ 1),

labels = FALSE, include.lowest = TRUE),

cut_phos = cut(phosphor, breaks = seq(min(phosphor), max(phosphor), length.out = 10

labels = FALSE, include.lowest = TRUE),

cut_water = cut(sqrt_dist_water, breaks = seq(min(sqrt_dist_water), max(sqrt_dist_wa

ter), length.out = 10 + 1),

labels = FALSE, include.lowest = TRUE),

tot_cut_group = paste(cut_evi, cut_temp, cut_slope, cut_phos, cut_water, sep = "-"))

*# Get group counts*

count_groups <- dat_cuts %>% group_by(tot_cut_group) %>% summarise(count=n())

*# Get inverse of occurring*

dat_cuts <- dat_cuts %>%

left_join(count_groups, by = "tot_cut_group") %>% mutate(class_prob = 1/count)

glimpse(dat_cuts)

*# Calculate the sampling probabilities based on the inverse counts*

probs <- dat_cuts$class_prob / sum(dat_cuts$class_prob)

#### Model without interactions

*# Resample*

resampled_data <- dat_cuts[sample(nrow(dat_cuts), size = nrow(dat_cuts)/(nrow(dat_cuts)/200 0),

replace = T, prob = probs), ]

*# Fit model with all variables # scale data*

my_recipe <- recipe(n_year_log ~ temp + temp2 + EVI + EVI2 + log_slope +

log_slope2 + phosphor + phosphor2 + sqrt_dist_water + sqrt_dist_water2

+

groupby_col, data = resampled_data)

my_recipe <- my_recipe %>% step_scale(all_numeric_predictors()) prep_recipe <- prep(my_recipe)

scaled_data <- bake(prep_recipe, new_data = resampled_data)

*# Fit model*

full_model <- lmer(n_year_log ~ EVI + EVI2 + temp + temp2 + log_slope +

log_slope2 + phosphor + phosphor2 + sqrt_dist_water + sqrt_dist_water2 + (1|groupby_col), data = scaled_data, na.action = "na.fail", REML = F)

summary(full_model)

r.squaredGLMM(full_model)

*# Model selection*

model_step_wf <- dredge(full_model, trace = T)

*# Best model*

best_model <- lmer(n_year_log ~ EVI + EVI2 + temp + log_slope + phosphor + phosphor2 + sqrt_dist_water + sqrt_dist_water2 + (1|groupby_col),

data = scaled_data, na.action = "na.fail", REML = F)

summary(best_model)

r.squaredGLMM(best_model)

*# Get R2 best model*

*# Define the number of iterations*

n_iterations <- 10000

*# Create an empty vector to store the R2 values*

r2m <- numeric(n_iterations) r2c <- numeric(n_iterations)

r2_values <- as.data.frame(cbind(r2m, r2c))

**for** (i **in** 1:n_iterations) {

*# Get subsample*

resampled_data <- dat_cuts[sample(nrow(dat_cuts), size = nrow(dat_cuts)/(nrow(dat_cuts)/200 0),

replace = T, prob = probs), ]

*# Create the recipe*

my_recipe <- recipe(n_year_log ~ EVI + EVI2 + temp + log_slope + phosphor + phosphor2 +

sqrt_dist_water + sqrt_dist_water2 + groupby_col, data = resampled_data) my_recipe <- my_recipe %>% step_scale(all_numeric_predictors())

prep_recipe <- prep(my_recipe)

scaled_data <- bake(prep_recipe, new_data = resampled_data)

*# Fit the model*

best_model <- lmer(n_year_log ~ EVI + EVI2 + temp + log_slope + phosphor + phosphor2 + sqrt_dist_water + sqrt_dist_water2 + (1|groupby_col),

data = scaled_data, na.action = "na.fail", REML = F)

*# Calculate the R2 and store it in the vector*

r2_values[i,] <- r.squaredGLMM(best_model)

}

#### Interactions

*# resample data*

resampled_data <- dat_cuts[sample(nrow(dat_cuts), size = nrow(dat_cuts)/(nrow(dat_cuts)/1000 0),

replace = T, prob = probs), ]

my_recipe <- recipe(n_year_log ~ log_slope + phosphor + sqrt_dist_water +

EVI + temp + groupby_col, data = resampled_data) my_recipe <- my_recipe %>% step_scale(all_numeric_predictors())

prep_recipe <- prep(my_recipe)

scaled_data <- bake(prep_recipe, new_data = resampled_data)

*# Plot interactions*

model_int <- lm(n_year_log~phosphor*log_slope*EVI*sqrt_dist_water*temp, data=scaled_data) f1 <- interact_plot(model_int, pred = phosphor, modx = log_slope)

f2 <- interact_plot(model_int, pred = phosphor, modx = sqrt_dist_water) f3 <- interact_plot(model_int, pred = phosphor, modx = temp)

f4 <- interact_plot(model_int, pred = EVI, modx = phosphor) f5 <- interact_plot(model_int, pred = EVI, modx = log_slope)

f6 <- interact_plot(model_int, pred = EVI, modx = sqrt_dist_water)

f7 <- interact_plot(model_int, pred = EVI, modx = temp)

f8 <- interact_plot(model_int, pred = log_slope, modx = sqrt_dist_water) f9 <- interact_plot(model_int, pred = temp, modx = log_slope)

f10 <- interact_plot(model_int, pred = temp, modx = sqrt_dist_water)

gridExtra::grid.arrange(f1,f2,f3,f4,f5,f6,f7,f8,f9,f10)

#### Model with interactions

*# resample data*

resampled_data <- dat_cuts[sample(nrow(dat_cuts), size = nrow(dat_cuts)/(nrow(dat_cuts)/200 0),

replace = T, prob = probs), ]

*# Scale variables*

my_recipe <- recipe(n_year_log ~ EVI + EVI2 + temp + log_slope + phosphor + phosphor2 +

sqrt_dist_water + sqrt_dist_water2 + groupby_col, data = resampled_data) my_recipe <- my_recipe %>% step_scale(all_numeric_predictors())

prep_recipe <- prep(my_recipe)

scaled_data <- bake(prep_recipe, new_data = resampled_data)

*# Fit model*

full_model <- lmer(n_year_log ~ EVI + EVI2 + phosphor + phosphor2 + temp + log_slope + sqrt_dist_water + sqrt_dist_water2 + phosphor*log_slope +

log_slope*EVI + temp*log_slope + log_slope*sqrt_dist_water +

(1|groupby_col), data = scaled_data, na.action = "na.fail", REML = F)

summary(full_model)

r.squaredGLMM(full_model)

*# Model selection*

model_step_wf3 <- dredge(full_model, trace = T)

*# Best model*

best_model <- lmer(n_year_log ~ EVI + phosphor + phosphor2 + temp + log_slope + sqrt_dist_water + sqrt_dist_water2 + log_slope*EVI +

log_slope*sqrt_dist_water + (1|groupby_col),

data = scaled_data, na.action = "na.fail", REML = F)

summary(best_model)

r.squaredGLMM(best_model)

*## get R2 best model*

*# Define the number of iterations*

n_iterations <- 10000

*# Create an empty vector to store the R2 values*

r2m <- numeric(n_iterations) r2c <- numeric(n_iterations)

r2_values <- as.data.frame(cbind(r2m, r2c))

**for** (i **in** 1:n_iterations) {

*# Get subsample*

resampled_data <- dat_cuts[sample(nrow(dat_cuts), size = nrow(dat_cuts)/(nrow(dat_cuts)/200 0),

replace = T, prob = probs), ]

*# Create the recipe*

my_recipe <- recipe(n_year_log ~ EVI + EVI2 + phosphor + phosphor2 + temp + log_slope +

sqrt_dist_water + sqrt_dist_water2 + groupby_col, data = resampled_da

ta)

my_recipe <- my_recipe %>% step_scale(all_numeric_predictors()) prep_recipe <- prep(my_recipe)

scaled_data <- bake(prep_recipe, new_data = resampled_data)

*# Fit the model*

best_model <- lmer(n_year_log ~ EVI + phosphor + phosphor2 + temp + log_slope + sqrt_dist_water + sqrt_dist_water2 + log_slope*EVI +

log_slope*sqrt_dist_water + (1|groupby_col),

data = scaled_data, na.action = "na.fail", REML = F)

*# Calculate the R2 and store it in the vector*

r2_values[i,] <- r.squaredGLMM(best_model)

}

## Cows, wet season

#### Prepare for resampling

*# Get cuts*

dat_cuts <- dat %>%

filter(season_sex =="wet-female") %>%

mutate(cut_evi = cut(EVI, breaks = seq(min(EVI), max(EVI), length.out = 10 + 1), labels = FALSE, include.lowest = TRUE),

cut_temp = cut(temp, breaks = seq(min(temp), max(temp), length.out = 10 + 1),

labels = FALSE, include.lowest = TRUE),

cut_slope = cut(log_slope, breaks = seq(min(log_slope), max(log_slope), length.out =

10 + 1),

+ 1),

labels = FALSE, include.lowest = TRUE),

cut_phos = cut(phosphor, breaks = seq(min(phosphor), max(phosphor), length.out = 10

labels = FALSE, include.lowest = TRUE),

cut_water = cut(sqrt_dist_water, breaks = seq(min(sqrt_dist_water), max(sqrt_dist_wa

ter), length.out = 10 + 1),

labels = FALSE, include.lowest = TRUE),

tot_cut_group = paste(cut_evi, cut_temp, cut_slope, cut_phos, cut_water, sep = "-"))

*# Get group counts*

count_groups <- dat_cuts %>% group_by(tot_cut_group) %>% summarise(count=n())

*# Get inverse of occurring*

dat_cuts <- dat_cuts %>%

left_join(count_groups, by = "tot_cut_group") %>% mutate(class_prob = 1/count)

glimpse(dat_cuts)

*# Calculate the sampling probabilities based on the inverse counts*

probs <- dat_cuts$class_prob / sum(dat_cuts$class_prob)

#### Model without interactions

*# Resample*

resampled_data <- dat_cuts[sample(nrow(dat_cuts), size = nrow(dat_cuts)/(nrow(dat_cuts)/200 0),

replace = T, prob = probs), ]

*# Fit model with all variables # scale data*

my_recipe <- recipe(n_year_log ~ temp + temp2 + EVI + EVI2 + log_slope +

log_slope2 + phosphor + phosphor2 + sqrt_dist_water + sqrt_dist_water2

+

groupby_col, data = resampled_data)

my_recipe <- my_recipe %>% step_scale(all_numeric_predictors()) prep_recipe <- prep(my_recipe)

scaled_data <- bake(prep_recipe, new_data = resampled_data)

*# Fit model*

full_model <- lmer(n_year_log ~ EVI + EVI2 + temp + temp2 + log_slope +

log_slope2 + phosphor + phosphor2 + sqrt_dist_water + sqrt_dist_water2 + (1|groupby_col), data = scaled_data, na.action = "na.fail", REML = F)

summary(full_model)

r.squaredGLMM(full_model)

*# Model selection*

model_step_wf <- dredge(full_model, trace = T)

*# Best model*

best_model <- lmer(n_year_log ~ EVI + log_slope + log_slope2 + phosphor + phosphor2 + sqrt_dist_water + (1|groupby_col),

data = scaled_data, na.action = "na.fail", REML = F)

summary(best_model)

r.squaredGLMM(best_model)

*# Get R2 best model*

*# Define the number of iterations*

n_iterations <- 10000

*# Create an empty vector to store the R2 values*

r2m <- numeric(n_iterations) r2c <- numeric(n_iterations)

r2_values <- as.data.frame(cbind(r2m, r2c))

**for** (i **in** 1:n_iterations) {

*# Get subsample*

resampled_data <- dat_cuts[sample(nrow(dat_cuts), size = nrow(dat_cuts)/(nrow(dat_cuts)/200 0),

replace = T, prob = probs), ]

*# Create the recipe*

my_recipe <- recipe(n_year_log ~ EVI + log_slope + log_slope2 + phosphor +

phosphor2 + sqrt_dist_water + groupby_col, data = resampled_data) my_recipe <- my_recipe %>% step_scale(all_numeric_predictors())

prep_recipe <- prep(my_recipe)

scaled_data <- bake(prep_recipe, new_data = resampled_data)

*# Fit the model*

best_model <- lmer(n_year_log ~ EVI + log_slope + log_slope2 + phosphor + phosphor2 + sqrt_dist_water + (1|groupby_col),

data = scaled_data, na.action = "na.fail", REML = F)

*# Calculate the R2 and store it in the vector*

r2_values[i,] <- r.squaredGLMM(best_model)

}

#### Interactions

*# resample data*

resampled_data <- dat_cuts[sample(nrow(dat_cuts), size = nrow(dat_cuts)/(nrow(dat_cuts)/1000 0),

replace = T, prob = probs), ]

my_recipe <- recipe(n_year_log ~ log_slope + phosphor + sqrt_dist_water +

EVI + groupby_col, data = resampled_data) my_recipe <- my_recipe %>% step_scale(all_numeric_predictors()) prep_recipe <- prep(my_recipe)

scaled_data <- bake(prep_recipe, new_data = resampled_data)

*# Plot interactions*

model_int <- lm(n_year_log~phosphor*log_slope*EVI*sqrt_dist_water*temp, data=scaled_data) f1 <- interact_plot(model_int, pred = phosphor, modx = log_slope)

f2 <- interact_plot(model_int, pred = phosphor, modx = sqrt_dist_water) f3 <- interact_plot(model_int, pred = EVI, modx = phosphor)

f4 <- interact_plot(model_int, pred = EVI, modx = log_slope)

f5 <- interact_plot(model_int, pred = EVI, modx = sqrt_dist_water)

f6 <- interact_plot(model_int, pred = log_slope, modx = sqrt_dist_water) gridExtra::grid.arrange(f1,f2,f3,f4,f5,f6)

Model with interactions

*# resample data*

resampled_data <- dat_cuts[sample(nrow(dat_cuts), size = nrow(dat_cuts)/(nrow(dat_cuts)/200 0),

replace = T, prob = probs), ]

*# Scale variables*

my_recipe <- recipe(n_year_log ~ EVI + log_slope + log_slope2 + phosphor +

phosphor2 + sqrt_dist_water + groupby_col, data = resampled_data) my_recipe <- my_recipe %>% step_scale(all_numeric_predictors())

prep_recipe <- prep(my_recipe)

scaled_data <- bake(prep_recipe, new_data = resampled_data)

*# Fit model*

full_model <- lmer(n_year_log ~ EVI + log_slope + log_slope2 + phosphor +

phosphor2 + sqrt_dist_water + EVI*phosphor + EVI*log_slope + log_slope*sqrt_dist_water + log_slope*phosphor +

(1|groupby_col), data = scaled_data, na.action = "na.fail", REML = F)

summary(full_model)

r.squaredGLMM(full_model)

*# Model selection*

model_step_wf3 <- dredge(full_model, trace = T)

*# Best model*

best_model <- lmer(n_year_log ~ EVI + log_slope + log_slope2 + phosphor +

phosphor2 + sqrt_dist_water + log_slope*sqrt_dist_water +

(1|groupby_col), data = scaled_data, na.action = "na.fail", REML = F)

summary(best_model)

r.squaredGLMM(best_model)

*## get R2 best model*

*# Define the number of iterations*

n_iterations <- 10000

*# Create an empty vector to store the R2 values*

r2m <- numeric(n_iterations) r2c <- numeric(n_iterations)

r2_values <- as.data.frame(cbind(r2m, r2c))

**for** (i **in** 1:n_iterations) {

*# Get subsample*

resampled_data <- dat_cuts[sample(nrow(dat_cuts), size = nrow(dat_cuts)/(nrow(dat_cuts)/200 0),

replace = T, prob = probs), ]

*# Create the recipe*

my_recipe <- recipe(n_year_log ~ EVI + log_slope + log_slope2 + phosphor +

phosphor2 + sqrt_dist_water + groupby_col, data = resampled_data) my_recipe <- my_recipe %>% step_scale(all_numeric_predictors())

prep_recipe <- prep(my_recipe)

scaled_data <- bake(prep_recipe, new_data = resampled_data)

*# Fit the model*

best_model <- lmer(n_year_log ~ EVI + log_slope + log_slope2 + phosphor +

phosphor2 + sqrt_dist_water + log_slope*sqrt_dist_water +

(1|groupby_col), data = scaled_data, na.action = "na.fail", REML = F)

*# Calculate the R2 and store it in the vector*

r2_values[i,] <- r.squaredGLMM(best_model)

}

## Bulls, dry season

#### Prepare for resampling

*# Get cuts*

dat_cuts <- dat %>%

filter(season_sex =="dry-male") %>%

mutate(cut_evi = cut(EVI, breaks = seq(min(EVI), max(EVI), length.out = 10 + 1), labels = FALSE, include.lowest = TRUE),

cut_temp = cut(temp, breaks = seq(min(temp), max(temp), length.out = 10 + 1),

labels = FALSE, include.lowest = TRUE),

cut_slope = cut(log_slope, breaks = seq(min(log_slope), max(log_slope), length.out =

10 + 1),

+ 1),

labels = FALSE, include.lowest = TRUE),

cut_phos = cut(phosphor, breaks = seq(min(phosphor), max(phosphor), length.out = 10

labels = FALSE, include.lowest = TRUE),

cut_water = cut(sqrt_dist_water, breaks = seq(min(sqrt_dist_water), max(sqrt_dist_wa

ter), length.out = 10 + 1),

labels = FALSE, include.lowest = TRUE),

tot_cut_group = paste(cut_evi, cut_temp, cut_slope, cut_phos, cut_water, sep = "-"))

*# Get group counts*

count_groups <- dat_cuts %>% group_by(tot_cut_group) %>% summarise(count=n())

*# Get inverse of occurring*

dat_cuts <- dat_cuts %>%

left_join(count_groups, by = "tot_cut_group") %>% mutate(class_prob = 1/count)

glimpse(dat_cuts)

*# Calculate the sampling probabilities based on the inverse counts*

probs <- dat_cuts$class_prob / sum(dat_cuts$class_prob)

#### Model without interactions

*# Resample*

resampled_data <- dat_cuts[sample(nrow(dat_cuts), size = nrow(dat_cuts)/(nrow(dat_cuts)/200 0),

replace = T, prob = probs), ]

*# Fit model with all variables # scale data*

my_recipe <- recipe(n_year_log ~ temp + temp2 + EVI + EVI2 + log_slope +

log_slope2 + phosphor + phosphor2 + sqrt_dist_water + sqrt_dist_water2

+

groupby_col, data = resampled_data)

my_recipe <- my_recipe %>% step_scale(all_numeric_predictors()) prep_recipe <- prep(my_recipe)

scaled_data <- bake(prep_recipe, new_data = resampled_data)

*# Fit model*

full_model <- lmer(n_year_log ~ EVI + EVI2 + temp + temp2 + log_slope +

log_slope2 + phosphor + phosphor2 + sqrt_dist_water + sqrt_dist_water2 + (1|groupby_col), data = scaled_data, na.action = "na.fail", REML = F)

summary(full_model)

r.squaredGLMM(full_model)

*# Model selection*

model_step_wf <- dredge(full_model, trace = T)

*# Best model*

best_model <- lmer(n_year_log ~ EVI + EVI2 + log_slope + log_slope2 +

sqrt_dist_water + sqrt_dist_water2 + (1|groupby_col), data = scaled_data, na.action = "na.fail", REML = F)

summary(best_model)

r.squaredGLMM(best_model)

*# Get R2 best model*

*# Define the number of iterations*

n_iterations <- 10000

*# Create an empty vector to store the R2 values*

r2m <- numeric(n_iterations) r2c <- numeric(n_iterations)

r2_values <- as.data.frame(cbind(r2m, r2c))

**for** (i **in** 1:n_iterations) {

*# Get subsample*

resampled_data <- dat_cuts[sample(nrow(dat_cuts), size = nrow(dat_cuts)/(nrow(dat_cuts)/200 0),

replace = T, prob = probs), ]

*# Create the recipe*

my_recipe <- recipe(n_year_log ~ EVI + EVI2 + log_slope + log_slope2 +

sqrt_dist_water + sqrt_dist_water2 + groupby_col, data = resampled_data) my_recipe <- my_recipe %>% step_scale(all_numeric_predictors())

prep_recipe <- prep(my_recipe)

scaled_data <- bake(prep_recipe, new_data = resampled_data)

*# Fit the model*

best_model <- lmer(n_year_log ~ EVI + EVI2 + log_slope + log_slope2 +

sqrt_dist_water + sqrt_dist_water2 + (1|groupby_col), data = scaled_data, na.action = "na.fail", REML = F)

*# Calculate the R2 and store it in the vector*

r2_values[i,] <- r.squaredGLMM(best_model)

}

#### Interactions

*# resample data*

resampled_data <- dat_cuts[sample(nrow(dat_cuts), size = nrow(dat_cuts)/(nrow(dat_cuts)/1000 0),

replace = T, prob = probs), ]

my_recipe <- recipe(n_year_log ~ log_slope + sqrt_dist_water +

EVI + groupby_col, data = resampled_data) my_recipe <- my_recipe %>% step_scale(all_numeric_predictors()) prep_recipe <- prep(my_recipe)

scaled_data <- bake(prep_recipe, new_data = resampled_data)

*# Plot interactions*

model_int <- lm(n_year_log~phosphor*log_slope*EVI*sqrt_dist_water*temp, data=scaled_data) f1 <- interact_plot(model_int, pred = EVI, modx = sqrt_dist_water)

f2 <- interact_plot(model_int, pred = EVI, modx = log_slope)

f3 <- interact_plot(model_int, pred = log_slope, modx = sqrt_dist_water) gridExtra::grid.arrange(f1,f2,f3)

Model with interactions

*# resample data*

resampled_data <- dat_cuts[sample(nrow(dat_cuts), size = nrow(dat_cuts)/(nrow(dat_cuts)/200 0),

replace = T, prob = probs), ]

*# Scale variables*

my_recipe <- recipe(n_year_log ~ EVI + EVI2 + log_slope + log_slope2 +

sqrt_dist_water + sqrt_dist_water2 + groupby_col, data = resampled_data) my_recipe <- my_recipe %>% step_scale(all_numeric_predictors())

prep_recipe <- prep(my_recipe)

scaled_data <- bake(prep_recipe, new_data = resampled_data)

*# Fit model*

full_model <- lmer(n_year_log ~ EVI + EVI2 + log_slope + log_slope2 + sqrt_dist_water +

sqrt_dist_water2 + sqrt_dist_water*EVI + sqrt_dist_water*log_slope + (1|groupby_col), data = scaled_data, na.action = "na.fail", REML = F)

summary(full_model)

r.squaredGLMM(full_model)

*# Model selection*

model_step_wf3 <- dredge(full_model, trace = T)

*# Best model*

best_model <- lmer(n_year_log ~ EVI + EVI2 + log_slope + log_slope2 + sqrt_dist_water + sqrt_dist_water2 + sqrt_dist_water*log_slope + (1|groupby_col),

data = scaled_data, na.action = "na.fail", REML = F)

summary(best_model)

r.squaredGLMM(best_model)

*## get R2 best model*

*# Define the number of iterations*

n_iterations <- 10000

*# Create an empty vector to store the R2 values*

r2m <- numeric(n_iterations) r2c <- numeric(n_iterations)

r2_values <- as.data.frame(cbind(r2m, r2c))

**for** (i **in** 1:n_iterations) {

*# Get subsample*

resampled_data <- dat_cuts[sample(nrow(dat_cuts), size = nrow(dat_cuts)/(nrow(dat_cuts)/200 0),

replace = T, prob = probs), ]

*# Create the recipe*

my_recipe <- recipe(n_year_log ~ EVI + EVI2 + log_slope + log_slope2 + sqrt_dist_water + sqrt_dist_water2 + groupby_col, data = resampled_data)

my_recipe <- my_recipe %>% step_scale(all_numeric_predictors())

prep_recipe <- prep(my_recipe)

scaled_data <- bake(prep_recipe, new_data = resampled_data)

*# Fit the model*

best_model <- lmer(n_year_log ~ EVI + EVI2 + log_slope + log_slope2 + sqrt_dist_water + sqrt_dist_water2 + sqrt_dist_water*log_slope + (1|groupby_col),

data = scaled_data, na.action = "na.fail", REML = F)

*# Calculate the R2 and store it in the vector*

r2_values[i,] <- r.squaredGLMM(best_model)

}

## Bulls, wet season

#### Prepare for resampling

*# Get cuts*

dat_cuts <- dat %>%

filter(season_sex =="wet-male") %>%

mutate(cut_evi = cut(EVI, breaks = seq(min(EVI), max(EVI), length.out = 10 + 1), labels = FALSE, include.lowest = TRUE),

cut_temp = cut(temp, breaks = seq(min(temp), max(temp), length.out = 10 + 1),

labels = FALSE, include.lowest = TRUE),

cut_slope = cut(log_slope, breaks = seq(min(log_slope), max(log_slope), length.out =

10 + 1),

+ 1),

labels = FALSE, include.lowest = TRUE),

cut_phos = cut(phosphor, breaks = seq(min(phosphor), max(phosphor), length.out = 10

labels = FALSE, include.lowest = TRUE),

cut_water = cut(sqrt_dist_water, breaks = seq(min(sqrt_dist_water), max(sqrt_dist_wa

ter), length.out = 10 + 1),

labels = FALSE, include.lowest = TRUE),

tot_cut_group = paste(cut_evi, cut_temp, cut_slope, cut_phos, cut_water, sep = "-"))

*# Get group counts*

count_groups <- dat_cuts %>% group_by(tot_cut_group) %>% summarise(count=n())

*# Get inverse of occurring*

dat_cuts <- dat_cuts %>%

left_join(count_groups, by = "tot_cut_group") %>% mutate(class_prob = 1/count)

glimpse(dat_cuts)

*# Calculate the sampling probabilities based on the inverse counts*

probs <- dat_cuts$class_prob / sum(dat_cuts$class_prob)

#### Model without interactions

*# Resample*

resampled_data <- dat_cuts[sample(nrow(dat_cuts), size = nrow(dat_cuts)/(nrow(dat_cuts)/200 0),

replace = T, prob = probs), ]

*# Fit model with all variables # scale data*

my_recipe <- recipe(n_year_log ~ temp + temp2 + EVI + EVI2 + log_slope +

log_slope2 + phosphor + phosphor2 + sqrt_dist_water + sqrt_dist_water2

+

groupby_col, data = resampled_data)

my_recipe <- my_recipe %>% step_scale(all_numeric_predictors()) prep_recipe <- prep(my_recipe)

scaled_data <- bake(prep_recipe, new_data = resampled_data)

*# Fit model*

full_model <- lmer(n_year_log ~ EVI + EVI2 + temp + temp2 + log_slope +

log_slope2 + phosphor + phosphor2 + sqrt_dist_water + sqrt_dist_water2 + (1|groupby_col), data = scaled_data, na.action = "na.fail", REML = F)

summary(full_model)

r.squaredGLMM(full_model)

*# Model selection*

model_step_wf <- dredge(full_model, trace = T)

*# Best model*

best_model <- lmer(n_year_log ~ EVI + EVI2 + temp + log_slope + log_slope2 +

phosphor + phosphor2 + sqrt_dist_water + sqrt_dist_water2 +

(1|groupby_col), data = scaled_data, na.action = "na.fail", REML = F)

summary(best_model)

r.squaredGLMM(best_model)

*# Get R2 best model*

*# Define the number of iterations*

n_iterations <- 10000

*# Create an empty vector to store the R2 values*

r2m <- numeric(n_iterations) r2c <- numeric(n_iterations)

r2_values <- as.data.frame(cbind(r2m, r2c))

**for** (i **in** 1:n_iterations) {

*# Get subsample*

resampled_data <- dat_cuts[sample(nrow(dat_cuts), size = nrow(dat_cuts)/(nrow(dat_cuts)/200 0),

replace = T, prob = probs), ]

*# Create the recipe*

my_recipe <- recipe(n_year_log ~ EVI + EVI2 + temp + log_slope + log_slope2 +

phosphor + phosphor2 + sqrt_dist_water + sqrt_dist_water2 + groupby_col, data = resampled_data)

my_recipe <- my_recipe %>% step_scale(all_numeric_predictors())

prep_recipe <- prep(my_recipe)

scaled_data <- bake(prep_recipe, new_data = resampled_data)

*# Fit the model*

best_model <- lmer(n_year_log ~ EVI + EVI2 + temp + log_slope + log_slope2 +

phosphor + phosphor2 + sqrt_dist_water + sqrt_dist_water2 +

(1|groupby_col), data = scaled_data, na.action = "na.fail", REML = F)

*# Calculate the R2 and store it in the vector*

r2_values[i,] <- r.squaredGLMM(best_model)

}

#### Interactions

*# resample data*

resampled_data <- dat_cuts[sample(nrow(dat_cuts), size = nrow(dat_cuts)/(nrow(dat_cuts)/1000 0),

replace = T, prob = probs), ]

my_recipe <- recipe(n_year_log ~ log_slope + phosphor + sqrt_dist_water +

EVI + temp + groupby_col, data = resampled_data) my_recipe <- my_recipe %>% step_scale(all_numeric_predictors())

prep_recipe <- prep(my_recipe)

scaled_data <- bake(prep_recipe, new_data = resampled_data)

*# Plot interactions*

model_int <- lm(n_year_log~phosphor*log_slope*EVI*sqrt_dist_water*temp, data=scaled_data) f1 <- interact_plot(model_int, pred = phosphor, modx = log_slope)

f2 <- interact_plot(model_int, pred = phosphor, modx = sqrt_dist_water) f3 <- interact_plot(model_int, pred = phosphor, modx = temp)

f4 <- interact_plot(model_int, pred = EVI, modx = phosphor) f4 <- interact_plot(model_int, pred = EVI, modx = log_slope)

f6 <- interact_plot(model_int, pred = EVI, modx = sqrt_dist_water)

f7 <- interact_plot(model_int, pred = EVI, modx = temp)

f8 <- interact_plot(model_int, pred = log_slope, modx = sqrt_dist_water) f9 <- interact_plot(model_int, pred = temp, modx = log_slope)

f10 <- interact_plot(model_int, pred = temp, modx = sqrt_dist_water)

gridExtra::grid.arrange(f1,f2,f3,f4,f5,f6,f7,f8,f9,f10)

#### Model with interactions

*# resample data*

resampled_data <- dat_cuts[sample(nrow(dat_cuts), size = nrow(dat_cuts)/(nrow(dat_cuts)/200 0),

replace = T, prob = probs), ]

*# Scale variables*

my_recipe <- recipe(n_year_log ~ EVI + EVI2 + temp + log_slope + log_slope2 +

phosphor + phosphor2 + sqrt_dist_water + sqrt_dist_water2 + groupby_col, data = resampled_data)

my_recipe <- my_recipe %>% step_scale(all_numeric_predictors()) prep_recipe <- prep(my_recipe)

scaled_data <- bake(prep_recipe, new_data = resampled_data)

*# Fit model*

full_model <- lmer(n_year_log ~ EVI + EVI2 + temp + log_slope + log_slope2 +

phosphor + phosphor2 + sqrt_dist_water + sqrt_dist_water2 + EVI*phosphor

+

EVI*sqrt_dist_water + phosphor*sqrt_dist_water + log_slope*temp +

(1|groupby_col), data = scaled_data, na.action = "na.fail", REML = F)

summary(full_model)

r.squaredGLMM(full_model)

*# Model selection*

model_step_wf3 <- dredge(full_model, trace = T)

*# Best model*

best_model <- lmer(n_year_log ~ EVI + EVI2 + temp + log_slope + log_slope2 +

phosphor + phosphor2 + sqrt_dist_water + sqrt_dist_water2 + EVI*phosphor + phosphor*sqrt_dist_water + (1|groupby_col), data = scaled_data, na.action = "na.fail", REML = F)

summary(best_model)

r.squaredGLMM(best_model)

*## get R2 best model*

*# Define the number of iterations*

n_iterations <- 10000

*# Create an empty vector to store the R2 values*

r2m <- numeric(n_iterations) r2c <- numeric(n_iterations)

r2_values <- as.data.frame(cbind(r2m, r2c))

**for** (i **in** 1:n_iterations) {

*# Get subsample*

resampled_data <- dat_cuts[sample(nrow(dat_cuts), size = nrow(dat_cuts)/(nrow(dat_cuts)/200 0),

replace = T, prob = probs), ]

*# Create the recipe*

my_recipe <- recipe(n_year_log ~ EVI + EVI2 + temp + log_slope + log_slope2 +

phosphor + phosphor2 + sqrt_dist_water + sqrt_dist_water2 + groupby_col, data = resampled_data)

my_recipe <- my_recipe %>% step_scale(all_numeric_predictors())

prep_recipe <- prep(my_recipe)

scaled_data <- bake(prep_recipe, new_data = resampled_data)

*# Fit the model*

best_model <- lmer(n_year_log ~ EVI + EVI2 + temp + log_slope + log_slope2 + phosphor + phosphor2 + sqrt_dist_water + sqrt_dist_water2 + EVI*phosphor + phosphor*sqrt_dist_water + (1|groupby_col), data = scaled_data, na.action = "na.fail", REML = F)

*# Calculate the R2 and store it in the vector*

r2_values[i,] <- r.squaredGLMM(best_model)

}
